# *P*-adic *L*-functions for $\textrm{GL}(3)$

**DOI:** 10.1007/s00208-026-03377-w

**Published:** 2026-03-11

**Authors:** David Loeffler, Chris Williams

**Affiliations:** 1 University of Warwick, Mathematics Institute, Zeeman Building, CV4 7AL Coventry, UK; 2 Present Address: UniDistance Suisse, Schinerstrasse 18, 3900 Brig, Switzerland; 3 University of Nottingham, School of Mathematical Sciences, NG7 2RD Nottingham, UK

## Abstract

Let $\Pi$ be a regular algebraic cuspidal automorphic representation (RACAR) of $\textrm{GL}_3(\mathbb {A}_{\mathbb {Q}})$. When $\Pi$ is *p*-nearly-ordinary for the maximal standard parabolic with Levi $\textrm{GL}_1 \times \textrm{GL}_2$, we construct a *p*-adic *L*-function for $\Pi$. More precisely, we construct a (single) bounded measure $L_p(\Pi )$ on $\mathbb {Z}_p^{\times }$ attached to $\Pi$, and show it interpolates all the critical values $L(\Pi \times \eta ,-j)$ at *p* in the left-half of the critical strip for $\Pi$ (for varying $\eta$ and *j*). This proves conjectures of Coates–Perrin-Riou and Panchishkin in this case. We also prove a corresponding result in the right half of the critical strip, assuming near-ordinarity for the other maximal standard parabolic. Our construction uses the theory of spherical varieties to build a “Betti Euler system”, a norm-compatible system of classes in the Betti cohomology of a locally symmetric space for $\textrm{GL}_3$. We work in arbitrary cohomological weight, allow arbitrary ramification at *p* along the Levi factor of the standard parabolic, and make no self-duality assumption. We thus give the first constructions of *p*-adic *L*-functions for RACARs of $\textrm{GL}_n(\mathbb {A}_{\mathbb {Q}})$ of ‘general type’ (i.e. those that do not arise as functorial lifts) for any $n >2$.

## Introduction

### Context

There is a wide network of conjectures, including the Birch–Swinnerton-Dyer and Bloch–Kato conjectures, that describe important arithmetic invariants in terms of special values of (complex) *L*-functions. One of the most successful tools in tackling these conjectures has been Iwasawa theory, in which one seeks to formulate analogous *p*-adic conjectures, replacing the complex analytic *L*-function with a *p*-*adic* *L-function*. The resulting *p*-adic Iwasawa main conjectures are often more tractable than their complex counterparts, and provide beautiful, deep connections between analysis and arithmetic.

A crucial launching point for Iwasawa theory is proving *existence* of *p*-adic *L*-functions. Coates and Perrin-Riou conjectured the existence of a *p*-adic *L*-function attached to every motive *M* over $\mathbb {Q}$ that is ordinary with good reduction at *p*, and whose *L*-function has at least one critical value [9, 10]. This itself rests on Deligne’s period conjecture [12]. One may formulate automorphic realisations of these (motivic) conjectures, but proving these remains very difficult.

To illustrate this, consider the fundamental case where $\Pi$ is a regular algebraic cuspidal automorphic representation (RACAR) of $\textrm{GL}_n(\mathbb {A}_{\mathbb {Q}})$. Then Coates and Perrin-Riou predict that when $\Pi$ is unramified and ordinary at *p*, there exists a *p*-adic measure on $\mathbb {Z}_p^{\times }$ – the *p*-adic *L*-function of $\Pi$ – interpolating all its critical *L*-values at *p*; we recall the precise conjecture below.

For $n=1,2$, existence of *p*-adic *L*-functions has been known for decades (starting from e.g. [35, 48]). For $n \ge 3$, however, our understanding of the conjecture remains poor, and it is known only in very special cases, e.g. symmetric squares of classical modular forms [11, 23, 56], RACARs of $\textrm{GL}_{2n}$ with Shalika models [1, 13, 16], or Rankin–Selberg transfers from $\textrm{GL}_n \times \textrm{GL}_{n+1}$ to $\textrm{GL}_{n(n+1)}$ [28, 30, 57]. In particular, in *all* known cases, $\Pi$ arises as a functorial lift from a group whose *L*-group is a proper subgroup of $\textrm{GL}_n$: beyond the classical cases of $n=1, 2$, there are no known constructions applying to automorphic representations $\Pi$ of ‘general type’, i.e. not arising from a smaller group in this way.

In this paper, we prove existence of *p*-adic *L*-functions for *p*-ordinary RACARs of $\textrm{GL}_3(\mathbb {A}_{\mathbb {Q}})$, making no self-duality or functorial lift assumptions. This provides the first examples of *p*-adic *L*-functions for general type RACARs of $\textrm{GL}_n(\mathbb {A}_{\mathbb {Q}})$ for any $n > 2$.

We briefly highlight some further strengths of our construction:

- We work in arbitrary cohomological weight, and prove the so-called ‘Manin relations’; that is, for each critical region we construct a *single* *p*-adic measure that sees *L*-values at all critical integers (rather than a separate measure for each different critical integer).
- We actually prove a more general conjecture of Panchishkin [50] that refines Coates–Perrin-Riou: rather than Coates–Perrin-Riou’s assumption of Borel-ordinarity at *p*, we impose *p*-ordinarity only along either the maximal standard parabolic $P_1$ with Levi $\textrm{GL}_1 \times \textrm{GL}_2$, or $P_2$ with Levi $\textrm{GL}_2\times \textrm{GL}_1$. Additionally, we give constructions (in both cases) assuming only near-ordinarity, hence extending [50] beyond the ordinary case.
- We allow arbitrary ramification at *p*. As a result, our construction applies even to a class of RACARs that have infinite slope at *p* for the Borel subgroup (those that are nearly ordinary for $P_1$, but have infinite slope along the other maximal parabolic subgroup $P_2$; or vice versa).

We believe that our result is “optimal”, in the sense that *p*-adic *L*-functions for RACARs of $\textrm{GL}_3$ should only exist as bounded measures when these conditions hold. (Our methods can be adapted to construct finite-order tempered distributions interpolating *L*-values of RACARs which have positive, but sufficiently small, slope for $P_i$; this will be pursued elsewhere.)

### Our results

To motivate the precise form of our main result, we first state Panchishkin’s refinement of the Coates–Perrin-Riou conjecture for $\textrm{GL}_n(\mathbb {A}_{\mathbb {Q}})$.

Let $n = 2m+1$ be odd.^1^ Let $\Pi$ be a (unitary^2^) RACAR of $\textrm{GL}_{n}(\mathbb {A}_{\mathbb {Q}})$ with central character $\omega _\Pi$ and weight $\lambda = (\lambda _1,...,\lambda _{2m+1})$. Note $\lambda _i \ge \lambda _{i+1}$, $\lambda _m = 0$, and $\lambda _i = -\lambda _{2\,m+1-i}$. With these notations, for $\eta$ a Dirichlet character and $t \in \mathbb {Z}$, the *L*-value $L(\Pi \times \eta , t)$ is critical for $(t, \eta )$ lying in one of the following sets:

$$\begin{aligned} {{\,\textrm{Crit}\,}}^-(\Pi )&= \{(-j,\eta ) \ \ \ : 0 \le j \le \lambda _{m-1}, \ \ \ \eta \omega _{\Pi }(-1) = (-1)^{j}\},\\ {{\,\textrm{Crit}\,}}^+(\Pi )&= \{(j+1,\eta ) : 0\le j \le \lambda _{m-1},\ \ \ \eta \omega _{\Pi }(-1) = (-1)^{j}\} \end{aligned}$$

See Proposition 2.4. These are the critical values of twists of $L(\Pi ,s)$ in the left and right halves of the critical strip respectively. We write ${{\,\textrm{Crit}\,}}(\Pi )$ for the union of these sets. For $(t, \eta ) \in {{\,\textrm{Crit}\,}}(\Pi )$, let $e_\infty (\Pi _\infty \times \eta _\infty , t)$ be the modified Euler factor at $\infty$ of [9, §1], which is a product of a rational number with powers of *i* and $\pi$ (see Sect. 2.5).

#### Algebraicity

As a precursor to *p*-adic interpolation, we first consider an algebraicity result for *L*-values. Let *E* denote the rationality field of $\Pi$. If $\eta$ is a Dirichlet character, let $E[\eta ]$ denote the extension of *E* obtained by the values of $\eta$. The following is [9, Period Conjecture], a reformulation of the conjectures of [12] better suited to *p*-adic interpolation:

##### Conjecture 1.1

(Algebraicity Conjecture for $\textrm{GL}_{2m+1}$) There exist complex periods $\Omega _\Pi ^\pm \in \mathbb {C}^{\times }$ such that for all $(t,\eta ) \in {{\,\textrm{Crit}\,}}^\pm (\Pi )$, we have

1.1 $$\begin{aligned} e_\infty \left( \Pi _\infty \times \eta _\infty , t\right) \cdot \frac{L(\Pi \times \eta , t)}{\Omega _\Pi ^\pm } \cdot G(\eta ^{-1})^{(n \pm 1)/2} \in E[\eta ], \end{aligned}$$

where $G(\eta )$ is the Gauss sum. Moreover, this ratio depends $\operatorname {Gal}(E[\eta ] / E)$-equivariantly on $\eta$.

Note that it suffices to prove the conjecture (for all $\Pi$) for one choice of the sign ±, since the functional equation interchanges ${{\,\textrm{Crit}\,}}^{\pm }(\Pi )$ and ${{\,\textrm{Crit}\,}}^{\mp }(\Pi ^\vee )$; see Sect. 10 below. A partial result towards this conjecture is known for $n = 3$, by work of Mahnkopf and Kasten–Schmidt (see Sect. 1.3). Our first main result is:

##### Theorem A

Conjecture 1.1 holds for $n = 3$.

#### P-adic interpolation

Now suppose *p* is a prime. The second conjecture we state builds on the algebraicity conjecture 1.1 by predicting the algebraic parts of *L*-values vary *p*-adic analytically as we deform $(\eta ,j)$. It implies that these values satisfy remarkable *p*-adic congruences, akin to higher-dimensional analogues of the famous Kummer congruences for the Riemann zeta function.

Let $P_i$ be the block-upper-triangular parabolic subgroup with Levi subgroup $\textrm{GL}_i \times \textrm{GL}_{n-i}$. A $P_i$*-refinement* is a choice of irreducible representation of $\textrm{GL}_i \times \textrm{GL}_{n-i}$ appearing in the Jacquet module $J_{P_i}(\Pi _p)$. We define in Sect. 2.6 below a notion of *slope* for $P_i$-refinements, and we say $\Pi$ is $P_i$*-nearly-ordinary* if it admits a $P_i$-refinement of the minimal possible slope. We are most interested in $i = m, m+1$.

Let ${{\,\textrm{Crit}\,}}_p^\pm (\Pi ) \subset {{\,\textrm{Crit}\,}}^\pm (\Pi )$ be the subset of $(t, \eta )$ where $\eta$ has *p*-power conductor. For such $(t, \eta )$, let $e_p(\Pi _p \times \eta _p, t) \in E[\eta , G(\eta )]$ denote the modified Euler factor at *p* of [9, §2] (see Sect. 2.7). Let $L^{(p)}(\Pi \times \eta , s)$ be the *L*-function without its Euler factor at *p*. Then we have the following conjecture, which is (a generalisation of) the conjectures of Coates–Perrin-Riou and Panchishkin:

##### Conjecture 1.2

(*p*-adic *L*-functions Conjecture for $\textrm{GL}_{2m+1}$)

(i) Suppose $\Pi$ is $P_m$-nearly-ordinary. Then there exists a *p*-adic measure $L_p^-(\Pi )$ on $\mathbb {Z}_p^{\times }$ such that for all $(t,\eta ) \in {{\,\textrm{Crit}\,}}_p^-(\Pi )$, we have the interpolation
  1.2 $$\begin{aligned} \int _{\mathbb {Z}_p^{\times }} \eta (x)^{-1} x^t \cdot \textrm{d}L_p^-\left( \Pi \right) (x) = e_\infty \left( \Pi _\infty \times \eta _\infty , t\right) e_p\left( \Pi _p \times \eta _p, t\right) \cdot \frac{L^{(p)}(\Pi \times \eta , t)}{\Omega _\Pi ^-}, \end{aligned}$$
  and $L_p^-(\Pi )$ vanishes on characters of sign $\ne \omega _\Pi (-1)$.
(ii) Suppose $\Pi$ is $P_{m+1}$-nearly-ordinary. Then there exists a *p*-adic measure $L_p^+(\Pi )$ on $\mathbb {Z}_p^{\times }$ such that for all $(t,\eta ) \in {{\,\textrm{Crit}\,}}_p^+(\Pi )$, we have the interpolation
  $$\begin{aligned} \int _{\mathbb {Z}_p^{\times }} \eta (x)^{-1} x^t \cdot \textrm{d}L_p^+\left( \Pi \right) (x) = e_\infty \left( \Pi _\infty \times \eta _\infty , t\right) e_p\left( \Pi _p \times \eta _p, t\right) \cdot \frac{L^{(p)}(\Pi \times \eta , t)}{\Omega _\Pi ^+} \end{aligned}$$
  and $L_p^+(\Pi )$ vanishes on characters of sign $\ne -\omega _{\Pi }(-1)$.

We call $L_p^{\pm }(\Pi )$ the *left-half* and *right-half* *p*-adic *L*-functions of $\Pi$. Each of these objects, if it exists, is uniquely determined by the interpolation property in the theorem (since it is a measure). If $\Pi$ is nearly ordinary for both $P_m$ and $P_{m+1}$, then we can define a single *p*-adic *L*-function $L_p(\Pi ) = L_p^+(\Pi ) + L_p^-(\Pi )$ interpolating critical values in both halves of the critical strip; but the conditions of near-ordinarity at $P_m$ and $P_{m+1}$ are independent of each other, so it can occur that only one of these objects is well-defined.

In this paper, we prove:

##### Theorem B

Conjecture 1.2 holds in full when $n=2m+1=3$. $\square$

As with Theorem A, it suffices to prove Theorem B for one choice of the sign (and all $\Pi$). This is because $\Pi$ is $P_{m+1}$-nearly-ordinary if $\Pi ^\vee$ is $P_m$-nearly-ordinary, and vice versa; and via the functional equation, we show in §10 that part (i) of the conjecture for $\Pi$ is equivalent to part (ii) of the conjecture for $\Pi ^\vee$. This also gives a *p*-adic functional equation relating $L_p^{\pm }(\Pi )$ and $L_p^{\mp }(\Pi ^\vee )$.

### Mahnkopf’s work on algebraicity

We now describe the starting-point for our constructions, which is Mahnkopf’s demonstration of a weakened form of the Algebraicity Conjecture for $n = 3$. He uses the Rankin–Selberg integral for $\textrm{GL}_3 \times \textrm{GL}_2$: taken with $\Pi$ on $\textrm{GL}_3$ and an Eisenstein series on $\textrm{GL}_2$, this integral computes a product of (twisted) *L*-functions for $\Pi$.

In [45] Mahnkopf gave a cohomological interpretation of this integral, as we now sketch. We take $\Pi$ to have weight $\lambda = (a,0,-a)$, and let $V_\lambda$ be the $\textrm{GL}_3$-representation of highest weight $\lambda$. Note in particular now that

1.3 $$\begin{aligned} {{\,\textrm{Crit}\,}}^-_p(\Pi ) = \{(-j,\eta ) : 0 \le j \le a, \ \textrm{cond}(\eta ) \mid p^\infty , \ \eta \omega _{\Pi }(-1) = (-1)^{j}\}. \end{aligned}$$

For $j \ge 0$, let $V_{(0,-j)}^{\textrm{GL}_2}$ be the $\textrm{GL}_2$-representation of highest weight $(x,y) \mapsto y^{-j}$. Let $Y^{\textrm{GL}_3}(\mathcal {U})$ be the locally symmetric space for $\textrm{GL}_3$ (of some neat level $\mathcal {U}$), and let $Y^{\textrm{GL}_2}_1(p^n)$ be the locally symmetric space (of level $\Gamma _1(p^n)$) for $\textrm{GL}_2$. For a Dirichlet character $\eta$ of conductor $p^n$, one has:

- a compactly supported Betti class $\phi _\varphi \in \textrm{H}_{\textrm{c}}^{2}(Y^{\textrm{GL}_3}(\mathcal {U}), V_\lambda ^\vee (\mathbb {C}))$ attached to any $\varphi \in \Pi _{\textrm{f}}$,
- and Harder’s weight *j* Eisenstein class $\textrm{Eis}^{j,\eta } \in \textrm{H}^1\big (Y^{\textrm{GL}_2}_1(p^n),V_{(0,-j)}^{\textrm{GL}_2}(\mathbb {C})\big )$.

We have the following crucial *branching law* (cf. [46, Lem. 3.1]):

1.4 $$\begin{aligned} If \, (-j,\eta ) \in {{\,\textrm{Crit}\,}}_p^-(\Pi ), \, then \, V_{(0,-j)}^{\textrm{GL}_2} \subset V_\lambda \big |_{\textrm{GL}_2} with \,\,\, multiplicity \,\,\, one. \end{aligned}$$

By pushing forward $\textrm{Eis}^{j,\eta }$ to $Y^{\textrm{GL}_3}$, and using (1.4), one can then define a pairing

1.5 $$\begin{aligned} \langle -,-\rangle : \textrm{H}_{\textrm{c}}^{2}(Y^{\textrm{GL}_3}(\mathcal {U}), V_\lambda ^\vee ) \times \textrm{H}^1\left( Y^{\textrm{GL}_2}_1(p^n),V_{(0,-j)}^{\textrm{GL}_2}\right) \rightarrow \mathbb {C}\end{aligned}$$

such that $\langle \phi _\varphi ,\textrm{Eis}^{j,\eta }\rangle$ is a Rankin–Selberg integral; and for appropriate $\varphi$, one of the *L*-values it computes is $L(\Pi \times \eta , -j)$. Mahnkopf and Kasten–Schmidt used this in [29, 45, 46] to prove that for each fixed *j*, Conjecture 1.1 holds up to replacing $e_\infty (\Pi _\infty \times \eta _\infty , -j)$ with some inexplicit, but non-zero, $\widetilde{e}_\infty (\Pi _\infty \times \eta _\infty , -j) \in \mathbb {C}^{\times }$.

The new result we prove here, in order to complete the proof of Theorem A, is that the inexplicit quantity $\widetilde{e}_{\infty }(\Pi _\infty \times \eta _\infty , -j)$ in fact coincides with the modified archimedean factor $e_\infty (\Pi _\infty \times \eta _\infty , -j)$ of Coates–Perrin-Riou. Our proof of this will in fact be bound up with the proof of Theorem B, which we now sketch.

### *p*-adic interpolation

In light of the equivalence between parts (i) and (ii) of the *p*-adic *L*-functions conjecture, it suffices to focus on part (i), and consider $P_1$-nearly-ordinary refinements and twists in ${{\,\textrm{Crit}\,}}^-_p(\Pi )$.

Mahnkopf’s work expresses $L(\Pi \times \eta ,-j)$ as an integral involving the pushforward to $\textrm{GL}_3$ of the Eisenstein class $\textrm{Eis}^{j,\eta }$. Accordingly, for *p*-adic interpolation of *L*-values the aim is clear; we must:

(I) *p*-adically interpolate the pushforward of $\textrm{Eis}^{j,\eta }$ to $Y^{\textrm{GL}_3}$ as $\eta$ (hence *n*) varies;
(II) *p*-adically interpolate the pushforward of $\textrm{Eis}^{j,\eta }$ to $Y^{\textrm{GL}_3}$ as *j* varies.

This strategy was already known to Mahnkopf over 20 years ago, and work towards (I) was the focus of [46] and [18]. However, these works did not ultimately lead to constructions of *p*-adic *L*-functions, since their methods did not give sufficient control on the denominators of Eisenstein classes to construct a uniquely-determined *p*-adic *L*-function (see Sect. 1.6 below).

In this paper, we solve (I) and (II). Our key innovations are:

- the use of Beĭlinson’s *motivic* Eisenstein classes $\textrm{Eis}^j_{\textrm{mot},\Phi _{\textrm{f},n}}$ (see Sect. 5.3), and
- the systematic variation of a *third* parameter: the definition of the pairing (1.5).

The motivic classes are *norm compatible*, so their Betti realisations – the *Betti–Eisenstein classes* $\textrm{Eis}^j_{\Phi _{\textrm{f},n}}$ – form a tower as *n* varies. Kings has shown they interpolate well as *j* varies (see Sect. 5.3.4). Whilst these classes are not integral, crucially for an auxiliary integer *c* one can define ‘*c*-smoothed’ modifications , that *are* integral and which remain norm-compatible (see Sect. 5.3.3).

By varying the pairing, we build a machine that ports these compatibility and integrality properties from $\textrm{GL}_2$ to $\textrm{GL}_3$. To make this more precise, we elaborate on our approach, which is somewhat different to [45] from the outset. Let , and let $\iota : H \hookrightarrow \textrm{GL}_3$ be the map $$(\gamma ,z) \mapsto \left( {\begin{smallmatrix}\gamma &  \\  &  z\end{smallmatrix}}\right) $$. Our construction involves pulling back Eisenstein classes under the natural projection $H \rightarrow \textrm{GL}_2$, applying the branching law (1.4), pushing forward under $\iota : H \rightarrow \textrm{GL}_3$, and then twisting by a certain operator $u\tau ^n \in \textrm{GL}_3(\mathbb {Q}_p)$, where $$u = \left( {\begin{smallmatrix} 1 &  0 &  1 \\  &  1 &  0 \\  &   &  1\end{smallmatrix}}\right) \left( {\begin{smallmatrix} 1 &   &   \\  &   &  1 \\  &  -1 &  \end{smallmatrix}}\right) $$ and $\tau = \textrm{diag}(p,1,1)$. Varying the parameters in this process varies the pairing (1.5). Ultimately, by ‘spreading out’ this twisted pushforward map over a group algebra, we construct a machine that is, at level *n*, a mapNote that since $Y^{\textrm{GL}_3}$ is 5-dimensional, $\textrm{H}^3(Y^{\textrm{GL}_3}(\mathcal {U}),V_\lambda )$ is Poincaré dual to $\textrm{H}_{\textrm{c}}^{2}(Y^{\textrm{GL}_3}(\mathcal {U}),V_\lambda ^\vee )$.

Importantly, $\overline{P}_1(\mathbb {Z}_p) \cdot u \cdot H(\mathbb {Z}_p)$ is open and dense in $\textrm{GL}_3(\mathbb {Z}_p)$, where we recall the parabolic $P_1 \subset \textrm{GL}_3$ from above. This provides a link with the theory of *spherical varieties*, via $\textrm{GL}_3/H$, as explored in [36]. Exploiting this link, in Theorem 6.10 we show that for each fixed $0 \le j \le a$ as in (1.3), the machine preserves norm-compatibility, but not on the nose: rather, for the Hecke operator $U_{p,1}$ at $P_1$, we prove thatNow let $\varphi \in \Pi$ be a suitable $U_{p,1}$-eigenform with an associated class $\phi _\varphi \in \textrm{H}_{\textrm{c}}^{2}(Y^{\textrm{GL}_3}(\mathcal {U}),V_\lambda ^\vee (\mathcal {O}))$, where $\mathcal {O}$ is a finite extension of $\mathbb {Z}_p$. Let $\alpha _{p,1}$ be the $U_{p,1}$-eigenvalue of $\varphi$. Suppose $\varphi$ is $P_1$-nearly-ordinary, i.e. $\alpha _{p,1} \in \mathcal {O}^{\times }$; then for each fixed *j*, pairing $\alpha _{p,1}^{-n}\phi _\varphi$ with  (under Poincaré duality (3.6)) yields an element . In Sect. 7, we show that if $(-j,\eta ) \in {{\,\textrm{Crit}\,}}^-_p(\Pi )$, then integrating this measure against $\eta$ on $\mathbb {Z}_p^{\times }$ interpolates a relevant Rankin–Selberg integral. In Sects. 8 and 9 we show that for appropriate finite test data, this integral computes (a *c*-smoothed multiple of) the right-hand side of (1.2), up to the precise factor at $\infty$. Taking this test data as input to our machine, and renormalising, yields a family of measures $\Xi ^{[j]}$ that do not depend on the smoothing integer *c* (see Sect. 6.3), such that $\Xi ^{[j]}$ interpolates multiples of $L^{(p)}(\Pi \times \eta ,-j)$ as $\eta$ varies. This then solves (I).

It remains to prove (II), the compatibility between the $a+1$ measures $\{\Xi ^{[j]}\}_{j=0}^a$. This involves *p*-adically interpolating the branching law (1.4) as *j* varies. Again we exploit the connection to spherical varieties: our set-up puts us in the framework of [38], which we apply to prove

$$ \int _{\mathbb {Z}_p^{\times }} x^{j}f(x) \cdot \textrm{d}\Xi ^{[0]}(x)= \int _{\mathbb {Z}_p^{\times }} f(x) \cdot \textrm{d}\Xi ^{[j]}(x), $$

i.e. the $\Xi ^{[j]}$ are Tate twists of each other. Thus the measure $\Xi ^{[0]}$ on $\mathbb {Z}_p^{\times }$ satisfies

$$ \int _{\mathbb {Z}_p^{\times }} \eta (x)x^j \cdot d\Xi ^{[0]}(x) = \int _{\mathbb {Z}_p^{\times }} \eta (x) \cdot \Xi ^{[j]}= (*) \cdot L^{(p)}(\Pi \times \eta , -j), $$

where the term $(*)$ is a product of a global period and certain local zeta integrals. Away from infinity, for a good choice of test data, these local zeta factors are explicitly computed in Sect. 8, and shown to compute the correct interpolation factors. We define $L_p^-(\Pi )$ by precomposing $\Xi ^{[0]}$ with the involution $\chi \mapsto \chi ^{-1}$ on characters of $\mathbb {Z}_p^{\times }$.

This leaves the local zeta integral at $\infty$. By definition, for each *j* this Rankin–Selberg integral is the inexplicit factor $\widetilde{e}_\infty (\Pi _\infty \times \eta _\infty , -j)$ from [46], which is non-zero by [29, 58]. In particular, $L_p^-(\Pi )$ satisfies (1.2) up to replacing $e_\infty (\Pi _\infty \times \eta _\infty ,-j)$ with $\widetilde{e}_\infty (\Pi _\infty \times \eta _\infty , -j)$.

### The factor at $\infty$

To complete the proof of Conjecture 1.2(i), it remains to prove $\widetilde{e}_\infty (\Pi _\infty \times \eta _\infty ,-j) = e_\infty (\Pi _\infty \times \eta _\infty ,-j)$, i.e. that the factor at infinity has the expected form.

By construction, the factor $\widetilde{e}_\infty (\Pi _\infty \times \eta _\infty ,-j)$ depends only on *j* and $\Pi _\infty$, and in turn $\Pi _\infty$ depends only on $\lambda$ and $\omega _\Pi$. Whilst we do not evaluate this integral directly, we *do* know that it is non-vanishing (for all $\Pi _\infty$ and *j*) by [29]. To get the correct interpolation factor $e_\infty$, we exploit the fact that Theorem B is already known in full when $\Pi$ is a (twist of a) symmetric square from $\textrm{GL}_2$. In this case, we compute the ratio of our measure $L_p^-(\Pi )$ with the symmetric square *p*-adic *L*-function, and show this ratio is constant. By (II), we thus deduce that the $\widetilde{e}_\infty (\Pi _\infty \times \eta _\infty ,-j)$’s satisfy the expected compatibility as *j* varies. Up to a global renormalisation, we thus deduce $\widetilde{e}_\infty (\Pi _\infty \times \eta _\infty ,-j) = e_\infty (\Pi _\infty \times \eta _\infty ,-j)$ for all $-j$ in the left-half of the critical strip. Combining with Sect. 1.4, we deduce $L_p^-(\Pi )$ satisfies (1.2), completing the proof of Theorems A and B for sign “−”. In Sect. 10 we use the functional equation to show that this implies the sign “+” case as well, thus completing the proof.

### Relation to previous literature

The most notable partial results towards Theorem B came in works of Mahnkopf [46] (for trivial weight) and Geroldinger [18] (cohomological weight), who gave constructions of algebraic *p*-adic distributions satisfying a partial form of the interpolation (1.2). More precisely, for each critical integer *j* for $\Pi$, they constructed a separate algebraic distribution $L_p^j(\Pi )$ (denoted $\mu _{\Pi ,j}$ in *op. cit*.) satisfying a formula similar to (1.2) for that fixed *j* and all sufficiently ramified $\eta$.

However, using their methods, they were not able to sufficiently control the denominators of their distributions, or obtain any kind of compatibility for varying *j*. In particular, their distributions only defined functions on the set of *locally-algebraic* characters of $\mathbb {Z}_p^{\times }$ of degree $\le a$; they could not prove that their distributions had any uniquely-determined extension from this discrete set to the whole weight space of continuous characters of $\mathbb {Z}_p^{\times }$. Thus their methods did not give a *p*-adic *L*-function in any usual sense (notwithstanding the titles of these papers). This is the fundamental problem we overcome in the present work: our methods give uniform control over the denominators of Eisenstein classes, allowing us to construct a uniquely-determined, bounded measure interpolating all critical values.

(Whilst our *p*-adic methods differ totally from those of [18, 46], these references do contain excellent accounts of a number of the automorphic aspects we require, particularly the local zeta integral at infinity.)

In the special case when $\Pi$ is a symmetric square lift of a RACAR of $\textrm{GL}_2(\mathbb {A}_{\mathbb {Q}})$, a different construction of the *p*-adic *L*-function is possible, using Rankin–Selberg convolutions of the $\textrm{GL}_2$ cusp form with half-integer weight theta-series; see e.g. [11, 23, 56]). However, these methods are completely specific to the case of symmetric-square lifts.

### Possible generalisations

Finally, we comment on possible future generalisations of the method developed here. Most immediately, an appropriate modification of our method should give a generalisation to non-critical slope RACARs of $\textrm{GL}_3(\mathbb {A}_{\mathbb {Q}})$, which is being pursued by Dimitrakopoulou–Rockwood. It should also be possible to generalise these results to RACARs of $\textrm{GL}_3(\mathbb {A}_F)$, where *F* is a totally real field, by replacing Beilinson’s motivic Eisenstein classes with the Hilbert–Eisenstein classes of Beilinson–Kings–Levin [5]. Proposition 3.5.5 *op. cit*. yields the Hilbert analogue of Sect. 5.3.4 of the present paper.

In the longer term, we hope to apply similar methods to construct *p*-adic *L*-functions for RACARs of $\textrm{GL}_n$, over $\mathbb {Q}$ or totally real fields. For this, one requires a supply of well-behaved Eisenstein classes for $\textrm{GL}_{n-1}$. The direct generalisation of our method is not obvious: for $n>3$, the group $\textrm{GL}_{n-1}$ does not give rise to a Shimura datum, and it is not clear what the appropriate generalisation of *motivic* Eisenstein classes should be. However, our methods only require the *Betti realisation* of motivic classes, which are purely topological. It is reasonable to ask if there are norm-compatible families of integral Betti–Eisenstein classes on $\textrm{GL}_{n-1}$, acting as a ‘Betti shadow’ of some deeper, but presently mysterious, motivic structure; and with our methods, such classes should give a *p*-adic *L*-function for $\textrm{GL}_n$. In this direction, a direct construction of such Betti–Eisenstein classes for $\textrm{GL}_2$ over totally real fields was given in [49].

## Preliminaries: automorphic representations

### Characters

If $\chi : (\mathbb {Z}/ N\mathbb {Z})^{\times } \rightarrow \mathbb {C}^{\times }$ is a Dirichlet character, there is a unique finite-order Hecke character $\widehat{\chi }: \mathbb {Q}^{\times } \backslash \mathbb {A}^{\times } / \mathbb {R}^{\times }_{> 0} \rightarrow \mathbb {C}^{\times }$ such that $\widehat{\chi }(\varpi _\ell ) = \chi (\ell )$ for all primes $\ell \not \mid N$, where $\varpi _\ell$ is a uniformiser at $\ell$; conversely, every finite-order Hecke character is $\widehat{\chi }$ for a unique primitive Dirichlet character $\chi$.

Note that the restriction of $\widehat{\chi }$ to $\widehat{\mathbb {Z}}^{\times } \subset \mathbb {A}_{\textrm{f}}^{\times }$ is the *inverse* of the composition $\widehat{\mathbb {Z}}^{\times } \twoheadrightarrow (\mathbb {Z}/ N\mathbb {Z})^{\times } \xrightarrow {\ \chi \ } \mathbb {C}^{\times }$. Moreover, if $\ell \mid N$, then $\widehat{\chi }_\ell (\varpi _\ell ) = \chi ^{(\ell )}(\ell )$, where we have written $\chi = \chi _\ell \chi ^{(\ell )}$ a product of characters of $\ell$-power and prime-to-$\ell$ conductor. Given a representation $\Pi$ of $\textrm{GL}_3(\mathbb {A})$, we write $\Pi \times \chi$ for the representation $\Pi \times [\widehat{\chi }\circ \det ]$.

We let $\psi$ be the unique character of the additive group $\mathbb {A}/ \mathbb {Q}$ such that $\psi (x) = \exp (-2\pi i x)$ for $x \in \mathbb {R}$. Note that the restriction of $\psi$ to $\mathbb {Q}_\ell$, for a finite prime $\ell$, maps $1/\ell ^n$ to $\exp (2\pi i / \ell ^n)$ for all $n \in \mathbb {Z}$. Let $\varepsilon (\widehat{\chi }_\ell ,\psi _\ell )$ be the local $\varepsilon$-factor (with respect to the unramified Haar measure $\textrm{d}x$), as defined in [59] for example. Then we havefor $\chi$ primitive of conductor *N*. (We do not include the Archimedean root number here.)

### Algebraic representations

If *J* is a split reductive group, and $\mu$ a $B_J$-dominant weight for some choice of Borel $B_J \subset J$, then we write $V_\mu ^J$ for the unique irreducible algebraic representation of *J* of highest weight $\mu$. When $J = \textrm{GL}_3$, we will drop the *J* and just write $V_\mu$.

Let *T* denote the diagonal torus of $\textrm{GL}_3$ (identified with $(\mathbb {G}_m)^3$ in the obvious fashion), and $B = T\ltimes N$ the upper-triangular Borel subgroup. The *B*-dominant weights for $\textrm{GL}_3$ are of the form $\lambda = (a,b,c) \in \mathbb {Z}^3$, with $a \ge b \ge c$. If *E* is any $\mathbb {Q}$-algebra, then we can realise $V_\lambda (E)$ as a space of polynomial functions on $\textrm{GL}_3$, via

$$\begin{aligned} V_\lambda (E) = \{ f : \textrm{GL}_3(E)&\rightarrow E \ : \ f\text { algebraic, }\\&f(n^-tg) = \lambda (t)f(g) \ \forall n^- \in N^-(E), t \in T(E)\}, \end{aligned}$$

where $N^-(E)$ is the unipotent radical of the opposite Borel. We get a natural left-action of $\gamma \in \textrm{GL}_3(E)$ on $V_\lambda (E)$ by $(\gamma \cdot f)(g) = f(g\gamma )$. Let $V_\lambda ^\vee (E)$ denote the *E*-linear dual, with the dual left action .

A weight $\lambda = (a,b,c)$ is *pure* if $a + c = 2b$. These are precisely the weights such that $V_{\lambda }^\vee$ is isomorphic to a twist of $V_{\lambda }$.

### Automorphic representations for $\textrm{GL}_3$

We recall some standard facts about automorphic representations of $\textrm{GL}_3$ (for a fuller account, see [47, §3.1], summarising [8, §3]). Let $\Pi$ be a cuspidal automorphic representation of $\textrm{GL}_3(\mathbb {A})$, with central character $\omega _{\Pi }$. We identify $\Pi$ with its realisation in $L^2_0(\textrm{GL}_3(\mathbb {Q})\backslash \textrm{GL}_3(\mathbb {A}))$, considering any $\varphi \in \Pi$ as a function on $\textrm{GL}_3(\mathbb {A})$.

Let $\mathfrak {gl}_3 = \textrm{Lie}(\textrm{GL}_3)$. Recall that the centre of the universal enveloping algebra at $\infty$ acts on $\Pi _\infty$ via a ring homomorphism $Z(U(\mathfrak {gl}_3)_{\mathbb {C}}) \rightarrow \mathbb {C}$ (the *infinitesimal character* of $\Pi _\infty$). We say $\Pi$ is *regular algebraic of weight* $\lambda$ if $\Pi _\infty$ has the same infinitesimal character as the irreducible algebraic representation $V_\lambda$, for some dominant integral weight $\lambda$. (This determines $\lambda$ uniquely.) We use the abbreviation “RACAR” for “regular algebraic cuspidal automorphic representation”.

#### Whittaker models

We denote the standard Whittaker model of $\Pi$ bywhere . As $\psi$ is fixed throughout, we will often drop it from notation. We denote cusp forms in $\Pi$ by $\varphi$, and elements of $\mathcal {W}_\psi (\Pi )$ by *W*.

#### Cohomological automorphic representations

Let $K_{J,\infty }$ be a maximal compact subgroup of $\textrm{GL}_3(\mathbb {R})$, $Z_{\textrm{GL}_3,\infty } = Z_{\textrm{GL}_3}(\mathbb {R})$, and $(-)^\circ$ denote the identity component. Write $K_{3,\infty }^\circ =$ $K_{\textrm{GL}_3,\infty }^\circ Z_{\textrm{GL}_3,\infty }^\circ$ for shorthand. We say $\Pi$ is *cohomological* with coefficients in an algebraic representation *W* if

2.1 $$\begin{aligned} \textrm{H}^\bullet \big (\mathfrak {gl}_3,K_{3,\infty }^\circ ; \Pi _\infty \otimes W(\mathbb {C}) \big ) \ne 0. \end{aligned}$$

##### Proposition 2.1

[8, Lem. 4.9]. If $\Pi$ is a RACAR of weight $\lambda$, then it is cohomological with coefficients in $W = V_{\lambda }^\vee$ (and this is the unique irreducible representation for which $\Pi$ is cohomological). Moreover, $\lambda$ is necessarily pure (as in Sect. 2.2).$\square$

This cohomology is then supported in degrees 2 and 3 [8, Lem. 3.14], and in each of these degrees (2.1) is necessarily one-dimensional [47, (3.2),(3.4)]. We will consider throughout only the lowest degree $i= 2$; exactly as in [46, §3.1] (where it is denoted $\omega _\infty$) we choose a generator

2.2 $$\begin{aligned} \zeta _\infty \in \textrm{H}^2\big (\mathfrak {gl}_3,K_{3,\infty }^\circ ; \Pi _\infty \otimes V_{\lambda }^{\vee }(\mathbb {C}) \big ). \end{aligned}$$

##### Convention

Let $\Pi$ be a RACAR of weight $\lambda$. As in [46, §1] (noting Mahnkopf’s $l_0$ is our $2a+2$), without loss of generality we may (and always will) normalise so that $b = 0$, so (by purity) $\lambda = (a,0,-a)$ for some $a \ge 0$. In this case, we see that

2.3 $$\begin{aligned} \Pi _\infty \cong \textrm{Ind}_{P_2(\mathbb {R})}^{\textrm{GL}_3(\mathbb {R})} (D_{2a+3}, \textrm{id}) \quad \text {or}\quad \textrm{Ind}_{P_2(\mathbb {R})}^{\textrm{GL}_3(\mathbb {R})} (D_{2a+3}, \textrm{sgn}), \end{aligned}$$

where $P_2$ is the parabolic with Levi $\textrm{GL}_2 \times \textrm{GL}_1$, $D_{2a+3}$ is the discrete series representation of $\textrm{GL}_2(\mathbb {R})$ of lowest weight $2a + 3$, and $\textrm{sgn}$ is the sign character. In particular, this implies that its central character $\widehat{\omega }_{\Pi }$ has finite order (i.e. it is the adelic character associated to a Dirichlet character $\omega _\Pi$), and hence $\Pi$ is unitary.

##### Remark 2.2

If $\omega _{\Pi }$ is *odd*, then $\Pi _\infty \cong \textrm{Ind}_{P_2(\mathbb {R})}^{\textrm{GL}_3(\mathbb {R})} (D_{2a+3}, \textrm{id})$, and $\Pi _\infty$ is the twist of this by $\textrm{sgn}$ when $\omega _{\Pi }$ is even. In [46], only the case of $\omega _{\Pi }$ odd is considered, but we allow both signs here.

#### Self-duality

We say $\Pi$ is *self-dual* if $\Pi ^\vee \cong \Pi$, and more generally *essentially self-dual* if $\Pi$ is isomorphic to a twist of $\Pi ^\vee$. A theorem of Ramakrishnan [54] shows that if $\Pi$ is an essentially self-dual RACAR of $\textrm{GL}(3)$, then there exists a non-CM-type cuspidal modular newform *f* of weight $a + 2$, and a character $\nu$, such that $\Pi = \operatorname {Sym}^2(f) \otimes \nu$.

##### Remark 2.3

Ash and Pollack have conjectured [2] that all level 1 RACARs of $\textrm{GL}_3$ are self-dual, and arise as symmetric squares of level 1 cuspidal eigenforms for $\textrm{GL}_2$. Examples which are not essentially self-dual do exist in higher levels; see the tables of [15] for examples.

### The *L*-function of $\Pi$

We let $L(\Pi , s) = \prod _{\ell < \infty } L(\Pi _\ell , s)$ denote the standard *L*-function of $\Pi$ (*without* its Archimedean $\Gamma$-factors). We use the analytic normalisations here, so the Euler product defining $L(\Pi , s)$ converges for $\Re (s) > 1$.

Since $\Pi$ is cohomological, it is *C*-algebraic in the sense of [7], i.e. there exists a number field *E* such that $\Pi _{\textrm{f}}$ is definable as an *E*-linear representation. Since half the sum of the positive roots is in the weight lattice for $\textrm{GL}_3$, it is also *L*-algebraic: that is, if *E* is any number field over which $\Pi _{\textrm{f}}$ is definable, then for primes $\ell$ such that $\Pi _\ell$ is unramified, we have

$$ L(\Pi _\ell , s) = P_\ell \left( \ell ^{-s}\right) ^{-1}, \qquad P_\ell (X) = (1 - \alpha _\ell X)(1 - \beta _\ell X)(1 - \gamma _\ell X) \in E[X], $$

where $\alpha _\ell , \beta _\ell , \gamma _\ell$ are units outside $\ell$, and have valuation $\ge -1-a$ at $\ell$. If $\ell$ is a ramified prime, then we still have $L(\Pi _\ell , s) = P_\ell (\ell ^{-s})$ for some polynomial $P_\ell \in 1 + X E[X]$, but of degree $< 3$.

As $\Pi _\infty$ is given by $(2a+2)\oplus (\pm ,0)$ in the notation of [34, §3], at infinity we have

$$ L_\infty (\Pi _\infty \times \eta _\infty , s) = \Gamma _\mathbb {R}(s + 1 - \kappa ) \Gamma _{\mathbb {C}}(s + a + 1), \qquad \kappa = {\left\{ \begin{array}{ll}0 &  \omega _\Pi \eta \text { is even}\\ 1 &  \omega _\Pi \eta \text { is odd,}\end{array}\right. } $$

where $$\Gamma _{\mathbb {R}}(s) = (2\pi )^{-s/2}\Gamma (s/2)$$, and .

#### Proposition 2.4

Let $\eta$ be a Dirichlet character. Then the critical values of $L(\Pi \times \eta , s)$ are at $s = t$ for integers *t* satisfying either

$$\begin{aligned} \{ -a \le t \le 0 \text { and } (-1)^{t} = \omega _{\Pi }\eta (-1) \} \end{aligned}$$

or

$$\begin{aligned} \{ 1 \le t \le 1+a \text { and } (-1)^{t} = -\omega _{\Pi }\eta (-1) \}. \end{aligned}$$

Note that none of these critical values can be zero (since $L(\Pi \times \eta , s) \ne 0$ for $\operatorname {Re}(s) \ge 1$, giving non-vanishing over $\textrm{Crit}^+$; and non-vanishing over $\textrm{Crit}^-$ follows via the functional equation). In particular, the near-central values $s = 0$ and $s = 1$ of $L(\Pi , s)$ are critical if and only if $\omega _{\Pi }$ is even.

#### Remark 2.5

An important example of $\Pi$ satisfying our conditions is the (normalised) symmetric square lift of a modular form *f* of weight $k = a + 2 \ge 2$ and character $\varepsilon _f$. Then we have $L(\Pi , s) = L(\operatorname {Sym}^2 f, s + a+1)$ and $\omega _{\Pi }(-1) = (-1)^a$, so the above is consistent with the fact that $L(\operatorname {Sym}^2(f), 1)$ and $L(\operatorname {Sym}^2(f) \times \varepsilon _f^{-1}, k-1)$ are always critical values (independent of *a*).

#### Galois representations

By [21], for each prime *p* and embedding $\iota : E \hookrightarrow \overline{\mathbb {Q}}_p$, there is a Galois representation $\rho _{\Pi , \iota }: \operatorname {Gal}(\overline{\mathbb {Q}} / \mathbb {Q}) \rightarrow \textrm{GL}_3(\overline{\mathbb {Q}}_p)$, uniquely determined up to semisimplification, such that for all primes $\ell \ne p$ such that $\Pi _\ell$ is unramified, we have

$$\begin{aligned} \det (1 - X \rho _{\Pi , \iota }(\operatorname {Frob}_{\ell }^{-1})) = P_\ell (X) \end{aligned}$$

Here $\operatorname {Frob}_\ell$ is an arithmetic, and hence $\operatorname {Frob}_{\ell }^{-1}$ a geometric, Frobenius.

Conjecturally $\rho _{\Pi , \iota }$ should be de Rham at *p*, with Hodge–Tate weights $(-1-a, 0, 1+a)$; and if $\Pi _p$ is unramified, then it should be crystalline at *p*, and the eigenvalues of $\varphi$ on $\mathbb {D}_{\textrm{cris}}(\rho _{\Pi , \iota })$ should be $( \alpha _p, \beta _p, \gamma _p)$. More generally, even if $\Pi _p$ is ramified, the Weil–Deligne representation $\mathbb {D}_{\textrm{pst}}(\rho _{\Pi , \iota })$ should be related to $\Pi _p$ via the local Langlands correspondence. This conjecture is “local–global compatibility for $\ell = p$”; it is known if $\Pi$ is essentially self-dual. None of our results will logically rely on this conjecture, or indeed on the existence of $\rho _{\Pi , \iota }$; but it serves as important motivation to explain why the statements are natural ones.

### Rationality of *L*-values

Recall Proposition 2.4, and let

$$\begin{aligned} {{\,\textrm{Crit}\,}}^-(\Pi )&= \{ (-j, \eta ): 0 \le j \le a,\ (-1)^j = \omega _{\Pi }\eta (-1)\} \\ {{\,\textrm{Crit}\,}}^+(\Pi )&= \{ (j+1, \eta ): 0 \le j \le a,\ (-1)^j = \omega _{\Pi }\eta (-1)\} \end{aligned}$$

so $L(\Pi \times \eta , j')$ is critical if and only if $(j', \eta ) \in {{\,\textrm{Crit}\,}}^-(\Pi ) \sqcup {{\,\textrm{Crit}\,}}^+(\Pi )$. For later use we write ${{\,\textrm{Crit}\,}}^{\pm }_p(\Pi ) = \{ (j', \eta ) \in {{\,\textrm{Crit}\,}}^{\pm }(\Pi ): \eta \text { has } p\text { -power conductor }\}$.

If $\Pi$ is a unitary RACAR of $\textrm{GL}_3$, then conjecturally it has an attached motive $M_\Pi$ of weight 0. We note that for $t \in \mathbb {Z}$, the motive $M_\Pi (t)$ should have weight $-2t$, with Hodge decomposition $\textrm{H}_B(M_\Pi (t)(\eta ))\otimes \mathbb {C}= \textrm{H}^{-t-a-1,-t+a+1} \oplus \textrm{H}^{-t,-t} \oplus \textrm{H}^{-t+a+1,-t-a-1}$, each summand 1-dimensional over $\mathbb {C}$. This motivates the following modified Euler factor at infinity:

#### Definition 2.6

If $(-j,\eta ) \in {{\,\textrm{Crit}\,}}^-(\Pi )$, letcorresponding to $t = -j$. If $(j+1,\eta ) \in {{\,\textrm{Crit}\,}}^+(\Pi )$, letcorresponding to $t = j+1$, and where $\epsilon = 0$ if *j* is odd, and $\epsilon = 1$ if *j* is even.

#### Remarks

These factors would be denoted $\mathcal {L}_\infty ^{(i)}(M_\Pi (-j)(\eta ))$ and $\mathcal {L}_\infty ^{(-i)}(M_\Pi (j+1)(\eta ))$ respectively in [9, §1]). In particular, we make opposite choices of parameter (*i* vs. $-i$) in the two critical regions; and where defined, the factors are related by a functional equation

2.4 $$\begin{aligned} e_\infty (\Pi _\infty ^\vee ,1 - t) = \gamma _\infty (\Pi _\infty , t) \cdot e_\infty (\Pi _\infty , t), \end{aligned}$$

(cf. equation (6) *op. cit.*), where $\gamma _\infty (-)$ is the usual local $\gamma$-factor (defined with respect to $\psi$).

These factors $e_\infty (\Pi _\infty \times \eta _\infty , t)$ are the ones appearing in Conjecture 1.1. There are partial results towards this conjecture (see [53] for an overview):

#### Theorem 2.7

(Mahnkopf [45, 46], Kasten–Schmidt [29]) Conjecture 1.1 holds for $n = 3$ up to replacing $e_\infty (\Pi _\infty \times \eta _\infty , t)$ with an inexplicit scalar $\tilde{e}_\infty (\Pi _\infty \times \eta _\infty , t) \in \mathbb {C}^{\times }$.

Note that it is not clear if the ratio $\frac{\tilde{e}_\infty (\Pi _\infty \times \eta _\infty , t)}{e_\infty (\Pi _\infty \times \eta _\infty , t)}$ is independent of *t*, so we cannot simply “absorb” it by renormalising the periods (although this is possible for $a = 0$, since then only one *t*-value can occur for each choice of sign ±).

#### Remark 2.8

We shall recall in Sect. 3.3.1 the definition of a cohomological (Whittaker) period $\Theta _\Pi \in \mathbb {C}^{\times }$ associated to $\Pi$ (well defined up to $E^{\times }$). Note $(\omega _\Pi ^{-1},1) \in {{\,\textrm{Crit}\,}}^+(\Pi )$, and $L(\Pi \times \omega _\Pi ^{-1},1) \ne 0$ (since it is far from the centre $s= \tfrac{1}{2}$ of the critical strip); then, precisely, we take $\Omega _\Pi ^-$ to be an algebraic multiple of $\Theta _\Pi /L(\Pi \times \omega _\Pi ^{-1},1)$. Analogously, $\Omega _\Pi ^+$ will be an algebraic multiple of $\Theta _\Pi /L(\Pi \times \omega _\Pi ^{-1}, 0)$.

### Parabolics, refinements and near-ordinarity at *p*

Let *p* be a prime, and fix an embedding $\iota : E \hookrightarrow \overline{\mathbb {Q}}_p$. Consider the two maximal parabolic subgroups

$$ P_1 = \left( {\begin{smallmatrix} \star &  \star &  \star \\ 0 &  \star &  \star \\ 0 &  \star &  \star \end{smallmatrix}}\right) , \qquad P_2 = \left( {\begin{smallmatrix} \star &  \star &  \star \\ \star &  \star &  \star \\ 0 &  0 &  \star \end{smallmatrix}}\right) . $$

For each $i \in \{1, 2\}$, we can consider the normalised Jacquet module $J_{P_i}(\Pi _p)$, which is an admissible smooth representation of $(\textrm{GL}_i \times \textrm{GL}_{3-i})(\mathbb {Q}_p)$.

#### Definition 2.9

For $i = 1, 2$, a $P_i$*-refinement* of $\Pi _p$ is a choice of an irreducible representation $\sigma _p \times \sigma '_p$ of $(\textrm{GL}_i \times \textrm{GL}_{3-i})(\mathbb {Q}_p)$ appearing as a subrepresentation of $J_{P_i}(\Pi _p)$.

Note that $\sigma _p\times \sigma '_p$ is a $P_1$-refinement of $\Pi _p$ if and only if $\sigma _p^{\prime \vee } \times \sigma _p^\vee$ is a $P_2$-refinement of $\Pi _p^\vee$. For a given $\Pi _p$ and *i*, $\sigma _p'$ is determined by $\sigma _p$ and vice versa, so we will often specify only $\sigma _p$. Accordingly, we say the $P_i$-refinement $\sigma _p \times \sigma _p'$ is *unramified* if $\sigma _p$ is an unramified representation (but $\sigma _p'$ may be ramified).

#### Definition 2.10

The *slope* of a $P_i$-refinement $\sigma _p$ is the valuation of $\iota (\omega _{\sigma _p}(p))$, where $\omega _{\sigma _p}$ is the central character of $\sigma _p$ (and we assume, by enlarging *E* if necessary, that $\sigma _p$ is defined over *E*).

One can check that for a RACAR $\Pi$ of weight $(a, 0, -a)$, the slope of any $P_i$-refinement lies in the interval $[-1-a, 1 + a]$ (this follows from the relation to Hecke eigenvalues which we recall in the next section).

#### Definition 2.11

We say $\Pi$ is $P_i$*-nearly-ordinary* if $\Pi _p$ admits a $P_i$-refinement of slope exactly $-1-a$. This refinement is unique if it exists. We say it is $P_i$*-ordinary* if it is $P_i$-nearly-ordinary, and its unique nearly-ordinary $P_i$-refinement is unramified.

#### Remark 2.12

Clearly, if $\Pi _p$ is itself unramified, then any $P_i$-refinement of it must be unramified. In particular, ‘nearly-ordinary’ and ‘ordinary’ are equivalent if $\Pi _p$ is unramified.

Note, however, that ramified $\Pi _p$ can still admit unramified refinements.

#### Example 2.13

We briefly recall the classification of generic representations of $\textrm{GL}_3$, and explain the conditions under which these are (nearly) ordinary. For simplicity, if $\chi$ is a character of $\mathbb {Q}_p^{\times }$ we write $v(\chi ) = v_p(\iota (\chi (p))$.

- If $\Pi _p$ is supercuspidal, it admits no $P_1$-refinements or $P_2$-refinements, and hence is never nearly-ordinary for any parabolic.
- If $\Pi _p = \operatorname {St}_3 \otimes \lambda$ is a twist of the $\textrm{GL}_3$ Steinberg representation by a character $\lambda$ (necessarily of finite order), then it has a unique $P_1$-refinement and a unique $P_2$-refinement, both of which have slope $-1$. Thus $\Pi _p$ is nearly-ordinary for both parabolics if $a = 0$ (and ordinary if $\lambda$ is unramified), but for $a > 0$ it is not nearly-ordinary for either $P_1$ or $P_2$.
- If $\Pi _p$ is parabolically induced from a representation of $\textrm{GL}_1 \times \textrm{GL}_2$ of the form $\theta \times \pi$, where $\theta$ is a character and $\pi$ is supercuspidal, then its unique $P_1$-refinement is $\theta$, and its unique $P_2$-refinement is $\pi$. So it is $P_1$-nearly-ordinary if and only if $v(\theta ) = -1-a$, whereas it is $P_2$-nearly-ordinary if and only if $v(\theta ) = 1+a$. It is $P_1$-ordinary if it is $P_1$-nearly-ordinary and $\theta$ is unramified, while it is never $P_2$-ordinary.
- If $\Pi _p$ is (irreducibly) induced from $\theta \times (\operatorname {St}_2 \otimes \lambda )$, where $\operatorname {St}_2$ is the $\textrm{GL}_2$ Steinberg representation (and hence $\lambda ^2 \theta = \hat{\omega }_{\Pi , p}$), then it has two $P_1$-refinements, namely $\theta$ and $\lambda |\cdot |^{1/2}$; note that $v(\theta ) \le 1 + a$ implies $v(\lambda |\cdot |^{1/2}) \ge -1-\tfrac{a}{2}$. Thus $\Pi _p$ is $P_1$-nearly-ordinary in either of two (mutually exclusive) cases: if $v(\theta ) = -1-a$ and *a* is arbitrary; or if $a = 0$ and $v(\theta ) = 1$. It is $P_1$-ordinary if $\theta$ is unramified in the former case, and if $\lambda$ is unramified in the latter. (There is a similar criterion for $P_2$-ordinarity, which we leave to the reader.)
- If $\Pi _p$ is an irreducible principal series representation, induced from a character $\chi _1 \times \chi _2 \times \chi _3$ of the diagonal torus, then the possible $P_1$-refinements are exactly the $\chi _i$, and the $P_2$-refinements are the pairs $\{ \{\chi _1, \chi _2\}, \{\chi _2, \chi _3\}, \{\chi _3, \chi _1\}\}$. We can assume without loss of generality that $v(\chi _1) \le v(\chi _2) \le v(\chi _3)$; then $\Pi _p$ is $P_1$-nearly-ordinary if $v(\chi _1) = -1-a$, and $P_1$-ordinary if in addition $\chi _1$ is unramified; it is $P_2$-nearly-ordinary if $v(\chi _1\chi _2) = -1-a$, and $P_2$-ordinary if in addition $\chi _1$ and $\chi _2$ are unramified.

#### Ordinarity and Galois representations

If $\sigma _p$ is a $P_i$-refinement of $\Pi _p$, then $\Pi _p$ is the unique generic constituent of $\operatorname {Ind}_{P_i}^G( \sigma _p \times \sigma '_p)$ for some $\sigma '_p$. Via compatibility of the local Langlands correspondence with parabolic induction, the Langlands parameter $\phi _{\Pi _p}$ has the form

$$\begin{aligned} \begin{pmatrix}\phi _{\sigma _p} & \quad {\star }\\ 0 & \quad \phi _{\sigma '_p} \end{pmatrix}. \end{aligned}$$

Assuming that the Galois representation $\rho _{\Pi , \iota }$ satisfies local–global compatibility at *p*, this gives an *i*–dimensional $(\varphi , N, G_{\mathbb {Q}_p})$-stable subspace of $\mathbb {D}_{\textrm{pst}}(\rho _{\Pi , \iota }|_{G_{\mathbb {Q}_p}})$ isomorphic to $\phi _{\sigma _p}$.

In general this does not arise from a subrepresentation of the Galois representation, since it may not be weakly admissible. However, the following is a straightforward check:

##### Proposition 2.14

Assuming the local–global compatibility conjecture, $\Pi _p$ is $P_i$-nearly-ordinary if and only if $\rho _{\Pi , \iota }|_{G_{\mathbb {Q}_p}}$ preserves an *i*-dimensional subrepresentation accounting for the *i* largest Hodge–Tate weights. Moreover, $\mathbb {D}_{\textrm{pst}}$ of this subrepresentation is the Langlands parameter of the unique nearly-ordinary $P_i$-refinement.$\square$

If the $P_r$-refinement $\sigma _p$ is unramified, then the Satake parameters of $\sigma _p$ are among the reciprocal roots of the Hecke polynomial $P_p(X)$. Thus the existence of an ordinary (rather than nearly-ordinary) $P_r$-refinement is equivalent to the Newton and Hodge polygons coinciding at *r*, which is the “Panchishkin condition” considered in [50, Definition 5.5].

Thus $P_1$-ordinarity corresponds precisely to the *Panchishkin condition* formulated in [50] for the existence of a *p*-adic *L*-function interpolating the *L*-values $L(\Pi \times \eta , -j)$, for $(-j, \eta ) \in {{\,\textrm{Crit}\,}}^-_p(\Pi )$. Similarly, $P_2$-ordinarity corresponds precisely to the Panchishkin condition for interpolation over ${{\,\textrm{Crit}\,}}^+_p(\Pi )$.

### The Coates–Perrin-Riou factor at *p*

Let $t \in \mathbb {Z}$ be such that $(t, \textrm{id}) \in {{\,\textrm{Crit}\,}}(\Pi )$, and let $i = 1$ for ${{\,\textrm{Crit}\,}}^-$ and $i = 2$ for ${{\,\textrm{Crit}\,}}^+$. Let $\sigma _p$ be a $P_i$-refinement of $\Pi _p$.

#### Remark 2.15

In Galois-theoretic terms, *i* is the number of Hodge–Tate weights of the twist $\rho _{\Pi , \iota }(t)$ which are strictly positive, and the condition that $L(\Pi , t)$ be critical implies that this is also the dimension of the $+1$ eigenspace for complex conjugation. So we are choosing a subrepresentation of the Weil–Deligne representation associated to $\rho _{\Pi , \iota }(t)$ whose dimension is equal to the number of positive Hodge–Tate weights.

#### Definition 2.16

Recall $\psi$ is the additive character of $\mathbb {A}/\mathbb {Q}$ such that $\psi (x) = \textrm{exp}(-2\pi i x)$ for $x \in \mathbb {R}$. We briefly denote this by $\psi ^-$, and write $\psi ^+$ for its inverse. For $(t, \textrm{id}) \in {{\,\textrm{Crit}\,}}^\pm (\Pi )$, we set

$$ e_p^\pm (\Pi _p, \sigma _p, t) = \gamma _p(\sigma _p, \psi ^\pm , t)^{-1} :=\frac{L(\sigma _p, t)}{L(\sigma _p^\vee , 1 - t)\varepsilon _p(\sigma _p, \psi _p^\pm , t)}.$$

#### Remark 2.17

Here $\gamma _p(-)$ is the usual local $\gamma$-factor.

Note that in the epsilon factor, we make opposite choices of additive characters for the two critical regions; this ensures the validity of Lemma 2.18. Since the sign ± is determined by *t*, to avoid extraneous notation we henceforth always drop the ± (and $\psi ^\pm$) from notation (except in the proof of Lemma 2.18), and just write $e_p(-)$ and $\gamma _p(-)$.

In this work we shall only consider nearly-ordinary refinements (although we hope that the more general finite-slope case will be studied in future works); since nearly-ordinary refinements are unique if they exist, we shall frequently omit $\sigma _p$ from the notation entirely, and write just $e_p(\Pi _p, t)$.

#### Duality

If $\sigma _p\times \sigma '_p$ is a $P_i$-refinement of $\Pi _p$, then $\sigma _p^{\prime \vee }\times \sigma _p^\vee$ is a $P_{(3-i)}$-refinement of $\Pi _p^\vee$. Our hypotheses are symmetric under replacing $(\Pi , \sigma _p, t, i)$ and with $(\Pi ^\vee , \sigma _p^{\prime \vee }, 1 - t, 3-i)$. We have the following relation between the Coates–Perrin-Riou factors for the two critical regions; compare (2.4) above.

##### Lemma 2.18

We have

2.5 $$\begin{aligned} e_p(\Pi _p^\vee , \sigma _p^{\prime \vee }, 1 - t) = \gamma _p(\Pi _p, t) \cdot e_p(\Pi _p, \sigma _p, t). \end{aligned}$$

##### Proof

By symmetry we may suppose that $(t, \textrm{id}) \in {{\,\textrm{Crit}\,}}_p^-$ and $i = 1$, so the relation to be proved is

$$ \gamma _p((\sigma _p')^\vee , \psi ^+, 1 - t)^{-1} = \gamma _p(\Pi _p, \psi ^-, t) \cdot \gamma _p(\sigma _p, \psi ^-, t)^{-1}. $$

Since $\gamma _p((\sigma _p')^\vee , \psi ^+, 1 - t)^{-1} = \gamma _p(\sigma _p', \psi ^-, t)$, this is equivalent to the “inductivity” property

$$ \gamma _p (\sigma _p, \psi ^-, t) \cdot \gamma _p(\sigma _p', \psi ^-, t) = \gamma _p (\Pi _p, \psi ^-, t). $$

As above, since $\sigma _p \times \sigma _p'$ embeds in $J_{P_i}(V)$, its associated Weil–Deligne representation has a filtration with graded pieces $\sigma _p$ and $\sigma _p'$. The result now follows from the fact that $\gamma$-factors are multiplicative in short exact sequences of Weil–Deligne representations, whether or not the sequence is split (see e.g. [22, Corollary 4.5(ii)]). $\square$

#### Twists

For $\eta$ a Dirichlet character, we have $(t, \eta ) \in {{\,\textrm{Crit}\,}}^{\pm }(\Pi )$ if and only if $(t, \textrm{id}) \in {{\,\textrm{Crit}\,}}^{\pm }(\Pi \times \eta )$. We note that if $\sigma _p$ is a $P_i$-refinement of $\Pi _p$, then $\sigma _p \times \eta _p$ is a $P_i$-refinement of $\Pi _p \times \eta _p$; and this refinement is nearly-ordinary if $\sigma _p$ is.

##### Proposition 2.19

To prove Conjecture 1.2(i) for $n = 3$, it suffices to prove that it holds for all $\Pi$ that are $P_1$-ordinary at *p* (not just $P_1$-nearly-ordinary).

##### Proof

From the discussion above, if $\chi$ is any *p*-power-conductor Dirichlet character, Conjecture 1.2(i) holds for $\Pi$ if and only if it holds for $\Pi \times \chi$ (and the measure $L_p^-(\Pi \times \chi )$ is the twist of $L_p^-(\Pi )$ by $\chi$). Given any $\Pi$ which is $P_1$-nearly-ordinary at *p*, its $P_1$-refinement $\sigma _p$ is a smooth character of $\mathbb {Q}_p^{\times }$, and we can evidently find a Dirichlet character $\chi$ such that $\chi _p \sigma _p$ is unramified; thus $\Pi \times \chi$ is $P_1$-ordinary at *p*. $\square$

##### Remark 2.20

Note this reduction would not work for Conjecture 1.2(ii), or for odd $n \ge 5$.

##### Example 2.21

If $\sigma _p$ is an unramified $P_1$-refinement of $\Pi _p$ with $\sigma _p(p)= \alpha _p \in E^{\times }$, then for all $(-j, \eta ) \in {{\,\textrm{Crit}\,}}_p^-(\Pi )$ we have

### Hecke operators

Let $i \in \{1, 2\}$ and let $N_{P_i}$ and $M_{P_i}$ be the unipotent radical and Levi subgroup of $P_i$. If $K_p \subset \textrm{GL}_3(\mathbb {Z}_p)$ is any open subgroup containing $N_{P_i}(\mathbb {Z}_p)$ and having an Iwahori decomposition with respect to $P_i$, then we have a normalised Hecke operator

2.6 $$\begin{aligned} U_{p, i} = p^a \left[ K_p \ \tau _i \ K_p\right] , \qquad \tau _1 = \left( {\begin{smallmatrix}p \\   &  1\\   & &  1\end{smallmatrix}}\right) , \qquad \tau _2 = \left( {\begin{smallmatrix}p \\   &  p\\   & &  1\end{smallmatrix}}\right) \in \textrm{GL}_3(\mathbb {Q}_p). \end{aligned}$$

on $\Pi _{\textrm{f}}^{K_p}$. The scalar $p^a$ optimally ensures $U_{p,i}$ preserves a $\mathbb {Z}$-lattice in Betti cohomology (see Sect. 3.2), so its eigenvalues are algebraic integers (cf. [3, Rem. 3.13]; our $U_{p,i}$ would be $U_{p,i}^\circ$ *op. cit*.).

From Casselman’s canonical lifting theorem, the subspace of $\Pi _p^{K_p}$ on which the Hecke operator $U_{p, i}$ has finite slope (the sum of its generalised eigenspaces with non-zero eigenvalues) is isomorphic to the invariants of the Jacquet module $J_{P_i}(\Pi )$ under $K_p \cap M_{P_i}$. Moreover, the action of $U_{p, i}$ on this finite-slope subspace is $p^{a+1}$ times the action of $\tau _i \in Z(M_{P_i})$ on $J_{P_i}(\Pi )$ (the $+1$ comes from the modulus character). This result originates in an unpublished note of Casselman; see [14] for an account.

Let us now consider a $P_i$-refinement $\sigma _p \times \sigma '_p$ satisfying the following conditions: $\sigma _p$ is unramified, and all irreducible constituents of $J_{P_i}(\Pi _p) / (\sigma _p \times \sigma '_p)$ have different central characters from $\sigma _p \times \sigma '_p$. Both of these conditions are is automatic if the *p*-refinement is ordinary (since the other constituents, if any, must have strictly larger slope), but is also satisfied in many non-ordinary cases.

#### Remark 2.22

This is a $\textrm{GL}_3$ analogue of the “*p*-regularity” condition often encountered in $\textrm{GL}_2$ theory; if $\Pi _p$ is principal-series and $i = 1$, a $P_1$-refinement is just a choice of one among the three characters from which $\Pi _p$ is induced, and we are assuming that our chosen character is distinct from the other two.

Recalling $\omega _{\sigma _p}$ is the central character of $\sigma _p$ and letting $\alpha _p = \omega _{\sigma _p}(p)$, it follows that the $U_{p, i} = p^{a + 1} \alpha _p$ generalised eigenspace on $\Pi _p^{K_p}$ coincides with the actual eigenspace, and is isomorphic to the $(K_p \cap M_{P_i})$-invariants of $\sigma _p \times \sigma '_p$.

#### Remark 2.23

If $\Pi _p$ is unramified, then its $P_1$-refinements are the unramified characters mapping *p* to the Satake parameters of $\Pi _p$, so our notation $\alpha _p$ is consistent with Sect. 2.4.

### $P_1$-refined new-vectors

We now specialise to the case of an unramified $P_1$-refinement $\sigma _p \times \sigma '_p$. As $\sigma _p$ is an unramified character, the refinement is determined by the number $\alpha _p = \sigma _p(p)$.

For $r \ge 0$, consider the subgroup

2.7 $$\begin{aligned} \mathcal {U}_{1, p}^{(P_1)}(p^r) = \left\{ g: g = 1 \bmod \left( {\begin{smallmatrix} \star &  \star &  \star \\ p &  \star &  \star \\ p^R &  p^r &  p^r\end{smallmatrix}}\right) \right\} \subset \textrm{GL}_3(\mathbb {Z}_p), \end{aligned}$$

where $R = \max (r, 1)$. By construction this subgroup has an Iwahori decomposition with respect to $P_1$; and its intersection the $\textrm{GL}_2$ factor of the Levi of $P_1$ is the level $p^r$ mirabolic subgroup appearing in Casselman’s $\textrm{GL}_2$ local new-vector theory. Hence we have the following:

#### Proposition 2.24

For all sufficiently large *r*, there exists a vector $\varphi _p$ in $\Pi _p$ which is invariant under $\mathcal {U}_{1, p}^{(P_1)}(p^r)$, and on which $U_{p, 1}$ acts via $p^{a+1} \alpha _p$. The minimal such *r*, denoted $r(\Pi _p, \alpha _p)$, is equal to the conductor of the $\textrm{GL}_2(\mathbb {Q}_p)$-representation $\sigma '_p$; and for this *r*, the space of such vectors is 1-dimensional.

This follows from the two results of Casselman discussed above: the canonical lifting theorem, which identifies the $U_{p, 1} = p^{a+1} \alpha _p$ eigenspace with the $(\mathbb {Z}_p^{\times } \times \mathcal {U}_{1, p}^{(\textrm{GL}_2)}(p^r))$-invariants of $\sigma _p \times \sigma '_p$, combined with the local new-vector theorem for $\textrm{GL}_2$ applied to $\sigma '_p$.

We define the $P_1$*-refined local Whittaker newvector* $W^{\alpha _p}$ to be the unique basis of the above 1-dimensional space in the Whittaker model of $\Pi _p$, normalised to be 1 at the identity. By comparing Hecke eigenvalues (using the Hecke-equivariance of Casselman’s lifting), we have the following formula for its values along the torus:

#### Proposition 2.25

We have $$W^{\alpha _p}\left( \left( {\begin{smallmatrix}p^{m+n} \\   &  p^n\\   & &  1\end{smallmatrix}}\right) \right) = 0$$ if $m < 0$ or $n < 0$, and for $m, n \ge 0$ its values are given by

$$ p^{n/2}\left( \tfrac{\alpha _p}{p}\right) ^{(m + n)} W_{\sigma '_p}^{\textrm{new}} \left( \left( {\begin{smallmatrix}p^n &  \\ & 1\end{smallmatrix}}\right) \right) $$

where $W_{\sigma '_p}^{\textrm{new}}$ is the normalised new-vector of the $\textrm{GL}_2(\mathbb {Q}_p)$-representation $\sigma '_p$.$\square$

### Ordinarity for unramified primes

We briefly explain what the above definitions give in the (important!) special case when $\Pi _p$ is unramified. The parabolics $P_i$ correspond to two normalised Hecke operators in the spherical Hecke algebra (cf. (2.6))

2.8 $$\begin{aligned} T_{p, 1} = p^a \left[ \textrm{GL}_3(\mathbb {Z}_p)\ \tau _1\ \textrm{GL}_3(\mathbb {Z}_p)\right] , \qquad T_{p, 2} = p^{a} \left[ \textrm{GL}_3(\mathbb {Z}_p) \ \tau _2 \ \textrm{GL}_3(\mathbb {Z}_p)\right] , \end{aligned}$$

for $\tau _i$ as in (2.6). Let $a_{p, i}(\Pi )$ be the eigenvalue of $T_{p,i}$ on $\Pi _p^{\textrm{GL}_3(\mathbb {Z}_p)}$; as in Sect. 2.8, these are algebraic integers. Recall the Satake parameters $\alpha _p,$ $\beta _p$ and $\gamma _p$ from Sect. 2.4; we have

$$ a_{p, 1}(\Pi ) = p^{a+1}(\alpha _p + \beta _p + \gamma _p),\qquad a_{p, 2}(\Pi ) = p^{a+1}(\alpha _p \beta _p + \beta _p \gamma _p + \gamma _p \alpha _p). $$

Moreover, $a_{p, 2} = \omega _{\Pi }(p) \cdot \overline{a_{p, 1}}$. From these formulae, the following is immediate:

#### Lemma 2.26

$\Pi _p$ is $P_i$-ordinary (with respect to $\iota$) if the eigenvalue $a_{p, i}(\Pi )$ of $T_{p, i}$ acting on $\Pi _p$ is a *p*-adic unit. $\square$

Hence, if we order the Satake parameters so that $v_p(\alpha _p) \le v_p(\beta _p) \le v_p(\gamma _p)$, then $\Pi _p$ is $P_1$-ordinary if and only if $v_p(\alpha _p) = -1-a$ (the smallest possible value), in which case the unique ordinary $P_1$-refinement is $\sigma _p \times \sigma '_p$, where $\sigma _p$ is the unramified character with $\sigma _p(p) = \alpha _p$, and $\sigma '_p$ is the unramified $\textrm{GL}_2$-representation with Satake parameters $\{\beta _p, \gamma _p\}$.

On the other hand, $\Pi _p$ is $P_2$-ordinary if and only if $v_p(\gamma _p) = 1 + a$ (the largest possible), and if so its unique ordinary $P_2$-refinement is $\sigma _p \times \sigma '_p$, where $\sigma _p$ has Satake parameters $\{\alpha _p, \beta _p\}$ and $\sigma '_p$ is the unramified character $p \mapsto \gamma _p$.

#### Remark 2.27

Note that $P_1$-ordinarity and $P_2$-ordinarity are equivalent for essentially self-dual representations (of prime-to-*p* level), but not for general RACARs. Explicit examples which are $P_1$-ordinary but not $P_2$-ordinary can be found in the computations of [15].

In this unramified setting, the quantity $r(\Pi , \alpha _p)$ of Proposition 2.24 is 0, and the corresponding group $\mathcal {U}_{1, p}^{(P_1)}(1)$ is the parahoric subgroup associated to $P_1$; so the $P_1$-refined new vector is simply the unique normalised $(U_{p, 1} = p^{a + 1} \alpha _p$)-eigenvector in the parahoric invariants of $\Pi _p$.

## Symmetric spaces and Betti cohomology

### Symmetric spaces

For any split reductive group *J* over $\mathbb {Q}$, we define a symmetric space for *J* by $\mathcal {H}_J = J(\mathbb {R}) / K_{J, \infty }^{\circ } Z_{J, \infty }^\circ ,$ where $(-)^\circ$ denotes the identity component, $K_{J,\infty }$ is a maximal compact subgroup of $J(\mathbb {R})$, and $Z_\infty = Z_J(\mathbb {R})$. For a neat open compact subgroup $\mathcal {U}\subset J(\mathbb {A}_{\textrm{f}})$, we define

$$ Y^J(\mathcal {U}) = J(\mathbb {Q}) \backslash \left[ J(\mathbb {A}_{\textrm{f}})/\mathcal {U}\times \mathcal {H}_{J} \right] . $$

Note that for $J = \textrm{GL}_n$, the components of $Y^J(\mathcal {U})$ are indexed by the double quotient

$$ Y^{\textrm{GL}_1}(\det \mathcal {U}) = \mathbb {Q}^{\times } \backslash \mathbb {A}^{\times } / \det (\mathcal {U}) \mathbb {R}_{>0}^{\times } \cong \widehat{\mathbb {Z}}^{\times } / \det (\mathcal {U}). $$

Each component of $Y^J(\mathcal {U})$ is the quotient of $\mathcal {H}_J^\circ$ by an arithmetic subgroup of $\textrm{SL}_n(\mathbb {Q})$.

Note $\mathcal {H}_{\textrm{GL}_2}$ is the union of two copies of the complex upper half-plane, and has dimension 2 as a real manifold; while $\mathcal {H}_{\textrm{GL}_3}$ is a 5-dimensional real manifold, again with two components.

#### Remark 3.1

Our conventions match the usual ones for modular curves when $J = \textrm{GL}(2)$. However, note that some papers (such as [20]) use the slightly different quotient $J(\mathbb {Q}) \backslash J(\mathbb {A}) / U K_\infty Z_\infty ^\circ$. For example, if $J = \textrm{GL}(2)$ and $$U = \{ \left( {\begin{smallmatrix}* &  *\\ 0& 1\end{smallmatrix}}\right) \bmod N\}$$, our $Y^J(U)$ is the usual modular curve $Y_1(N)$, while the space considered in [20] is the quotient of $Y_1(N)$ by an anti-holomorphic involution.

### Betti cohomology and Hecke operators

Let $J, \mathcal {U}$ be as above. Given an algebraic representation *V* of *J* (over $\mathbb {Q}$), we have three possible constructions of local systems on $Y^{J}(\mathcal {U})$:

- a local system $\mathscr {V}_{\mathbb {Q}}$ of $\mathbb {Q}$-vector spaces, given by the locally constant sections of the projection
  $$ J(\mathbb {Q}) \backslash \big [\big (J(\mathbb {A}_{\textrm{f}}) \times \mathcal {H}_{J}\big ) \times V(\mathbb {Q})\big ]/\mathcal {U}\longrightarrow Y^J(\mathcal {U}), $$
  with action $\gamma \cdot [(g,z),v] \cdot u = [(\gamma gu, \gamma z), \gamma \cdot v]$. (The functor $V \mapsto \mathscr {V}_{\mathbb {Q}}$ is Pink’s “canonical construction” functor, [51]).
- a local system $\mathscr {V}_{\infty }$ of $\mathbb {R}$-vector spaces, given by the locally constant sections of
  $$ J(\mathbb {Q}) \backslash \big [\big (J(\mathbb {A}_{\textrm{f}}) \times J(\mathbb {R})\big ) \times V(\mathbb {R})\big ]/\mathcal {U}K_{J,\infty }^{\circ } Z_{J,\infty }^{\circ } \rightarrow Y^J(\mathcal {U}),$$
  with action $$\gamma \cdot [(g,z),v] \cdot u k_\infty z_{\infty } = [(\gamma gu, \gamma z), (k_\infty z_{\infty })^{-1} \cdot v]$$.
- a local system of $\mathbb {Q}_p$-vector spaces $\mathscr {V}_p$ for any finite prime *p*, given by the locally constant sections of
  3.1 $$\begin{aligned} J(\mathbb {Q}) \backslash \big [\big (J(\mathbb {A}_{\textrm{f}}) \times \mathcal {H}_{J}\big ) \times V(\mathbb {Q}_p)\big ]/\mathcal {U}\longrightarrow Y^{J}(\mathcal {U}), \end{aligned}$$
  with action $$\gamma [(g,z),v]u = [(\gamma gu, \gamma z), u_p^{-1} \cdot v]$$.

If $\mathcal {U}' \subset \mathcal {U}$, then the formation of $\mathscr {V}_?$ is compatible with the natural projection map $Y^J(\mathcal {U}') \rightarrow Y^J(\mathcal {U})$, and we get natural maps $\textrm{H}^\bullet (Y^J(\mathcal {U}), \mathscr {V}_?) \rightarrow \textrm{H}^\bullet (Y^J(\mathcal {U}'), \mathscr {V}_?)$, where $\textrm{H}^\bullet$ denotes Betti cohomology. We write

$$ \textrm{H}^\bullet (Y^J, \mathscr {V}_{?}) = \varinjlim _{\mathcal {U}} \textrm{H}^\bullet (Y^J(\mathcal {U}), \mathscr {V}_{?}) $$

where $\mathcal {U}$ varies over open compact subgroups of $J(\mathbb {A}_{\textrm{f}})$. This direct limit is naturally a representation of $J(\mathbb {A}_{\textrm{f}})$, and $\textrm{H}^\bullet (Y^J(\mathcal {U}),\mathscr {V}_?) = \textrm{H}^\bullet (Y^J,\mathscr {V}_?)^{\mathcal {U}}.$ We have direct analogues of these statements for compactly-supported cohomology $\textrm{H}^\bullet _c$.

It is standard that there are canonical isomorphisms of local systems

3.2 $$\begin{aligned} \mathscr {V}_{\mathbb {Q}} \otimes \mathbb {Q}_p\cong \mathscr {V}_p, \qquad \mathscr {V}_{\mathbb {Q}} \otimes \mathbb {R}\cong \mathscr {V}_{\infty }. \end{aligned}$$

For instance, the first of these two is given on sections by $[(g,z), v] \mapsto [(g,z), g_p^{-1}\cdot v]$. Details on all of this can be found in [60, §1.2].

The Betti cohomology of $Y^J(\mathcal {U})$ is equipped with a natural action of the Hecke algebra $\mathbb {C}[\mathcal {U}\backslash \textrm{GL}_3(\mathbb {A}_{\textrm{f}}) / \mathcal {U}]$. The isomorphisms on cohomology groups induced by the isomorphisms (3.2) of local systems are all equivariant under the Hecke operators [60, §1.2].

For later use, we describe the $P_1$-Hecke operator at *p* in more detail. Let $\mathcal {U}= \mathcal {U}^{(p)}\mathcal {U}_p \subset \textrm{GL}_3(\mathbb {A}_{\textrm{f}})$ be an open compact subgroup, with $\mathcal {U}_p$ admitting an Iwahori decomposition relative to $P_1$, with notation as in Sect. 2.8. Recall $\tau _i$ from (2.6); for ease of notation, we set . Then we have maps

$$\begin{aligned} Y^{\textrm{GL}_3}(\mathcal {U})\xleftarrow {\ \textrm{pr}_{\mathcal {U}}^{\mathcal {U}\cap \tau \mathcal {U}\tau ^{-1}}\ } Y^{\textrm{GL}_3}(\mathcal {U}\cap \tau \mathcal {U}\tau ^{-1})&\xrightarrow {\ \ \tau \ \ } Y^{\textrm{GL}_3}(\tau ^{-1} \mathcal {U}\tau \cap \mathcal {U})\\&\xrightarrow {\ \textrm{pr}_{\mathcal {U}}^{\tau ^{-1} \mathcal {U}\tau \cap \mathcal {U}}\ } Y^{\textrm{GL}_3}(\mathcal {U}), \end{aligned}$$

where the middle map is induced by right-translation of $\tau$ on $\textrm{GL}_3(\mathbb {A}_{\textrm{f}})$ and the outside maps are the natural projection maps. Passing from left-to-right and right-to-left respectively, we get associated (normalised) Hecke operatorson the cohomology $\textrm{H}^\bullet _*(Y^{\textrm{GL}_3}(\mathcal {U}),\mathscr {V}_?)$, for $* = \varnothing$ or $\textrm{c}$ and $\mathscr {V}_?$ as in Sect. 3.2. As in (2.8), the scalars $p^a$ are for integral normalisation. The operators $U_{p,1}$ and $U_{p,1}'$ are adjoint to each other under Poincaré duality, as we make precise in Sect. 3.4.

We also define *ordinary projectors*  and ; by definition, $U_{p,1}$ is invertible on the image of $e_{\textrm{ord},1}$.

### Automorphic cohomology classes

Let $\Pi$ be a RACAR of $\textrm{GL}_3(\mathbb {A})$. We now realise $\Pi$ in the compactly-supported Betti cohomology of $Y^{\textrm{GL}_3}$. Let $\mathcal {U}\subset \textrm{GL}_3$ be any neat open compact subgroup such that $\Pi _{\textrm{f}}^{\mathcal {U}} \ne 0$, and write $\mathscr {V}_{\lambda ,\mathbb {Q}}^\vee$ for the $\mathbb {Q}$-local system attached to $V_\lambda ^\vee$. Via the cuspidal cohomology, and the natural map $\textrm{H}^\bullet _{\textrm{cusp}} \hookrightarrow \textrm{H}^\bullet _{\textrm{c}}$ of [8, p.123], there is an injectioncompatible with the Hecke action of $\mathbb {C}[\mathcal {U}\backslash \textrm{GL}_3(\mathbb {A}_{\textrm{f}}) / \mathcal {U}]$. Via the choice of $\zeta _\infty$ from (2.2), this yields a map3.3The $\mathbb {Q}$-local systems, and (3.3), are compatible with varying $\mathcal {U}$, giving a $\textrm{GL}_3(\mathbb {A}_{\textrm{f}})$-equivariant map3.4We shall denote the image of this map by $\textrm{H}_{\textrm{c}}^2(\Pi , \mathbb {C})$.

#### Periods and rationality

The representation $\textrm{H}_{\textrm{c}}^2(Y^{\textrm{GL}_3}, \mathscr {V}_{\lambda ,\mathbb {Q}}^\vee (\mathbb {C}))$ has a natural $\mathbb {Q}$-structure given by the cohomology of $\mathscr {V}_{\lambda ,\mathbb {Q}}^\vee (\mathbb {Q})$. Since the complex representation is admissible and contains $\Pi _{\textrm{f}}$ with multiplicity 1, it follows that there is a number field *E*, the *field of definition* of $\Pi$, such that the *E*-linear representation

$$\begin{aligned} \textrm{H}_{\textrm{c}}^2(\Pi , E) = \textrm{H}_{\textrm{c}}^2(\Pi , \mathbb {C}) \cap \textrm{H}_{\textrm{c}}^2\left( Y^{\textrm{GL}_3}, \mathscr {V}_{\lambda ,\mathbb {Q}}^\vee (E)\right) \end{aligned}$$

is non-zero and gives an *E*-structure on $\textrm{H}_{\textrm{c}}^2(\Pi ,\mathbb {C})$. (This is the same field *E* as from Sect. 2.4. By the strong multiplicity one theorem for $\textrm{GL}_3$, we may take *E* to be the field generated by the Hecke eigenvalues of $\Pi$ at the unramified primes, although we shall not need this.)

From the uniqueness of Whittaker models, $\mathcal {W}(\Pi _{\textrm{f}})$ also has a canonical *E*-structure $\mathcal {W}(\Pi _{\textrm{f}}, E)$, given by the functions $W \in \mathcal {W}(\Pi _{\textrm{f}})$ which take values in $E\mathbb {Q}^{\textrm{ab}}$ and satisfy

$$\begin{aligned} W(g)^{\sigma } = W\left( \left( {\begin{smallmatrix}\kappa (\sigma )^2 \\   &  \kappa (\sigma )\\   & &  1\end{smallmatrix}}\right) g\right) \quad \text {for all } \sigma \in \operatorname {Gal}(E \mathbb {Q}^{\textrm{ab}} / E), \end{aligned}$$

where $\kappa : \operatorname {Gal}(\mathbb {Q}^{\textrm{ab}} / \mathbb {Q}) \rightarrow \widehat{\mathbb {Z}}^{\times }$ is the cyclotomic character. By [8, Prop. 3.1], there exists $\Theta _\Pi \in \mathbb {C}^{\times }$ such that

$$ \phi _\Pi \left( \mathcal {W}(\Pi _{\textrm{f}}, E)\right) = \Theta _\Pi \cdot \textrm{H}_{\textrm{c}}^2(\Pi , E). $$

##### Remark 3.2

Having fixed $\zeta _\infty$, the period $\Theta _\Pi$ is uniquely determined modulo $E^{\times }$. We may, however, rescale $\zeta _\infty$ by an arbitrary non-zero complex scalar, which then also rescales $\Theta _\Pi$.

Note that for $\textrm{GL}_n$ with *n* odd, we obtain only a single Whittaker period, since (2.1) is 1-dimensional. This differs from the case of $\textrm{GL}_n$ for *n* even, where (2.1) is 2-dimensional and there are two Whittaker periods (one for each choice of sign at $\infty$).

#### Integral lattices

Let $V_\mu ^J$ be an algebraic representation of a split reductive group *J*, with highest weight vector $v_\mu \in V_\mu ^J$. An *admissible lattice* is a lattice $\mathcal {L}\subset V_\mu$ such that: (1) the map $J_{/\mathbb {Q}} \rightarrow \textrm{GL}(V_\mu )$ extends to a map of $\mathbb {Z}$-group schemes $J_{/\mathbb {Z}} \rightarrow \textrm{GL}(\mathcal {L})$, and (2) the intersection of the highest weight space in $V_\mu$ with $\mathcal {L}$ is $\mathbb {Z}\cdot v_\lambda$.

The set of admissible lattices in $V_\mu ^J$ (for a fixed choice of $v_\mu$) is finite (cf. [40, §4.2]), and there are uniquely-determined maximal and minimal admissible lattices. Since the quotient of these two lattices is a finite abelian group, the base-extensions to $\mathbb {Z}_p$ of these two lattices coincide for all sufficiently large *p* (depending on $\mu$); for instance, if $J = \textrm{SL}_2$, then the minimal and maximal lattices in the *k*-th symmetric power of the standard representation coincide over $\mathbb {Z}_p$ for all $p > k$.

We adopt the convenient (but somewhat misleading) notation that $V_{\mu ,\mathbb {Z}}^{\textrm{GL}_2}$ denotes the *minimal* such lattice in $V_\mu ^{\textrm{GL}_2}$, whilst $V_{\lambda ,\mathbb {Z}}^{\textrm{GL}_3}$ denotes the *maximal* such lattice in $V_\lambda ^{\textrm{GL}_3}$. This ensures that under the branching laws of the next section, $V_{\star ,\mathbb {Z}}^{\textrm{GL}_2}$ is always mapped into $V_{\star ,\mathbb {Z}}^{\textrm{GL}_3}$.

#### Integral structures on cohomology

Now let *p* be a prime, and $v \mid p$ a prime of *E*. Completing *E* at *v*, we may consider

$$ \phi _{\Pi }(\mathcal {W}(\Pi _{\textrm{f}},E)) /\Theta _\Pi \subset \textrm{H}_{\textrm{c}}^2(Y^{\textrm{GL}_3},\mathscr {V}_{\lambda ,\mathbb {Q}}^\vee (E_v)). $$

Note as above that $\mathscr {V}_{\lambda ,\mathbb {Q}}^\vee (E_v) \cong \mathscr {V}_{\lambda ,p}^\vee (E_v)$ on $Y^{\textrm{GL}_3}(\mathcal {U})$. We can define an integral version using the lattice $V_{\lambda ,\mathbb {Z}}$ above (or, more properly, the analogue $V_{\lambda ,\mathbb {Z}}^\vee$, which can be described similarly); the $\mathcal {O}_{E,v}$-points $V_{\lambda ,\mathbb {Z}}(\mathcal {O}_{E,v})$ of this representation carry an action of $\textrm{GL}_3(\mathbb {Z}_p)$, so we get an associated local system $\mathscr {V}_{\lambda ,p}^\vee (\mathcal {O}_{E,v})$ of $\mathbb {Z}_p$-modules (defined exactly as in (3.1)), and where we henceforth drop the $\mathbb {Z}$ for convenience.

Then, for any level $\mathcal {U}$, the space $\textrm{H}_{\textrm{c}}^2\left( Y^{\textrm{GL}_3}(\mathcal {U}), \mathscr {V}_{\lambda ,p}^\vee (\mathcal {O}_{E, v})\right)$ is a finitely-generated $\mathcal {O}_{E, v}$-module; and the quotient of this module by its torsion subgroup is an $\mathcal {O}_{E, v}$-lattice in $\textrm{H}_{\textrm{c}}^2\left( Y^{\textrm{GL}_3}(\mathcal {U}), \mathscr {V}_{\lambda ,p}^\vee (E_v)\right)$.

For any finite subset $\{W_i\} \subset \mathcal {W}(\Pi _{\textrm{f}},E)$, any open compact $\mathcal {U}$ stabilizing all the $W_i$, and any finite extension $L / \mathbb {Q}_p$ into which *E* embeds, we have classes

3.5 $$\begin{aligned} \phi _\Pi (W_i)/\Theta _\Pi \in \textrm{H}_{\textrm{c}}^{2}(Y^{\textrm{GL}_3}(\mathcal {U}),\mathscr {V}_\lambda ^\vee (L)), \end{aligned}$$

depending on the choices of $\zeta _\infty$ (determined up to $\mathbb {C}^{\times }$) and the period $\Theta _\Pi$ (which, given the choice of $\zeta _\infty$, is determined up to $E^{\times }$). Via the embedding $\iota$, we may further rescale $\Theta _\Pi$ to ensure all the $\phi _\Pi (W_i)/\Theta _\Pi$ take values in the lattice $\textrm{H}_{\textrm{c}}^{2}(Y^{\textrm{GL}_3}(\mathcal {U}),\mathscr {V}_\lambda ^\vee (\mathcal {O}_L)) / \mathrm {\{torsion\}}$. Such a normalisation depends on $\{W_i\}$.

##### Remark 3.3

Henceforth we fix a choice of a finite extension $L / \mathbb {Q}_p$ and an embedding $\iota : E \hookrightarrow L$ (factoring through $E_v$ for some prime $v \mid p$), and only use the local systems $\mathscr {V}_{\lambda ,p}^\vee (L)$ or $\mathscr {V}_{\lambda ,p}^\vee (\mathcal {O}_{L})$ constructed from the action of $\textrm{GL}_3(\mathbb {Z}_p)$. To ease notation we will typically drop the subscript *p* and write $\mathscr {V}_\lambda ^\vee$.

### The cup product pairing

Let $\mathcal {U}= \mathcal {U}^{(p)}\mathcal {U}_p\subset \textrm{GL}_3(\mathbb {A}_{\textrm{f}})$ be a neat open compact subgroup and let *R* be a $\mathbb {Z}_p$-algebra. There is a (perfect) Poincaré duality pairing

3.6 $$\begin{aligned} \langle -,-\rangle _{\mathcal {U}}: \frac{\textrm{H}_{\textrm{c}}^{2}\big (Y^{\textrm{GL}_3}(\mathcal {U}), \mathscr {V}_\lambda ^\vee (R)\big )}{\mathrm {(torsion)}} \times \frac{\textrm{H}^3(Y^{\textrm{GL}_3}(\mathcal {U}),\mathscr {V}_\lambda (R))}{\mathrm {(torsion)}} \longrightarrow R \end{aligned}$$

given by composing cup product, the natural pairing $V_\lambda ^\vee (R) \otimes V_\lambda (R) \rightarrow R$, and integration over the smooth (5-dimensional) real manifold $Y^{\textrm{GL}_3}(\mathcal {U})$. If $N_{P_1}(\mathbb {Z}_p) \subset \mathcal {U}_p$ and $\mathcal {U}_p$ has an Iwahori decomposition with respect to $P_1$, then from Sect. 3.2 we have Hecke operators $U_{p,1}$ and $U_{p,1}'$ acting on $\textrm{H}^\bullet _*(Y^{\textrm{GL}_3}(\mathcal {U}),\mathscr {V}_\lambda (R))$, where $* \in \{\varnothing , \textrm{c}\}$. Note that under Poincaré duality, pullback is adjoint to pushforward; thus the operator $U_{p,1}$ (acting on either factor) is adjoint to $U_{p,1}'$ (acting on the other factor) under $\langle -,-\rangle _{\mathcal {U}}$.

## The subgroup *H*

### Definition 4.1

Let $H = \textrm{GL}_2\times \textrm{GL}_1$, and let $\iota : H \hookrightarrow \textrm{GL}_3$ be the embedding

4.1 $$\begin{aligned} \left[ \left( {\begin{smallmatrix}a &  b\\ c &  d\end{smallmatrix}}\right) ,z\right] \longmapsto \left( {\begin{smallmatrix} a &  b &   \\ c &  d &   \\  &   &  z\end{smallmatrix}}\right) . \end{aligned}$$

### Definition 4.2

Let $\nu _1, \nu _2: H \rightarrow \textrm{GL}_1$ be the homomorphisms given by

$$\begin{aligned} \nu _1(\gamma , z) = \tfrac{\det \gamma }{z}\qquad \nu _2(\gamma , z) = z. \end{aligned}$$

Note $(\nu _1, \nu _2)$ gives an isomorphism $H / H^{\textrm{der}} \cong \textrm{GL}_1 \times \textrm{GL}_1$ (this parametrisation of $H / H^{\textrm{der}}$ may seem slightly unnatural, but will give us nicer formulae later), and $\det \circ \mathop {\iota } = \nu _1\nu _2^2$.

### Symmetric spaces

It is important to note that the embedding $\iota : H \rightarrow \textrm{GL}_3$ does not induce a map on symmetric spaces, since the inclusion $Z_{\textrm{GL}_3} \subset \iota (Z_H)$ is strict. To transfer cohomology classes from $Y^H$ to $Y^{\textrm{GL}_3}$, we instead define

$$ \widetilde{\mathcal {H}}_H = H(\mathbb {R}) / \left( K_{H, \infty }^{\circ } \cdot \iota ^{-1}(Z_{G, \infty }^\circ )\right) , $$

which maps naturally to both $\mathcal {H}_H$ and $\mathcal {H}_G$. If $\mathcal {U}\subset H(\mathbb {A}_{\textrm{f}})$ is open compact, let4.2For any open compact $\mathcal {V}\subset \textrm{GL}_3(\mathbb {A}_{\textrm{f}})$, we then have a diagram of maps

4.3 $$\begin{aligned} Y^H(\mathcal {V}\cap H) \leftarrow \widetilde{Y}^H(\mathcal {V}\cap H) \xrightarrow {\ \iota \ } Y^G(\mathcal {V}), \end{aligned}$$

where the right-hand map is induced by $\iota$. Pulling back under the leftward arrow and pushing forward under the rightward arrow gives a map from the cohomology $Y^H$ to that of $Y^{\textrm{GL}_3}$.

If $\mathcal {V}$ is small enough, the left arrow is a fibre bundle with fibres isomorphic to $Z_{H, \infty }^\circ / \iota ^{-1}(Z_{G, \infty }^\circ ) \cong \mathbb {R}$. The spaces $Y^H$ (resp. $\widetilde{Y}^H$) have dimension 2 (resp. 3) as real manifolds.

We get local systems on the modified spaces $\widetilde{Y}^H(\mathcal {U})$ from (4.2), defined identically to Sect. 3.2.

### Branching laws

Note $V^H_{(r, s; t)} = V_{(r,s)}^{\textrm{GL}_2} \otimes V_{(t)}^{\textrm{GL}_1}$ is the *H*-representation of highest weight $$(\left( {\begin{smallmatrix}x &  \\ & y\end{smallmatrix}}\right) , z) \mapsto x^r \cdot y^s \cdot z^t$$. We let $V_{(r,s;t),\mathbb {Z}}^H$ denote the *minimal* admissible lattice in $V_{(r,s;t)}^H$. The following is equivalent to the well-known branching law from $\textrm{GL}_3$ to $\textrm{GL}_2$, e.g. [19, §8].

#### Proposition 4.3

Let $\lambda = (a, 0, -a)$. The restriction of $V_\lambda$ to *H* (embedded via $\iota$) is given by

$$ \iota ^*\left( V_\lambda \right) \cong \bigoplus _{0 \le i,j \le a} V^H_{(j, -i; i-j)}. $$

For such $\lambda$, we shall fix choices of non-zero morphisms of *H*-representations over $\mathbb {Q}$

$$\begin{aligned} \operatorname {br}^{[a,j]}: V^H_{(j, 0; -j)} \rightarrow \iota ^*\left( V_{\lambda }\right) \end{aligned}$$

for each *j* as above. Note $V^H_{(j, 0; -j)} = V^H_{(0, -j; 0)} \otimes \Vert \nu _1^j\Vert$, hence our choice yields a pairing

4.4 $$\begin{aligned} \langle -,-\rangle _{a,j} : V_{\lambda }^\vee \times V_{(0,-j)}^{\textrm{GL}_2} \rightarrow \mathbb {G}_a,\hspace{12pt} (\mu ,v) \mapsto \mu \big ( \textrm{br}^{[a,j]}(\Vert \nu _1^j\Vert \otimes [v \otimes 1])\big ). \end{aligned}$$

As in [40, Prop 4.3.5], $\textrm{br}^{[a,j]}$ maps the (minimal) admissible lattice $V^H_{(j,0;-j),\mathbb {Z}}$ into the (maximal) admissible lattice $V_{\lambda ,\mathbb {Z}}$. Thus the pairing $\langle -,-\rangle _{a,j}$ also makes sense integrally.

## Eisenstein series and classes for $\textrm{GL}_2$

We recall the theory of Eisenstein series and classes attached to adelic Schwartz functions. In particular, we recall the motivic Eisenstein classes of Beĭlinson (see e.g. [40, §7]) and describe their Betti realisations via adelic Eisenstein series. In this section all symmetric spaces will be for $\textrm{GL}_2$, so we write simply $Y(\mathcal {U})$ (resp. *Y*) for $Y^{\textrm{GL}_2}(\mathcal {U})$ (resp. $Y^{\textrm{GL}_2}$).

If $j\ge 0$, recall $V_{(0,-j)}^{\textrm{GL}_2}$ denotes the $\textrm{GL}_2$-representation of highest weight $(0, -j)$. Similarly, in this section only we will drop the superscript and denote this simply $V_{(0,-j)}$.

### Schwartz functions

For a field $K/\mathbb {Q}$, write $\mathcal {S}(\mathbb {A}_{\textrm{f}}^2,K)$ for the Schwartz space of locally-constant, compactly-supported functions on $\mathbb {A}_{\textrm{f}}^2$ with values in *K*, and $\mathcal {S}_0(\mathbb {A}_{\textrm{f}}^2, K)$ for the subspace of $\Phi$ with $\Phi (0, 0) = 0$. We also let $\mathcal {S}(\mathbb {R}^2,K)$ be the usual space of Schwartz functions on $\mathbb {R}^2$, write $\mathcal {S}_0(\mathbb {R}^2,K)$ for the subspace with $\Phi (0,0) = 0$, and let $\mathcal {S}(\mathbb {A}^2,K) = \mathcal {S}(\mathbb {A}_{\textrm{f}}^2,K) \times \mathcal {S}(\mathbb {R}^2,K)$ (and similarly for $\mathcal {S}_0(\mathbb {A}^2,K)$). We will make specific choices at infinity, depending on an integer $j\ge 0$, and use the notation $\mathcal {S}_{(0)}(\mathbb {A}_{\textrm{f}}^2, K)$ to mean “$\mathcal {S}_{0}(\mathbb {A}_{\textrm{f}}^2, K)$ if $j = 0$ or $\mathcal {S}(\mathbb {A}_{\textrm{f}}^2, K)$ otherwise”.

Let $\chi$ be a Dirichlet character, corresponding to a finite-order Hecke character $\widehat{\chi }$. If ${\Phi _{\textrm{f}}}\in \mathcal {S}(\mathbb {A}_{\textrm{f}}^2,K)$, let $R_\chi ({\Phi _{\textrm{f}}}) \in \mathcal {S}(\mathbb {A}_{\textrm{f}}^2,K(\chi ))$ be its projection to the $\widehat{\chi }^{-1}$-isotypical component, given by5.1We emphasise that this is a projection operator, *not* a twisting operator: if $\chi _1,\chi _2$ are distinct primitive Dirichlet characters, then $R_{\chi _1} R_{\chi _2} ({\Phi _{\textrm{f}}}) = 0$.

### Eisenstein series

We now review some standard definitions of Eisenstein series from Schwartz functions, both adelically and classically, and relate these definitions.

#### Adelic Eisenstein series

Let $\Phi = \Phi _\infty \cdot {\Phi _{\textrm{f}}}\in \mathcal {S}(\mathbb {A}^2,\mathbb {C})$ and $\widehat{\chi }: \mathbb {Q}^{\times }\backslash \mathbb {A}^{\times } \rightarrow \mathbb {C}^{\times }$ be a Hecke character. For $g \in \textrm{GL}_2(\mathbb {A})$ and $\Re (s) > 0$ we define a *Godement–Siegel section*5.2This admits meromorphic continuation to $\mathbb {C}$ and gives an element of the family of $\textrm{GL}_2$-principal series representations $I(\Vert \cdot \Vert ^{s-1/2}, \widehat{\chi }^{-1}\Vert \cdot \Vert ^{-s+1/2})$, that is, we have

$$f_\Phi \left( \left( {\begin{smallmatrix}a &  b\\ &  d\end{smallmatrix}}\right) g; \widehat{\chi },s\right) = \widehat{\chi }^{-1}(d)\Vert a/d\Vert ^{s}f_\Phi (g;\widehat{\chi }, s).$$

It thus defines an element of $\mathcal {C}^\infty (B_2(\mathbb {Q})\backslash \textrm{GL}_2(\mathbb {A}),\mathbb {C})$, for $B_2 \subset \textrm{GL}_2$ the upper-triangular Borel. Define (cf. [25, §19])5.3which converges absolutely and locally uniformly on some right half-plane (for $\Re (s) > 1$ if $\widehat{\chi }$ is unitary), defining a function on the quotient $\textrm{GL}_2(\mathbb {Q}) \backslash \textrm{GL}_2(\mathbb {A})$ that transforms under the centre by $\widehat{\chi }^{-1}$. It has meromorphic continuation in *s*, analytic if $\Phi (0, 0) = \hat{\Phi }(0,0) = 0$ or if $\widehat{\chi }$ is ramified at some finite place.

#### Classical Eisenstein series

We now introduce classical Eisenstein series.

##### Definition 5.1

(i) If ${\Phi _{\textrm{f}}}\in \mathcal {S}_{(0)}(\mathbb {A}_{\textrm{f}}^2,K)$ and $j \ge 0$, define  where $s \in \mathbb {C}$ and $\tau \in \mathcal {H}_{\textrm{GL}_2}$. This is a classical real-analytic weight $j+2$ Eisenstein series.
(ii) We extend this to a function on $\textrm{GL}_2(\mathbb {A}_{\textrm{f}}) \times \mathcal {H}$ by setting
(iii) Finally, for a Dirichlet character $\chi$ we also define

We write $\mathcal {E}^{j+2}_{{\Phi _{\textrm{f}}}}$ for each of these functions, but it will be clear from context which we use.

##### Remarks

The function defined in (i) is denoted $E^{(j+2,{\Phi _{\textrm{f}}})}(\tau ;s)$ in [37, Def. 7.1]. These series always converge absolutely for $\Re (s) \ge 1$ unless $j=0$ and $\chi$ is trivial, and even in this case they converge absolutely if $\widehat{\Phi }(0, 0) = 0$.

One has the functional equation $\mathcal {E}_{{\Phi _{\textrm{f}}}}^{j+2}(\tau ;s) = \mathcal {E}_{\widehat{\Phi }_{\textrm{f}}}^{j+2}(\tau ;1-s)$, where $\widehat{\Phi }_{\textrm{f}}$ is the Fourier transform (normalised as in [37, §8.1]); this explains the compatibility between our conventions and those of [40, §7]. The definition in (ii) ensures the association ${\Phi _{\textrm{f}}}\mapsto \mathcal {E}_{{\Phi _{\textrm{f}}}}^{j+2}$ equivariant under $\textrm{GL}_2(\mathbb {A}_{\textrm{f}})$.

Via the standard procedure (see [61, §I]), we now extend $\mathcal {E}_{{\Phi _{\textrm{f}}}}^{j+2}$ to a function on $g = g_{\textrm{f}}g_\infty \in \textrm{GL}_2(\mathbb {A})$. By Iwasawa decomposition, $g_\infty \in \textrm{GL}_2(\mathbb {R})$ can be written uniquely in the form $$\left( {\begin{smallmatrix}z &  \\  &  z\end{smallmatrix}}\right) \left( {\begin{smallmatrix}y &  x\\ 0 &  1\end{smallmatrix}}\right) r(\theta ),$$ where $z \in \mathbb {R}^{\times }$ and $$r(\theta ) = \left( {\begin{smallmatrix}\cos \theta &  \sin \theta \\ -\sin \theta &  \cos \theta \end{smallmatrix}}\right) \in \textrm{SO}_2(\mathbb {R})$$. Then we define

5.4 $$\begin{aligned} \mathcal {E}_{{\Phi _{\textrm{f}}}}^{j+2}&(-;\chi ,-) : \textrm{GL}_2(\mathbb {A}) \times \mathbb {C}\longrightarrow \mathbb {C},\nonumber \\ (g, s)&\longmapsto \widehat{\chi }_\infty ^{-1}(z) \cdot |\det (g_\infty )|^{-s}\cdot y^{\tfrac{j+2}{2}} \cdot \textrm{exp}[{i(j+2)\theta }] \cdot \mathcal {E}_{{\Phi _{\textrm{f}}}}^{j+2}\left( g_{\textrm{f}}, x+iy; \chi , s\right) . \end{aligned}$$

This yields an automorphic form on $\textrm{GL}_2(\mathbb {A})$: the $y^{\tfrac{j+2}{2}}$ and $e^{i(j+2)\theta }$ are always present in extending from $\mathcal {H}_{\textrm{GL}_2}$ to $\textrm{GL}_2(\mathbb {R})$ [61], the $\widehat{\chi }_\infty ^{-1}(z)$ ensures the central character is correct, and $|\det (g_\infty )|^{-s}$ ensures $\mathcal {E}_{{\Phi _{\textrm{f}}}}^{j+2}$ factors through $\textrm{GL}_2(\mathbb {Q})\backslash \textrm{GL}_2(\mathbb {A})$.

#### Comparison of classical and adelic

At infinity, for $j \ge 0$ we shall henceforth take5.5For this choice, by [37, Prop. 10.1] we have:

##### Proposition 5.2

If $\Phi = \Phi _\infty ^{j+2} \cdot {\Phi _{\textrm{f}}}\in \mathcal {S}(\mathbb {A}^2, \mathbb {C})$, for some $j \ge 0$ and $\Phi _{\textrm{f}} \in \mathcal {S}(\mathbb {A}_{\textrm{f}}^2, \mathbb {C})$, and $\chi$ is a Dirichlet character, then the adelic and classical Eisenstein series are related by

$$ E_{\Phi }\Big (g_{\textrm{f}}\left( {\begin{smallmatrix}y &  x\\ 0& 1\end{smallmatrix}}\right) ; \widehat{\chi }, s\Big ) = y^{\tfrac{j+2}{2}} \Vert \det g_{\textrm{f}}\Vert ^{s} \cdot \mathcal {E}^{j+2}_{\Phi _{\textrm{f}}}\Big (g_{\textrm{f}}, x + iy; \chi , s\Big ) $$

for $$g_{\textrm{f}}\in \textrm{GL}_2(\mathbb {A}_{\textrm{f}})$$ and $$x + iy \in \mathcal {H}_{\textrm{GL}_2}$$.

##### Corollary 5.3

When $\Phi _\infty = \Phi _\infty ^{j+2}$, for $g \in \textrm{GL}_2(\mathbb {A})$ we have

$$ E_\Phi (g; \widehat{\chi }, s) = \Vert \det (g)\Vert ^s \mathcal {E}_{{\Phi _{\textrm{f}}}}^{j+2}(g;\chi , s). $$

##### Proof

We have $E_\Phi (g r(\theta ), \widehat{\chi }, s) = e^{i(j+2)\theta }E_{\Phi }(g,\widehat{\chi }, s)$ (cf. [6, §3,(7.35)]); and $E_{\Phi }$ left-translates as $\widehat{\chi }^{-1}_\infty (z)$ under $$\left( {\begin{smallmatrix}z &  \\  &  z\end{smallmatrix}}\right) \in \textrm{GL}_2(\mathbb {R})$$. The result follows from Proposition 5.2. $\square$

#### The special value $s = -j/2$

We shall be chiefly interested in the special value $s = -j/2$ (cf. Theorem 5.5 below). Classically, this has the particularly nice form

$$ \mathcal {E}^{j+2}_{\Phi _{\textrm{f}}}(\tau ,-\tfrac{j}{2}) = \frac{(j+1)!}{(-2\pi i)^{j+2}}\sum _{(m,n) \in \mathbb {Q}^2 \backslash (0,0)} \frac{\widehat{\Phi }_{\textrm{f}}(m,n)}{(m\tau + n)^{j+2}}, $$

where $\widehat{\Phi }_{\textrm{f}}$ is the Fourier transform of $\Phi _{\textrm{f}}$. This function is denoted $F^{j+2}_{{\Phi _{\textrm{f}}}}$ in [40]. Passing to the adeles, this motivates the definitions

5.6 $$\begin{aligned} \mathcal {E}_{{\Phi _{\textrm{f}}}}^{j+2} : \textrm{GL}_2(\mathbb {A})&\longrightarrow \mathbb {C}, \hspace{12pt} g \longmapsto \mathcal {E}_{{\Phi _{\textrm{f}}}}^{j+2}(g; -\tfrac{j}{2}),\nonumber \\ \mathcal {E}_{{\Phi _{\textrm{f}}}}^{j+2,\chi } : \textrm{GL}_2(\mathbb {A})&\longrightarrow \mathbb {C}, \hspace{12pt} g \longmapsto \mathcal {E}_{{\Phi _{\textrm{f}}}}^{j+2}(g;\chi , -\tfrac{j}{2}). \end{aligned}$$

Via Corollary 5.3, we are led to also consider

5.7 $$\begin{aligned} E_{\Phi }^{j+2,\chi } : \textrm{GL}_2(\mathbb {A})&\longrightarrow \mathbb {C}, \hspace{12pt} g \longmapsto \Vert \det g\Vert ^{j/2} E_\Phi \left( g;\widehat{\chi }, -\tfrac{j}{2}\right) . \end{aligned}$$

Then by Corollary 5.3, we have

5.8 $$\begin{aligned} \mathcal {E}_{{\Phi _{\textrm{f}}}}^{j+2,\chi }(g) = E_{{\Phi _{\textrm{f}}}}^{j+2,\chi }(g). \end{aligned}$$

##### Remark 5.4

The functions $\mathcal {E}_{{\Phi _{\textrm{f}}}}^{j+2,\chi }(g) = E_{{\Phi _{\textrm{f}}}}^{j+2,\chi }(g)$ depend $\textrm{GL}_2(\mathbb {A}_{\textrm{f}})$-equivariantly on $\Phi$ and transform as elements of the global principal series representation5.9Note $I_j(\widehat{\chi })$ is irreducible if $j > 0$. For $j = 0$ it does not even have finite length, since infinitely many of the local factors $I_0(\widehat{\chi }_v)$ are reducible.

### Betti–Eisenstein classes

#### Local systems and realisation maps

*Betti local systems:* For $j \ge 0$, we let $\mathscr {V}_{(0,-j)}= \mathscr {V}_{(0,-j)}^{\textrm{GL}_2}$ denote the local system on the $\textrm{GL}_2$ symmetric space associated to the $\textrm{GL}_2$-representation $V_{(0,-j)}$ of highest weight $(0, -j)$. Note that if $a \in \mathbb {Q}_{> 0}$, $$\left( {\begin{smallmatrix}a &  0\\ 0& a\end{smallmatrix}}\right) $$ acts on $\textrm{H}^1(Y, \mathscr {V}_{(0,-j)})$ as multiplication by $a^{-j}$.

*De Rham local systems:* To compare our (Betti)-Eisenstein classes to the classical Eisenstein classes of Harder, we go through a comparison to de Rham cohomology. The local system $\mathscr {V}_{(0,-j)}(\mathbb {C})$ comprises the flat sections of a vector bundle $\mathscr {V}_{(0,-j),\textrm{dR}}$ with respect to the Gauss–Manin connection (see [51, §1.18]). This is defined over $\mathbb {Q}$, and we have a comparison isomorphism5.10The pullback of $\mathscr {V}_{(0,-j),\textrm{dR}}$ to the upper half-plane is $\textrm{Sym}^j\otimes \det ^{-j}$ of the relative de Rham cohomology of $\mathbb {C}/(\mathbb {Z}+\tau \mathbb {Z})$, so has a canonical section $(2 \pi i \textrm{d}z)^{\otimes j}$, for *z* a co-ordinate on $\mathbb {C}$.

*Motivic local systems:* Our Betti–Eisenstein classes will be the Betti realisation of Beĭlinson’s motivic Eisenstein classes. For any level $\mathcal {U}$, there is a relative Chow motive $\mathscr {V}_{(0,-j),\textrm{mot}}(\mathbb {Q})$ over $Y(\mathcal {U})$ attached to the representation $V_{(0,-j)}$ of $\textrm{GL}_2/\mathbb {Q}$; this gives a coefficient system for motivic cohomology. Moreover, the Chow motive has a $\textrm{GL}_2(\mathbb {A}_{\textrm{f}})$-equivariant structure, so its cohomology has a natural Hecke action.

Then we have realisationsBase-changing to $\mathbb {C}$-coefficients, these are related by

5.11 $$\begin{aligned} C_{\textrm{B,dR}}\circ r_{\textrm{B}} = (2\pi i)^{-j-1} \cdot r_{\textrm{dR}} \end{aligned}$$

(see e.g. [33, §2.2] or [41, pf. of Prop. 5.2], noting $T_j(j+1)$ *op. cit.* is $\mathscr {V}_{(0,-j)}(1)$ here).

#### Betti–Eisenstein classes

The main input in our construction is Beĭlinson’s family of motivic Eisenstein classes [4], which we briefly summarise (cf. [40, Thm. 7.2.2]).

##### Theorem 5.5

(Beĭlinson) For any $j \ge 0$, and any level $\mathcal {U}$, there is a canonical $\textrm{GL}_2(\mathbb {A}_{\textrm{f}})$-equivariant map, the *motivic Eisenstein symbol*,

$$ \mathcal {S}_{(0)}(\mathbb {A}_{\textrm{f}}^2, \mathbb {Q})^{\mathcal {U}}\rightarrow \textrm{H}^1_{\textrm{mot}}\left( Y(\mathcal {U}), \mathscr {V}_{(0,-j),\textrm{mot}}(1)\right) ,\qquad {\Phi _{\textrm{f}}}\mapsto {{\,\textrm{Eis}\,}}^j_{\textrm{mot},{\Phi _{\textrm{f}}}}, $$

compatible with Hecke operators and changing $\mathcal {U}$, such that the pullback to the upper half-plane of $r_{\textrm{dR}}(\textrm{Eis}_{\textrm{mot},{\Phi _{\textrm{f}}}}^j)$ is the differential form $-\mathcal {E}^{j+2}_{\Phi _{\textrm{f}}}(\tau ;-\tfrac{j}{2}) \cdot (2\pi i\textrm{d}z)^{\otimes j} \cdot 2\pi i\textrm{d}\tau$ (cf. Sect. 5.3.1).

##### Corollary 5.6

For any $j \ge 0$, and any level $\mathcal {U}$, there is a canonical $\textrm{GL}_2(\mathbb {A}_{\textrm{f}})$-equivariant map, the *Betti–Eisenstein symbol*,compatible with Hecke operators and changing $\mathcal {U}$, such that $C_{\textrm{B,dR}}(\textrm{Eis}_{\Phi _{\textrm{f}}}^j)$ is the differential form $-\mathcal {E}^{j+2}_{\Phi _{\textrm{f}}}(g) \cdot (\textrm{d}z)^{\otimes j} \cdot \textrm{d}\tau$ on $Y(\mathcal {U})$.

##### Proof

By (5.11), the pullback to the upper half-plane of $C_{\textrm{B,dR}}(\textrm{Eis}_{\Phi _{\textrm{f}}}^j)$ is $-\mathcal {E}^{j+2}_{{\Phi _{\textrm{f}}}}(\tau ;-\tfrac{j}{2}) \cdot (\textrm{d}z)^{\otimes j} \cdot \textrm{d}\tau$. The extension of $\mathcal {E}_{{\Phi _{\textrm{f}}}}^{j+2}$ to $\textrm{GL}_2(\mathbb {A}_{\textrm{f}}) \times \mathcal {H}$ in Definition 5.1(ii) is the unique one preserving $\textrm{GL}_2(\mathbb {A}_{\textrm{f}})$-equivariance, and the further extension of this to $\textrm{GL}_2(\mathbb {A})$ in (5.4) is the unique one that is automorphic; it follows that the extension of the differential to *Y*(*U*) has form $-\mathcal {E}^{j+2}_{{\Phi _{\textrm{f}}}}(g;-\tfrac{j}{2})\otimes (\textrm{d}z)^j \otimes \textrm{d}\tau$. But $\mathcal {E}^{j+2}_{{\Phi _{\textrm{f}}}}(g) = \mathcal {E}^{j+2}_{{\Phi _{\textrm{f}}}}(g;-\tfrac{j}{2})$ by definition. $\square$

#### Integrality

In general these Eisenstein symbols do not take values in the integral cohomology. However, we can work around this as follows. Let $c > 1$ be coprime to 6, and let $\mathcal {U}$ be a level of the form $\mathcal {U}^{(c)} \times \prod _{\ell \mid c} \textrm{GL}_2(\mathbb {Z}_\ell )$. We write  for the $\mathbb {Z}$-valued Schwartz functions of the form $\Phi _{\textrm{f}}^{(c)} \times \prod _{\ell \mid c} \operatorname {ch}(\mathbb {Z}_\ell ^2)$, and similarly .

##### Theorem 5.7

There exist homomorphismscompatible with Hecke operators and changing $\mathcal {U}$, such that after base-extending to $\mathbb {Q}$ we havewhere $$\left( {\begin{smallmatrix}c &  0\\ 0& c\end{smallmatrix}}\right) $$ is understood as an element of $\textrm{GL}_2(\mathbb {A}_{\textrm{f}}^{(c)})$.

#### *p*-adic interpolation

Let us now fix a prime *p*, an open compact $\mathcal {U}^{(p)} \subseteq \textrm{GL}_2(\mathbb {A}_{\textrm{f}}^{(p)})$, and a prime-to-*p* Schwartz function $\Phi ^{(p)} \in \mathcal {S}(\mathbb {A}_{\textrm{f}}^{(p)},\mathbb {Z})^{\mathcal {U}^{(p)}}$. We suppose *c* is coprime to *p*, and that $\mathcal {U}$ and $\Phi ^{(p)}$ are unramified at the primes dividing *c*. After tensoring with $\mathbb {Z}_p$, we can take the local systems $\mathscr {V}_{(0,-j)}^{\textrm{GL}_2}$ (and the class ) to have $\mathbb {Z}_p$-coefficients.

For $t \ge 0$, let , a Schwartz function at *p*. The Schwartz functions , for $t \ge 1$, are stable under the group $$\mathcal {U}_1(p^t) = \mathcal {U}^{(p)} \cdot \{ \left( {\begin{smallmatrix}* &  *\\ 0& 1\end{smallmatrix}}\right) \bmod p^t\}$$, and compatible with the trace maps for varying *t*. Extending from $\mathbb {Z}$- to $\mathbb {Z}_p$-coefficients, we obtain an *Eisenstein–Iwasawa class*As explained in [40, §9.1], there are natural “moment” maps

$$ {{\,\textrm{mom}\,}}^j_t: \textrm{H}^1_{\textrm{Iw}}\left( Y(\mathcal {U}_1(p^\infty )), \mathbb {Z}_p\right) \rightarrow \textrm{H}^1\left( Y(\mathcal {U}_1(p^t)), \mathscr {V}_{(0,-j)}({\mathbb {Z}_p})\right) $$

for each $j \ge 0$ and $t \ge 0$; and if $t \ge 1$, we haveThis is true by definition for $j = 0$; that it also holds for $j > 0$ is a deep theorem due to Kings. There is a similar statement for $t = 0$, but we need to replace the group $\mathcal {U}_1(p^t)$ with the $\textrm{GL}_2$ Iwahori subgroup $\mathcal {U}_0(p)$. All of the above structures are compatible with the action of $\textrm{GL}_2(\mathbb {A}_{\textrm{f}}^{(pc)})$.

Note that the Iwasawa cohomology group containing  is a module over the Iwasawa algebra $\Lambda = \mathbb {Z}_p[\![\mathbb {Z}_p^{\times }]\!]$, and the moment map ${{\,\textrm{mom}\,}}^j_t$ factors through the quotient where $(1 + p^t \mathbb {Z}_p)^{\times }$ acts via $x \mapsto x^j$. If $\Phi ^{(p)}$ belongs to the $\widehat{\chi }$-eigenspace for the action of the centre, for some Dirichlet character $\chi$ of prime-to-*p* conductor (taking values in a finite extension $\mathcal {O}$ of $\mathbb {Z}_p$), then the elementis independent of *c*, where $\textbf{j}$ is the universal character $\mathbb {Z}_p^{\times } \hookrightarrow \Lambda ^{\times }$. In particular, if $\chi$ is non-trivial, then we can choose *c* so that the above factor is invertible in $\Lambda \otimes \mathbb {Q}_p$, and hence define $\mathcal{E}\mathcal{I}_{\Phi ^{(p)}}$ as an element of $\textrm{H}^1_{\textrm{Iw}} \otimes _{\mathbb {Z}_p} \mathbb {Q}_p$. (We can even work integrally if $\chi \ne \textbf{1} \hspace{2pt}\left( \textrm{mod}\hspace{2pt}p\right)$).

## Constructing the measure

We fix henceforth a prime *p*, allowing $p = 2$. We now construct a *p*-adic measure interpolating pushforwards (to $\textrm{GL}_3$) of Betti–Eisenstein classes. For this, we use the norm compatibility of these classes to systematically control their denominators after pushforward. Crucially, the measure we construct will be valued in the dual to degree 2 compactly supported cohomology for $\textrm{GL}_3$.

### Notation 6.1

We work with a fixed RACAR $\Pi$ in mind, of weight $\lambda = (a,0,-a)$, with $a \in \mathbb {Z}_{\ge 0}$. Our *p*-adic *L*-function will interpolate the critical *L*-values in the left half ${{\,\textrm{Crit}\,}}_p^-(\Pi )$ of the critical strip. This will correspond to privileging the parabolic $P_1 \subset \textrm{GL}_3$, with associated Hecke operator $U_{p,1}$, and considering $P_1$-refinements as in Sect. 2.6. Let $\sigma _p$ be a $P_1$-refinement of $\Pi$; via Proposition 2.19, replacing $\Pi$ by a character twist if necessary, we may suppose that $\sigma _p$ is unramified. Our local level at *p* will be6.1(see (2.7), for $r(\Pi ,\alpha _p)$ as in Proposition 2.24, recalling $\alpha _p = \sigma _p(p)$).

Let $\Phi ^{(p)} \in \mathcal {S}(\mathbb {A}_{\textrm{f}}^{(p),2},\mathbb {Z})$. We fix a tame level $\mathcal {U}^{(p)} \subset \textrm{GL}_3(\mathbb {A}_{\textrm{f}}^{(p)})$ such that $\Phi ^{(p)}$ is fixed by $\mathcal {U}^{(p)}\cap H$. We also fix an integer $c > 1$ coprime to 6*p* and to the levels of $\mathcal {U}^{(p)}$ and $\Phi ^{(p)}$. Let $\mathcal {U}= \mathcal {U}^{(p)}\mathcal {U}_p$, which – up to shrinking $\mathcal {U}^{(p)}$ – we may assume is neat.

### Definition of the classes

#### Branching laws and Eisenstein classes

Recall $H = \textrm{GL}_2 \times \textrm{GL}_1$. For numerology purposes, our pushforward maps will be from $\widetilde{Y}^H$ to $Y^{\textrm{GL}_3}$, so we now transfer our Eisenstein classes from $Y^{\textrm{GL}_2}$ to $\widetilde{Y}^H$. If $\mathcal {U}^H \subset H(\mathbb {A}_{\textrm{f}})$ is open compact, we have a natural composition

$$ \textrm{pr}_{\textrm{GL}_2}: \widetilde{Y}^H(\mathcal {U}^H) \longrightarrow Y^H(\mathcal {U}^H) \longrightarrow Y^{\textrm{GL}_2}(\mathcal {U}^{H}\cap \textrm{GL}_2), $$

where the first map is from (4.3) and the second map is induced by projection $H \rightarrow \textrm{GL}_2$. Pullback gives a map

$$ \textrm{pr}_{\textrm{GL}_2}^*: \textrm{H}^1\left( Y^{\textrm{GL}_2}(\mathcal {U}^{\textrm{GL}_2}), \mathscr {V}_{(0,-j)}^{\textrm{GL}_2}\right) \longrightarrow \textrm{H}^1\left( \widetilde{Y}^H(\mathcal {U}^H), \mathscr {V}^H_{(0,-j;0)}\right) . $$

For appropriate $\mathcal {U}^H$ we freely identify  with its image under this map.

Let $j \in \mathbb {Z}$ with $0 \le j \le a$. Recall the characters $\nu _1,\nu _2$ on *H* from Definition 4.2. We have an isomorphismof *H*-representations. Passing this twist outside the cohomology inverts it, so for appropriate $\mathcal {U}^H$ we may thus considerRecall the morphisms $\operatorname {br}^{[a,j]}: V^H_{(j, 0; -j)}\rightarrow \iota ^*(V_\lambda )$ defined in Sect. 4.2. We may (and do) suppose that these are integrally defined, and pushing forward we obtain an $H(\mathbb {A}_{\textrm{f}})$-equivariant map

$$ \textrm{br}^{[a,j]}_\star : \textrm{H}^1\big (\widetilde{Y}^H(\mathcal {U}^H), \mathscr {V}_{(j,0;-j)}^H(\mathbb {Z}_p)\big ) \rightarrow \textrm{H}^1\left( \widetilde{Y}^H(\mathcal {U}^{H}), \iota ^*\mathscr {V}_{\lambda }(\mathbb {Z}_p)\right) . $$

#### Towers of level groups

We now pushforward under $\iota$. Let $r = r(\Pi ,\alpha _p)$ from Proposition 2.24.

##### Definition 6.2

For $n \ge 1$, let $\mathcal {U}_n$ denote the group

$$ \mathcal {U}^{(p)} \times \left\{ g \in \textrm{GL}_3(\mathbb {Z}_p): g = 1 \bmod \left( {\begin{smallmatrix} \star &  p^n &  p^{n} \\ \star &  \star &  \star \\ p^{r} &  p^r &  p^r\end{smallmatrix}}\right) \right\} . $$

Write *u* for the element $$\left( {\begin{smallmatrix} 1 &  0 &  1 \\  &  1 &  0 \\  &   &  1\end{smallmatrix}}\right) \left( {\begin{smallmatrix} 1 &   &   \\  &   &  -1 \\  &  1 &  \end{smallmatrix}}\right) \in \textrm{GL}_3(\mathbb {Z}_p)$$.

We shall let $t \ge \textrm{max}(n,r)$ and consider the morphism

$$ \iota _{n, t}: \widetilde{Y}^H(\mathcal {U}_{1}^H(p^t) \cap u \mathcal {U}_n u^{-1}) \xrightarrow {\ \iota \ } Y^{\textrm{GL}_3}(u \mathcal {U}_n u^{-1}) \xrightarrow {\ \ u\ \ } Y^{\textrm{GL}_3}(\mathcal {U}_n), $$

where

$$ \mathcal {U}_{1}^H(p^t) = \left( \mathcal {U}^{(p)} \cap H(\mathbb {A}_{\textrm{f}}^{(p)})\right) \times \{ (\left( {\begin{smallmatrix}\star &  \star \\ 0& 1\end{smallmatrix}}\right) , \star ) \bmod p^t\}. $$

##### Proposition 6.3

If $t \ge \textrm{max}(n,r)$, then $\mathcal {U}_{1}^H(p^t) \cap u \mathcal {U}_n u^{-1}$ consists of all $$(\left( {\begin{smallmatrix}a &  b\\ c& d\end{smallmatrix}}\right) , z) \in \mathcal {U}_{1}^H(p^t)$$ with $b = 0\bmod p^{n}$, $a = z \bmod p^n$. Its index in $\mathcal {U}_1^H(p^t)$ is $p^{2n-1}(p-1)$.

##### Proof

Let $$g = \left( {\begin{smallmatrix} A &  \quad &  B \\ \quad &  C &  \quad \\ D &  \quad &  E\end{smallmatrix}}\right) \quad {F}\quad {G}\quad {H}\quad {I} \in \mathcal {U}_n$$. Then suppose there exists $$\left( \left( {\begin{smallmatrix}a &  b\\ c &  d\end{smallmatrix}}\right) , z\right) \in \mathcal {U}_{1}^H(p^t)$$ with

$$ \left( {\begin{smallmatrix} a &  \quad &  b \\ \quad &  0 &  \quad \\ c &  \quad &  d\end{smallmatrix}}\right) \quad {0}\quad {0}\quad {0}\quad {z} = \iota \Big (\left( {\begin{smallmatrix}a &  \quad \\ b &  \quad \end{smallmatrix}}\right) {c}\quad {d}, z\Big ) = ugu^{-1} = \left( {\begin{smallmatrix} A+D &  -(C+F) &  (B+E) - (A+D) \\ -G &  I &  G-H \\ D &  -F &  -D+E\end{smallmatrix}}\right) . $$

Then $D=F=0$, so $b = -(C+F) = -C \equiv 0 \hspace{2pt}\left( \textrm{mod}\hspace{2pt}p^n\right)$, and $z = -D+E = E$. Moreover $(B+E)-(A+D) = 0$ implies $a= A+D = A \equiv E = z \hspace{2pt}\left( \textrm{mod}\hspace{2pt}p^n\right)$, as required. Since $t \ge r$, the congruence conditions $\bmod p^r$ satisfied by *G*, *H* and *I* in the definition of $\mathcal {U}_n$ impose no further restrictions on $c \equiv 0 \hspace{2pt}\left( \textrm{mod}\hspace{2pt}p^t\right)$ and $d \equiv 1 \hspace{2pt}\left( \textrm{mod}\hspace{2pt}p^t\right)$. The restrictions on *b* and *z* introduce $p^n$ and $p^{n-1}(p-1)$ to the index respectively. $\square$

#### Construction at fixed level *n*

Recall the map $\nu = (\nu _1,\nu _2): H \rightarrow \textrm{GL}_1 \times \textrm{GL}_1$ given by $(\gamma , z) \mapsto (\tfrac{\det \gamma }{z}, z)$. By Proposition 6.3, we have $\nu _1(h_p) = 1\hspace{2pt}\left( \textrm{mod}\hspace{2pt}p^n\right)$ for all $h \in \mathcal {U}_{1}^H(p^t) \cap u \mathcal {U}_n u^{-1}$. Thus $\nu _1$ induces a locally constant map6.2

##### Remark 6.4

Here we have identified $\Delta _n$ with a quotient of $\mathbb {Q}^{\times } \backslash \mathbb {A}^{\times }$, in such a way that a uniformiser at a prime $\ell \ne p$ in $\mathbb {A}_{\textrm{f}}^{\times }$ is mapped to $\ell \bmod p^n$. This is consistent with our convention for Dirichlet characters elsewhere in the text (but its restriction to $\mathbb {Z}_p^{\times } \subset \mathbb {A}^{\times }$ is the inverse of the obvious reduction map).

Composing with the canonical map $\Delta _n \rightarrow \mathbb {Z}_p[\Delta _n]^{\times }$, $\delta \mapsto [\delta ]$, we obtain a locally constant function $[\nu _{1, (n)}]$ on $\widetilde{Y}^H\left( \mathcal {U}_{1}^H(p^t) \cap u \mathcal {U}_n u^{-1}\right)$ with values in the group ring $\mathbb {Z}_p[\Delta _n]$, i.e. an element of $\textrm{H}^0\left( \widetilde{Y}^H\left( \mathcal {U}_{1}^H(p^t) \cap u \mathcal {U}_n u^{-1}\right) , \mathbb {Z}_p\right) \otimes _{\mathbb {Z}_p} \mathbb {Z}_p[\Delta _n]$.

##### Notation 6.5

For technical reasons (see Proposition 6.13), we introduce a second auxiliary character. Let $\eta _2$ be an even Dirichlet character of prime-to-*p* conductor, which (for notational convenience) we suppose to be $\mathbb {Z}_p$-valued. Shrinking $\mathcal {U}^{(p)}$ if necessary, we may suppose $\mathcal {U}_{1}^H(p^t) \cap u \mathcal {U}_n u^{-1} \subseteq \ker (\widehat{\eta }_{2})$, and thus regard $\widehat{\eta }_{2} \circ \nu _2$ as a class in $\textrm{H}^0\left( \widetilde{Y}^H\left( \mathcal {U}_{1}^H(p^t) \cap u \mathcal {U}_n u^{-1}\right) , \mathbb {Z}_p\right)$.

##### Definition 6.6

For any $t \ge \textrm{max}(n,r)$, we defineLet $z_{n}^{[a,j]}(-) \in \textrm{H}^3\left( Y^{\textrm{GL}_3}(\mathcal {U}_n), \mathscr {V}_\lambda (\mathbb {Q}_p)\right) \otimes _{\mathbb {Z}_p} \mathbb {Z}_p[\Delta _n]$ be the analogue defined instead using ${{\,\textrm{Eis}\,}}_{\Phi _{\textrm{f}}^t}^j$ (without the *c* factor).

These classes are independent of *t* for $t \ge \textrm{max}(n,r)$, since the maps $\iota _{n, t}$ for varying *t* are compatible with the natural trace maps. The normalisation term $p^{-an}$ will later ensure compatibility with the normalised Hecke operator $U_{p,1}$ (see (2.6)).

##### Remark 6.7

Philosophically, this process formally ‘spreads out’ the cohomology over (unions of) connected components. More precisely, let $D = \textrm{cond}(\eta _2)$. Then we have a map

$$ \nu _{1,(n)} \times \nu _{2,D}: \widetilde{Y}^H\left( \mathcal {U}_{1}^H(p^t) \cap u \mathcal {U}_n u^{-1}\right) \twoheadrightarrow \Delta _n \times (\mathbb {Z}/D)^{\times }, $$

and

$$ \widetilde{Y}^H\left( \mathcal {U}_{1}^H(p^t) \cap u \mathcal {U}_n u^{-1}\right) = \bigsqcup _{(x,y) \in \Delta _n \times (\mathbb {Z}/D)^{\times }} \widetilde{Y}^H_{x,y}, $$

where  is a union of connected components. Let $i_{x,y}: \widetilde{Y}^H_{x,y} \hookrightarrow \widetilde{Y}^H\left( \mathcal {U}_{1}^H(p^t) \cap u \mathcal {U}_n u^{-1}\right)$ be the inclusion. Then

Our chief interest is in a modified version of these classes.

##### Definition 6.8

Let  from (2.6), and let

Translation by $\tau ^n$ gives a map $Y^G(\mathcal {U}_n) \rightarrow Y^G(\mathcal {V}_n)$. Crucially, $\mathcal {V}_n \subset \mathcal {U}_p$ from (6.1). We use this to define a pushforward map $(\tau ^n)_\star$ on cohomology with coefficients in $\mathscr {V}_\lambda$, scaling by $p^a$ (as in our definition of Hecke operators) in order to obtain a map on the integral lattices $\mathscr {V}_\lambda (\mathbb {Z}_p)$.

##### Definition 6.9

We setSimilarly, define $\xi ^{[a,j]}_n(-)$ (with $\mathbb {Q}_p$ coefficients) to be the analogue using $z_{n}^{[a,j]}(-)$.

The dependence on $c \in \mathbb {Z}$ is given by6.3where [*c*] is the class of *c* in $\Delta _n = (\mathbb {Z}/p^n)^{\times }$, and $\langle c \rangle$ denotes pullback by $\operatorname {diag}(c, c, c)^{-1} \in Z_G(\widehat{\mathbb {Z}}^{(c)})$.

#### Summary

The following diagram summarises the construction of . For notational convenience, we write . 
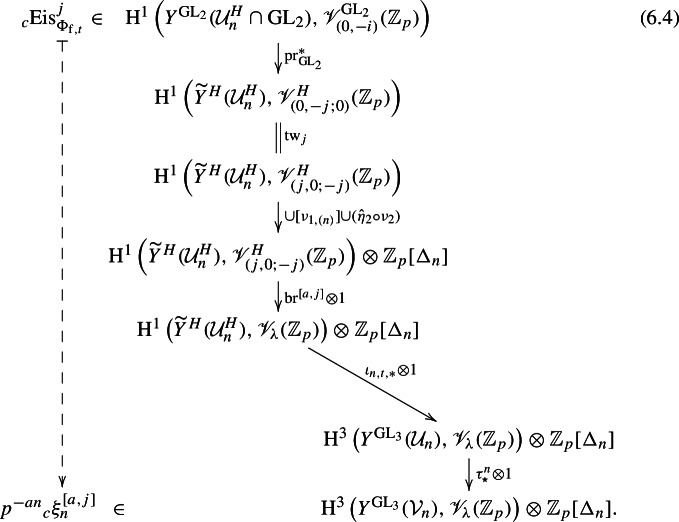


#### Varying *n*

If $\mathcal {U}' \subset \mathcal {U}\subset \textrm{GL}_3(\mathbb {A}_{\textrm{f}})$, let $\textrm{pr}_{\mathcal {U}}^{\mathcal {U}'}$ denote the natural projection map $Y^{\textrm{GL}_3}(\mathcal {U}') \rightarrow Y^{\textrm{GL}_3}(\mathcal {U})$. We get associated pullback/pushforward maps on cohomology. Also let $\textrm{norm}^{\Delta _{n+1}}_{\Delta _n}$ denote the natural projection map $\mathbb {Z}_p[\Delta _{n+1}] \rightarrow \mathbb {Z}_p[\Delta _n]$.

##### Theorem 6.10

(Norm relation) For any $n \ge 1$ we have

$$ \left[ \left( \textrm{pr}^{\mathcal {V}_{n+1}}_{\mathcal {V}_n}\right) _\star \otimes \operatorname {norm}_{\Delta _n}^{\Delta _{n+1}}\right] \left( \xi _{n+1}^{[a,j]}(-)\right) = \left[ U_{p,1}' \otimes 1\right] \cdot \xi _{n}^{[a,j]}(-) $$

as elements of $\textrm{H}^3(Y^{\textrm{GL}_3}(\mathcal {V}_n), \mathscr {V}_\lambda (\mathbb {Z}_p)) \otimes _{\mathbb {Z}_p} \mathbb {Z}_p[\Delta _n].$

##### Proof

This is an instance of [36, Prop. 4.5.2], elaborated in §4.6 ‘*The Betti setting*’ and §5.2.3 *op. cit*. It is an analogue of [41, Thm. 3.13]. As this result is key to our construction, for the convenience of the reader we sketch the proof in this special case, translating the notation of [36] into our setting. We shall drop the indices [*a*, *j*] and *c* here for brevity.

We first prove an analogue for the elements $z_n$ (as in [36, Prop. 4.5.1]). We claim that

6.5 $$\begin{aligned} \left[ 1 \otimes \operatorname {norm}_{\Delta _n}^{\Delta _{n+1}}\right] (z_{n+1}) = p^{a}\left[ \left( \operatorname {pr}_{\mathcal {U}_n}^{\mathcal {U}_{n+1}}\right) ^* \otimes 1\right] (z_n) \end{aligned}$$

as elements of $\textrm{H}^3(Y^{\textrm{GL}_3}(\mathcal {U}_{n+1}), \mathscr {V}_\lambda (\mathbb {Z}_p)) \otimes _{\mathbb {Z}_p} \mathbb {Z}_p[\Delta _n]$. To prove this, one fixes $t \ge n+1$ and checks that the horizontal maps in the diagram 
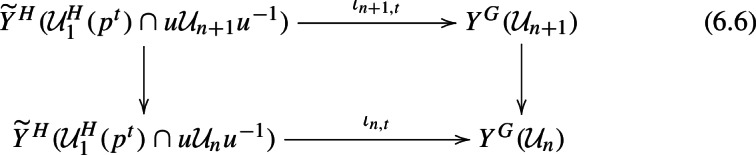
 are injective. (Note: this injectivity is the crucial reason for introducing the twisting map *u*; cf. [41, Rem. 4.12]). By injectivity, the diagram is Cartesian as both vertical maps have degree $p^2$ (using Proposition 6.3). Now, from the definitions we can write6.7The class  (at the top left corner) is a pullback from level $\mathcal {U}_{1}^H(p^t) \cap u \mathcal {U}_n u^{-1}$ (that is, the bottom left corner). Thus the right-hand side of (6.7) is obtained by passing from the bottom-left to top right of (6.6) along the left and top arrows, and (6.5) (where the right-hand side is obtained along the bottom and right arrows) follows from the compatibility of pushforward and pullback in Cartesian diagrams.

Now recall that on cohomology at level $\mathcal {U}_n$, we have $U_{p,1}' = p^{a} \big (\textrm{pr}^{\tau ^{-1} \mathcal {U}_n \tau \cap \mathcal {U}_n}_{\mathcal {U}_n}\big )_\star \circ \tau _\star \circ \big (\textrm{pr}_{\mathcal {U}_n}^{\mathcal {U}_n \cap \tau \mathcal {U}_n\tau ^{-1}} \big )^*.$ One easily sees $\mathcal {U}_{n+1} = \mathcal {U}_n \cap \tau \mathcal {U}_n\tau ^{-1}$. Applying the map $\left[ \left( (\textrm{pr}^{\tau ^{-1} \mathcal {U}_n \tau \cap \mathcal {U}_n}_{\mathcal {U}_n}\big )_\star \circ \tau _\star \right) \otimes 1]\right]$ to (6.5), we see

6.8 $$\begin{aligned} \left[ (\textrm{pr}^{\tau ^{-1} \mathcal {U}_n \tau \cap \mathcal {U}_n}_{\mathcal {U}_n}\big )_\star \circ \tau _\star \otimes \operatorname {norm}_{\Delta _n}^{\Delta _{n+1}}\right] (z_{n+1}) = \left[ U_{p,1}' \otimes 1\right] \cdot z_n \end{aligned}$$

as elements of $\textrm{H}^3(Y^{\textrm{GL}_3}(\mathcal {U}_{n}), \mathscr {V}_\lambda (\mathbb {Z}_p)) \otimes _{\mathbb {Z}_p} \mathbb {Z}_p[\Delta _n].$ One checks that

$$\begin{aligned} (\tau ^n)_\star \circ \left( \textrm{pr}^{\tau ^{-1} \mathcal {U}_n \tau \cap \mathcal {U}_n}_{\mathcal {U}_n}\right) _\star \circ \tau _\star&= \left( \textrm{pr}^{\tau ^{-n-1} \mathcal {U}_n \tau ^{n+1} \cap \tau ^{-n}\mathcal {U}_n\tau ^n }_{\tau ^{-n}\mathcal {U}_n\tau ^n}\right) _\star \circ (\tau ^{n+1})_\star \\&= \left( \textrm{pr}_{\mathcal {V}_n}^{\mathcal {V}_{n+1}}\right) _\star \circ (\tau ^{n+1})_\star , \end{aligned}$$

whilst a simple check on single coset representatives shows that $(\tau ^n)_\star \circ U_{p,1}' = U_{p,1}' \circ (\tau ^n)_\star$. Since $\xi _n = (\tau ^n)_\star (z_n)$ by definition, the result follows by applying $[(\tau ^n)_\star \otimes 1]$ to (6.8). $\square$

### Pairing with a $\textrm{GL}_3$-eigenclass

Recall that in Notation 6.1, we fixed a level subgroup $\mathcal {U}^{(p)} \subset \textrm{GL}_3(\mathbb {A}_{\textrm{f}})$ fixing a Schwartz function $\Phi ^{(p)} \in \mathcal {S}(\mathbb {A}_{\textrm{f}}^{(p),2},\mathbb {Z})$. Let $L/\mathbb {Q}_p$ be a finite extension with ring of integers $\mathcal {O}_L$, and

$$ \phi \in \textrm{H}_{\textrm{c}}^{2}\left( Y^{\textrm{GL}_3}(\mathcal {U}),\mathscr {V}_\lambda ^\vee (\mathcal {O}_L)\right) $$

be any cohomology class. Let $\mathcal {U}_n$ be as in Definition 6.2, , and let $\phi _n$ be the image of $\phi$ in the cohomology of $Y^{\textrm{GL}_3}(\mathcal {V}_n)$, recalling that $\mathcal {V}_n\subset \mathcal {U}$. Recall the pairing $\langle -,-\rangle _{\mathcal {V}_n}$ from Sect. 3.4. Let $\textrm{d}h_{\textrm{f}}$ denote the unramified Haar measure on $H(\mathbb {A}_{\textrm{f}})$, which defines a volume $\textrm{vol}(\mathcal {U}^{(p)}\cap H)$.

#### Proposition 6.11

The element of $\mathcal {O}_L[\Delta _n]$ defined by6.9is independent of $\mathcal {U}^{(p)}$ (for suitably chosen *c*).

#### Proof

If $\mathcal {U}' \subseteq \mathcal {U}\subset \textrm{GL}_3(\mathbb {A}_{\textrm{f}}^{(p)})$ are two levels, with $\mathcal {U}\cap H$ fixing $\Phi ^{(p)}$, then one may check that6.10Let $\mathcal {U}_1^{(p)}, \mathcal {U}^{(p)}_2$ be arbitrary. Then their intersection $\mathcal {U}_3^{(p)}$ also satisfies the conditions, and we see the result by applying (6.10) to $\mathcal {U}_3^{(p)} \subset \mathcal {U}_1^{(p)}, \mathcal {U}_2^{(p)}$. $\square$

For finite-slope eigenclasses, we can also vary *n*.

#### Corollary 6.12

Suppose $$U_{p,1}\phi = \alpha \phi $$, with $$0 \ne \alpha \in \mathcal {O}_L$$.

(i) The element  is a well-defined distribution on $\mathbb {Z}_p^{\times }$ (a linear functional on locally-constant functions).
(ii) If $v_p(\alpha )=0$, then  is a measure.

#### Proof

By the above remarks, for (i) it suffices to show that6.11By definition, we have $\phi _{n+1} = (\textrm{pr}_{\mathcal {V}_n}^{\mathcal {V}_{n+1}})^*(\phi _n).$ Since $c, [a,j], \Phi ^{(p)}$ and $\mathcal {U}^{(p)}$ are fixed, we drop them from notation for clarity, and see

6.12 $$\begin{aligned} \textrm{norm}^{\Delta _{n+1}}_{\Delta _n}&\left( \alpha ^{-(n+1)} \Big \langle \phi _n, \xi _{n+1} \Big \rangle _{\mathcal {V}_{n+1}} \right) \nonumber \\&= \alpha ^{-(n+1)}\left\langle (\textrm{pr}_{\mathcal {V}_n}^{\mathcal {V}_{n+1}})^*\phi _n, \left[ 1 \otimes \textrm{norm}^{\Delta _{n+1}}_{\Delta _n}\right] \xi _{n+1} \right\rangle _{\mathcal {V}_{n+1}} \nonumber \\&= \alpha ^{-(n+1)}\left\langle \phi _n, \left[ (\textrm{pr}_{\mathcal {V}_n}^{\mathcal {V}_{n+1}})_* \otimes \textrm{norm}^{\Delta _{n+1}}_{\Delta _n}\right] \xi _{n+1} \right\rangle _{\mathcal {V}_n} \nonumber \\&= \alpha ^{-(n+1)}\left\langle \phi _n,\ U_{p,1}'\cdot \xi _{n} \right\rangle _{\mathcal {V}_n}\nonumber \\&=\alpha ^{-(n+1)}\left\langle U_{p,1}\cdot \phi _n, \ \xi _{n} \right\rangle _{\mathcal {V}_n} = \alpha ^{-n}\big \langle \phi _n, \xi _{n} \big \rangle _{\mathcal {V}_n}. \end{aligned}$$

In (6.12) we use Theorem 6.10, in the penultimate step use that $U_{p,1}$ and $U_{p,1}'$ are adjoint under $\langle -,-\rangle$, and the final equality uses $U_{p,1}\phi = \alpha \phi$. We conclude (i) after rescaling by $\operatorname {vol}(\mathcal {U}^{(p)} \cap H)$.

Part (ii) is immediate as  for all *n*. $\square$

### Getting rid of *c*

We fix $\Phi ^{(p)}$ and $\mathcal {U}^{(p)}$ and, for now, drop them from notation. We also fix $\phi$ as above, with $U_{p,1}\phi = \alpha \phi$, with $v_p(\alpha ) = 0$.

Define ‘non-smoothed’ analogues $\Xi _n^{[a,j]}$ and $\Xi ^{[a,j]}$ of  and  by using $\xi _n^{[a,j]}$ instead of . Since the non-smoothed Eisenstein classes are $\mathbb {Q}_p$- rather than $\mathbb {Z}_p$-valued, these are ostensibly only distributions in $L[\![\mathbb {Z}_p^{\times }]\!]$. We now show that they actually lie in $\mathcal {O}_L[\![\mathbb {Z}_p^{\times }]\!]$.

#### Proposition 6.13

(i) We have, for each *n*,
(ii) If $\eta _2$ is chosen so that $\eta _2 \omega _{\Pi }$ is not congruent mod *p* to any character of *p*-power conductor, then $\Xi ^{[a,j]}(\phi ) \in \mathcal {O}_L[\![\mathbb {Z}_p^{\times }]\!]$, i.e. it is a measure.

#### Proof

(i) This follows from the formula (6.3) above relating  and the non-*c*-version, since the transpose of $\langle c \rangle$ with respect to Poincaré duality will be $\langle c^{-1} \rangle$, which acts on any vector in $\Pi$ as multiplication by $\omega _{\Pi }(c)^{-1}$.

(ii) Choose *c* with $c = 1 \bmod p^n$ and $(\omega _{\Pi } \eta _2)(c) \ne 1 \bmod p$. It follows that the factor relating  and $\Xi ^{[a,j]}_n$ is invertible in $\mathbb {Z}_p[\Delta _n]$. Thus $\Xi ^{[a,j]}_n$ is integral for all *n* and $\Xi ^{[a,j]}$ is a measure. $\square$

The auxiliary character $\eta _2$ was introduced solely for Proposition 6.13. We must now carry it through all notation, but its only contribution in the rest of the paper will be to the periods $\Omega _\Pi ^\pm$.

### The Manin relations: compatibility in varying *j*

#### Theorem 6.14

For $0 \le j \le a$ and $f: \mathbb {Z}_p^{\times } \rightarrow \overline{\mathbb {Q}}_p$, we have a compatibility

$$ \int _{\mathbb {Z}_p^{\times }} f(x) \cdot \textrm{d}\Xi ^{[a,j]}(\phi )(x) = \int _{\mathbb {Z}_p^{\times }} f(x)x^{j} \cdot \textrm{d} \Xi ^{[a,0]}(\phi )(x). $$

#### Proof

We shall prove this by adapting the methods of [38]. However, the result we seek is not quite a direct consequence of the main theorem of *op.cit.*, since the weight $\lambda = (a, 0, -a)$ of our coefficient sheaf is not induced from a 1-dimensional character of the Levi $L_1$ of $P_1$, and our level group does not always contain $L_1(\mathbb {Z}_p)$. So we shall briefly indicate the modifications needed to the theory of *op.cit.* in order to prove the theorem at hand.

Since our classes are built up from the norm-compatible families , it suffices to prove a compatibility for  and  modulo $p^n$, regarded as elements of the module

$$\textrm{H}^3(Y^{\textrm{GL}_3}(\mathcal {V}_n), \mathscr {V}_\lambda (\mathbb {Z}/p^n)) \otimes (\mathbb {Z}/p^n)[\Delta _n].$$

More precisely, we shall prove the following: the map $\operatorname {mom}^j_{\Delta _n}: (\mathbb {Z}/p^n)[\Delta _n] \rightarrow (\mathbb {Z}/p^n)[\Delta _n]$, defined on group elements by sending [*a*] to $a^j[a]$, maps  to .

We have , where $\tau$ denotes $$\left( {\begin{smallmatrix}p \\   &  1\\   & &  1\end{smallmatrix}}\right) $$. The action of $\tau _\star$ on our coefficients is given by a map of $\mathcal {U}_n$-representations

$$\begin{aligned} (\tau ^n)_{\sharp }: V_\lambda (\mathbb {Z}_p) \rightarrow \tau _n^\star \left( V_\lambda (\mathbb {Z}_p)\right) \end{aligned}$$

which is just $p^{an}$ times the action of $\tau ^{-n}$ on $V_\lambda (\mathbb {Q}_p)$; the factor $p^{an}$ is the image of $\tau ^n$ under the the character $\lambda$ (compare Definition 2.5.1 of [38]).

Since $V_\lambda (\mathbb {Z}_p)$ is an admissible lattice, it is a direct sum of eigenspaces for the diagonal torus, corresponding to the weights of $V_\lambda$. The map $(\tau ^n)_\sharp$ acts on each of these by a non-negative power of $p^n$; so on the mod $p^n$ coefficient sheaf $\mathscr {V}_\lambda (\mathbb {Z}/p^n)$, it is zero on all eigenspaces except the highest relative weight space for the torus $Z(L_1)$. So it suffices to prove that the classes  and  have the same image in the highest relative weight space.

Exactly as in [38], the homomorphism $\operatorname {br}^{[a, j]}$ is determined by the image of the highest-weight vector of $V_{(j, 0; -j)}^H$; let us call this vector $f^{a, j}$. If we model $V_\lambda$ as a space of functions on $\bar{N}_1 \backslash G$ taking values in the weight $\lambda$ representation of $L_1$, and satisfying $f(\bar{n} \ell g) = \ell \cdot f(g)$, then the value *f*(*u*) determines $f^{a, j}$ uniquely on $\overline{P}_1 u^{-1} Q_H^0$, which is open in *G*; so $f^{a, j}$ is uniquely determined by $f^{a, j}(u^{-1})$, and this value lies in a one-dimensional subspace of the weight $\lambda$ $L_1$-representation which is independent of *j* (in fact it is the *lowest* weight space). So we may suppose that the $f^{a, j}$ are normalised so that $f^{a, j}(u^{-1})$ is independent of *j*.

Then, exactly as in [38], we obtain a compatibility between moment maps for *G* and for *H* under pushforward, where $H = \textrm{GL}_2 \times \textrm{GL}_1$ and $G = \textrm{GL}_3 \times \textrm{GL}_1$. The moment map for *G* gives the twisting operator $\operatorname {mom}^j_{\Delta _n}$; and the twisting operator on *H* maps the weight 0 Eisenstein class to its weight *j* equivalent, by Kings’ theory of $\Lambda$-adic Eisenstein classes (see [32] for a summary in our present notations). $\square$

## Values of the distribution as global Rankin–Selberg integrals

Let $\Pi$ be a RACAR of $\textrm{GL}_3(\mathbb {A})$ of weight $\lambda = (a,0,-a)$, and let $\sigma _p$ be a finite-slope unramified $P_1$-refinement (as in Sect. 2.6). Recall $\sigma _p$ is determined by $\alpha _p = \sigma _p(p) \ne 0$. We now apply the previous section to attach distributions to appropriate eigenforms in $\Pi _f$, and show, in Proposition 7.3, that they compute global $\textrm{GL}_3 \times \textrm{GL}_2$ Rankin–Selberg integrals for $\Pi$ with Eisenstein series.

Let $\varphi _{\textrm{f}}= \otimes \varphi _\ell \in \Pi _{\textrm{f}}$ such that:

- $\varphi _p$ is fixed by $\mathcal {U}_{1,p}^{(P_1)}(p^{r(\Pi ,\alpha _p)})$ and $U_{p,1}\varphi _p = p^{a+1}\alpha _p \cdot \varphi _p$ (cf. Proposition 2.24); and
- $W_{\varphi _{\textrm{f}}}$ is algebraic, so that we get a class , for $L/\mathbb {Q}_p$ sufficiently large and $\phi _\Pi , \Theta _\Pi$ as in Sect. 3.3, normalised as in Sect. 3.3.3.

We fix tame data $\Phi ^{(p)} \in \mathcal {S}(\mathbb {A}_{\textrm{f}}^{(p),2},\mathbb {Z})$ and $\mathcal {U}^{(p)} \subset \textrm{GL}_3(\mathbb {A}_{\textrm{f}}^{(p)})$ such that $\varphi _{\textrm{f}}$ (resp. $\Phi ^{(p)}$) is fixed by $\mathcal {U}^{(p)}$ (resp. $\mathcal {U}^{(p)}\cap H$). These choices fix $\Phi _{\textrm{f},n}$ and $\mathcal {V}_n$, but we otherwise drop them from notation throughout this section.

By Proposition 6.13, attached to all this data, and a suitable fixed auxiliary character $\eta _2$, is a distribution $\Xi ^{[a,j]}(\phi _{\varphi _{\textrm{f}}}) \in L[\![\mathbb {Z}_p^{\times }]\!]$; and when $\sigma _p$ is $P_1$-ordinary, $\Xi ^{[a,j]}(\phi _{\varphi _{\textrm{f}}}) \in \mathcal {O}_L[\![\mathbb {Z}_p^{\times }]\!]$ is a measure.

### Values of the distribution

Let $\eta$ be a Dirichlet character of conductor $p^{n_1}$, and let $n = \textrm{max}(n_1,1)$. Consider $\eta$ as a character on $\Delta _n = (\mathbb {Z}/p^n\mathbb {Z})^{\times }$, and lift it it a character on $\mathbb {Z}_p^{\times }$ under the natural surjection. We integrate this against our distribution. This factors through $\Delta _n$, and writing *x* for the variable on $\mathbb {Z}_p^{\times }$, we have

7.1 $$\begin{aligned}&\int _{\mathbb {Z}_p^{\times }} \eta (x) \ \cdot \ \textrm{d}\Xi ^{[a,j]}\big (\phi _{\varphi _{\textrm{f}}}\big )(x) = (p^{a+1}\alpha _p)^{-n} \int _{\Delta _n} \eta (x) \cdot \textrm{d}\Xi _n^{[a,j]}\Big (\phi _{\varphi _{\textrm{f}}}\Big )(x)\nonumber \\&\qquad = (p^{a+1}\alpha _p)^{-n}\big [1 \otimes \eta \big ] \left( \operatorname {vol}(\mathcal {U}^{(p)} \cap H) \cdot \left\langle \left( \phi _{\varphi _{\textrm{f}}}\right) _n, \xi _{n}^{[a, j]} \right\rangle _{\mathcal {V}_n}\right) . \end{aligned}$$

We consider this value in a finite extension of $\mathbb {Q}_p$, but by construction (via the embedding $\iota$) it actually lies in a number field. For the rest of Sect. 7, we will forget this algebraicity and, abusing notation, consider it just as a complex number.

To study (7.1), we use the description and notation of Remark 6.7. Set $t = n$, and for $x \in \Delta _n$, let $i_{x}: \widetilde{Y}^H_{x} \hookrightarrow \widetilde{Y}^H(\mathcal {U}_{n}^H)$ be the natural inclusion. By the remark, the coefficient of [*x*] in $\zeta _n^{[a,j]}$ is

$$ \sum _{y \in (\mathbb {Z}/D)^{\times }} \eta _2(y) \cdot p^{an} \ \tau _\star ^n \circ u_\star \circ \iota _{\star } \circ \textrm{br}^{[a,j]}_\star \circ \textrm{tw}_{j} \circ i_{x,y}^\star (\textrm{Eis}_{\Phi _{\textrm{f}}^{n}}^{j}). $$

Substituting, we see that (7.1) is equal to

7.2 $$\begin{aligned}&= \tfrac{ p^{an}\operatorname {vol}(\mathcal {U}^{(p)} \cap H)}{(p^{a+1}\alpha _p)^n} \sum _{x \in \Delta _n} \eta (x)\nonumber \\&\hspace{10pt}\times \sum _{y \in (\mathbb {Z}/D)^{\times }} \eta _2(y)\cdot \left\langle \left( \phi _{\varphi _{\textrm{f}}}\right) _n, \ \tau _\star ^n u_\star \iota _{\star } \textrm{br}^{[a,j]}_\star \textrm{tw}_{j} i_{x,y}^\star (\textrm{Eis}_{\Phi _{\textrm{f}}^{2n}}^{j}) \right\rangle _{\mathcal {V}_n}\nonumber \\&= \tfrac{ \operatorname {vol}(\mathcal {U}^{(p)} \cap H)}{p^{n}\alpha _p^n} \sum _{x \in \Delta _n} \eta (x)\end{aligned}$$

7.3 $$\begin{aligned}&\hspace{10pt}\times \sum _{y \in (\mathbb {Z}/D)^{\times }} \eta _2(y) \cdot \left\langle \textrm{br}^{[a,j],\star } \iota ^{\star } u^\star \tau ^{n,\star }[\left( \phi _{\varphi _{\textrm{f}}}\right) _n], \ \textrm{tw}_{j} i_{x,y}^\star (\textrm{Eis}_{\Phi _{\textrm{f}}^{2n}}^{j}) \right\rangle _{\widetilde{Y}^H_{x}}, \end{aligned}$$

via adjointness of pushforward and pullback under the Poincaré duality pairing.

### Cup products as integrals

We now express these cup products as integrals. This follows a standard, if technical, procedure (cf. [45, Lem. 1.3], [46, Lem. 3.2]). This technicality will manifest itself exclusively in a local zeta integral at infinity.

#### The differential form for $\Pi$

Recall $\zeta _\infty \in \textrm{H}^2\big (\mathfrak {gl}_3,K_{3,\infty }^\circ ; \Pi _\infty \otimes V_{\lambda }^{\vee }(\mathbb {C})\big )$ from (2.2). We have

$$ \textrm{H}^i\big (\mathfrak {gl}_3,K_{3,\infty }^\circ ; \Pi _\infty \otimes V_{\lambda }^{\vee }\big ) \subset \bigwedge  ^i\left( \mathfrak {gl}_3/\mathfrak {K}_{3,\infty }^\circ \right) ^\vee \otimes \Pi _\infty \otimes V_\lambda ^\vee (\mathbb {C}), $$

where $\mathfrak {K}_{3,\infty }^\circ = \textrm{Lie}(K_{3,\infty }^\circ )$. Choose (arbitrary) bases $\{\delta _1',...\delta _5'\}$ of $\left( \mathfrak {gl}_3/\mathfrak {K}_{3,\infty }^\circ \right) ^\vee$ and $\{v_\alpha \}$ of $V_\lambda ^\vee (\mathbb {C})$. Given these choices, there exist vectors $\varphi _{\infty ,r,s,\alpha } \in \Pi _\infty$ such that

$$ \zeta _\infty = \sum _{r,s = 1,...,5} \sum _{\beta } \big [\delta _r' \wedge \delta _s'\big ] \otimes \varphi _{\infty ,r,s,\alpha } \otimes v_\alpha . $$

Letting , we see the differential form associated to $\phi _{\varphi _{\textrm{f}}} = \phi _\Pi (\varphi _{\textrm{f}})/\Theta _\Pi$ is

7.4 $$\begin{aligned} \Theta _\Pi ^{-1}\sum _{r,s = 1,...,5} \sum _{\beta } \big [\delta _r' \wedge \delta _s'\big ] \otimes \varphi _{r,s,\alpha } \otimes v_\alpha \in \bigwedge {}^i\left( \mathfrak {gl}_3/\mathfrak {K}_{3,\infty }^\circ \right) ^\vee \otimes \Pi \otimes V_\lambda ^\vee (\mathbb {C}). \end{aligned}$$

#### The Eisenstein differential

By Corollary 5.6, the differential on $Y^{\textrm{GL}_2}(\mathcal {U}_{n}^{H}\cap \textrm{GL}_2)$ associated to $\textrm{Eis}_{{\Phi _{\textrm{f}}}}^{j}$ is $-\mathcal {E}_{{\Phi _{\textrm{f}}}}^{j+2}(g) \cdot (\textrm{d}z)^{\otimes j} \cdot \textrm{d}\tau$, recalling $\mathcal {E}_{{\Phi _{\textrm{f}}}}^{j+2}(g) \in I(\Vert \cdot \Vert ^{-1/2}, \Vert \cdot \Vert ^{j+1/2})$ (5.6).

For comparison with the $\textrm{GL}_3$ setting, and with [45, 46], it is convenient to rephrase this in the (inexplicit) language of Sect. 7.2.1. Combining the above discussion, we see we may choose bases $\{\delta _1,\delta _2\}$ of $(\mathfrak {gl}_2/\mathfrak {K}_{2,\infty }^\circ )^\vee$ (corresponding to $\textrm{d}\tau$) and $\{w_\beta ^{[j]}\}$ of $V_{(0,-j)}^{\textrm{GL}_2}(\mathbb {C})$ (corresponding to $(\textrm{d}z)^{\otimes j}$) such that as a differential form on $Y^{\textrm{GL}_2}$, we may describe $\textrm{Eis}_{{\Phi _{\textrm{f}}}}^{j}$ as

7.5 $$\begin{aligned} \Big [\delta _1 + \delta _2\Big ] \otimes \mathcal {E}^{j+2}_{{\Phi _{\textrm{f}}}} \otimes \Big [\sum _\beta w_\beta ^{[j]}\Big ] \in \left( \mathfrak {gl}_2/\mathfrak {K}_{2,\infty }^\circ \right) ^\vee \otimes I\Big (\Vert \cdot \Vert ^{-\tfrac{1}{2}}, \Vert \cdot \Vert ^{j+\tfrac{1}{2}}\Big ) \otimes V_{(0,-j)}^{\textrm{GL}_2}(\mathbb {C}) \end{aligned}$$

(where $\mathcal {E}_{{\Phi _{\textrm{f}}}}^{j+2}(g) \in I \big (\Vert \cdot \Vert ^{-1/2}, \Vert \cdot \Vert ^{j+1/2}\big )$ by Corollary 5.3 and (5.9), with $\eta = 1$).

#### The cup product on components

We pass to *H*. We identify the basis elements $\{\delta _1,\delta _2\}$ with their image under the natural pullback $\left( \mathfrak {gl}_2/\mathfrak {K}_{2,\infty }^\circ \right) ^\vee \rightarrow \left( \textrm{Lie}(H(\mathbb {R}))/\textrm{Lie}(K_{H,\infty }^\circ \iota ^{-1}(Z_{G,\infty }^\circ ))\right) ^\vee$, and then extend to a basis $\{\delta _1,\delta _2,\delta _3\}$ of this latter space. By construction, the pullback of $\textrm{Eis}_{{\Phi _{\textrm{f}}}}^{j}$ to $\widetilde{Y}^H$ corresponds to a differential in which only $\delta _1$ and $\delta _2$ appear.

Since the basis $\{\delta _i'\}$ of $\left( \mathfrak {gl}_3/\mathfrak {K}_{3,\infty }^\circ \right) ^\vee$ was arbitrary, we may rescale so that under the map

$$ \iota ^*: \left( \mathfrak {gl}_3/\mathfrak {K}_{3,\infty }^\circ \right) ^\vee \longrightarrow \left( \textrm{Lie}(H(\mathbb {R}))/\textrm{Lie}(K_{H,\infty }^\circ \iota ^{-1}(Z_{G,\infty }^\circ ))\right) ^\vee , $$

we have $\iota ^*(\delta _i') = \delta _i$ for $i = 1,2,3$ and $\iota ^*(\delta _i') = 0$ for $i = 4,5$ (cf. [46, p.277]).

Recall $\langle -,-\rangle _{a,j}$ from (4.4). By definition,

$$\begin{aligned}&\Big \langle \textrm{br}^{[a,j],\star } \iota ^{\star } u^\star \tau ^{n,\star }[\left( \phi _{\varphi _{\textrm{f}}}\right) _n], \ \textrm{tw}_{j} i_{x}^\star (\textrm{Eis}_{\Phi _{\textrm{f}}^{2n}}^{j}) \Big \rangle _{\widetilde{Y}^H_{x,y}}\\&\qquad \qquad = \int _{\widetilde{Y}^H_{x,y}} \bigg [ \Theta _\Pi ^{-1}\sum _{r,s,t = 1}^3 \sum _{\alpha ,\beta } \left\langle v_\alpha , w_\beta ^{[j]}\right\rangle _{a,j} \cdot \varphi _{r,s,\alpha }\Big (\iota (h)u\tau ^{n}\Big ) \\&\qquad \qquad \qquad \qquad \times \mathcal {E}_{{\Phi _{\textrm{f}}}}^{j+2} \big (\textrm{pr}_1(h)\big ) \cdot \Vert \nu _1^{-j}(h)\Vert \cdot \big [\delta _r\wedge \delta _s \wedge \delta _t\big ]\bigg ]. \end{aligned}$$

Since only $\delta _1$ and $\delta _2$ arise in the Eisenstein differential, the only non-zero terms in the sum over *r*, *s*, *t* are $rst = 132,231$. As $\widetilde{\mathcal {H}}_H$ is 3-dimensional, we identify $\delta _1\wedge \delta _2\wedge \delta _3$ with a fixed choice of Haar measure $\textrm{d}h_\infty$ on $H(\mathbb {R})$. The expression becomes

7.6 $$\begin{aligned} = \Theta _\Pi ^{-1}\sum _{r,s,t} \varepsilon _{rst} \sum _{\alpha ,\beta } \left\langle v_\alpha , w_\beta ^{[j]}\right\rangle _{a,j}\int _{\widetilde{Y}_{x,y}^H} \varphi _{r,s,\alpha }\Big (\iota (h)u\tau ^{n}\Big ) \mathcal {E}_{{\Phi _{\textrm{f}}}}^{j+2} \big (\textrm{pr}_1(h)\big ) \cdot \Vert \nu _1^{-j}(h)\Vert \cdot \textrm{d}h, \end{aligned}$$

where $\varepsilon _{231} = 1$, $\varepsilon _{132} = -1$, and $\varepsilon _{rst} = 0$ otherwise; and $\textrm{d}h = \textrm{d}h_{\textrm{f}}\textrm{d}h_\infty$, recalling $\textrm{d}h_{\textrm{f}}$ from Sect. 6.2.

### Passing to the Rankin–Selberg integral

By definition, on $\widetilde{Y}_{x,y}^{H}$ we have $x = \nu _1(h), y =\nu _2(h)$. Moreover, the characters $\eta$ on $\mathbb {Z}_p^{\times }$ and $\eta _2$ on $(\mathbb {Z}/D)^{\times }$ lift to the (finite order) Hecke characters $\widehat{\eta }$ and $\widehat{\eta _2}$ (as in Sect. 2.1). Combining (7.1), (7.2) and (7.6) now gives

$$\begin{aligned}\begin{array}{c} \int _{\mathbb {Z}_p^{\times }} \eta (x) \cdot \textrm{d}\Xi ^{[a,j]}(\phi _{\varphi _{\textrm{f}}})(x,y) = \tfrac{\operatorname {vol}(\mathcal {U}^{(p)} \cap H)}{p^{n}\alpha _{p}^n\Theta _\Pi } \sum _{r,s,t} \varepsilon _{rst} \sum _{\alpha ,\beta } \left\langle v_\alpha , w_\beta ^{[j]}\right\rangle _{a,j}\\ \int _{\widetilde{Y}^H(\mathcal {U}_{n}^H)} \varphi _{r,s,\alpha }\Big (\iota (h)u\tau ^{n}\Big ) \mathcal {E}_{{\Phi _{\textrm{f}}}}^{j+2} \big (\textrm{pr}_1(h)\big ) \cdot \widehat{\eta }\Vert \cdot \Vert ^{-j}(\nu _1(h))\cdot \widehat{\eta _2}(\nu _2(h)) \textrm{d}h. \end{array}\end{aligned}$$

Up to renormalising our choices at infinity (and hence $\textrm{d}h_\infty$), we may take $\textrm{vol}(K_{H,\infty }^\circ ) = 1$. Also cancelling the $\textrm{vol}(\mathcal {U}^{(p)}\cap H)$ – which, at *p*, introduces $\textrm{vol}(\mathcal {U}_{n,p}^H)$ – we thus obtain

$$\begin{aligned}\begin{array}{c} = \tfrac{\operatorname {vol}(\mathcal {U}_{n,p}^H)^{-1}}{{p^{n}\alpha _{p}^n} \Theta _\Pi } \sum _{r,s,t} \varepsilon _{rst} \sum _{\alpha ,\beta } \langle v_\alpha , w_\beta ^{[j]}\rangle _{a,j} \int _{H(\mathbb {Q})\backslash H(\mathbb {A})/\mathbb {R}_{>0}} \varphi _{r,s,\alpha }\Big (\iota (h)u\tau ^{n}\Big ) \\ \times \mathcal {E}_{{\Phi _{\textrm{f}}}}^{j+2} \big (\textrm{pr}_1(h)\big ) \cdot \widehat{\eta }\Vert \cdot \Vert ^{-j}(\nu _1(h)) \cdot \widehat{\eta _2}(\nu _2(h)) \textrm{d}h, \end{array}\end{aligned}$$

where $z \in \mathbb {R}_{>0}$ embeds as $$[\left( {\begin{smallmatrix}z &  \\  &  z\end{smallmatrix}}\right) , z]$$. Write $h = (\gamma , z) \in \textrm{GL}_2(\mathbb {A}) \times \textrm{GL}_1(\mathbb {A})$, so $\nu _1(h) = \tfrac{\det (\gamma )}{z}$, and

$$\begin{aligned}&= \tfrac{\operatorname {vol}(\mathcal {U}_{n,p}^H)^{-1}}{{p^{n}\alpha _{p}^n} \Theta _\Pi } \sum _{r,s,t} \varepsilon _{rst} \sum _{\alpha ,\beta } \langle v_\alpha , w_\beta ^{[j]}\rangle _{a,j} \int _{H(\mathbb {Q})\backslash H(\mathbb {A})/\mathbb {R}_{>0}} \varphi _{r,s,\alpha }\Big (\iota (\gamma ,z)u\tau ^{n}\Big ) \\&\hspace{120pt}\times \mathcal {E}_{{\Phi _{\textrm{f}}}}^{j+2} \big (\gamma \big ) \cdot \widehat{\eta }\Vert \cdot \Vert ^{-j}\left( \tfrac{\det (\gamma )}{z}\right) \cdot \widehat{\eta _2}(z) \textrm{d}(\gamma ,z)\\&= \tfrac{\operatorname {vol}(\mathcal {U}_{n,p}^H)^{-1}}{p^{n}\alpha _{p}^n \Theta _\Pi } \sum _{r,s,t} \varepsilon _{rst} \sum _{\alpha ,\beta } \langle v_\alpha , w_\beta ^{[j]}\rangle _{a,j} \int _{H(\mathbb {Q})\backslash H(\mathbb {A})/\mathbb {R}_{>0}} \varphi _{r,s,\alpha } \Big (\iota \left( \tfrac{\gamma }{z},1\right) u\tau ^{n}\Big )\widehat{\omega }_\Pi (z) \\&\hspace{120pt}\times \mathcal {E}_{{\Phi _{\textrm{f}}}}^{j+2} \big (\gamma \big ) \cdot \widehat{\eta }\Vert \cdot \Vert ^{-j}\Big (\det \left( \tfrac{\gamma }{z}\right) \Big ) \cdot \widehat{\eta }\widehat{\eta _2}\Vert \cdot \Vert ^{-j}(z) \textrm{d}(\gamma ,z), \end{aligned}$$

noting $\tfrac{\det (\gamma )}{z} = z\cdot \det \big (\tfrac{\gamma }{z}\big )$. Now make the change of variables $g = \gamma /z$: this identifies $H(\mathbb {A})$ with itself, but now $\mathbb {R}_{>0}$ embeds as $$z \mapsto [\left( {\begin{smallmatrix}1 &  \\  &  1\end{smallmatrix}}\right) , z]$$, so the domain becomes

$$ \textrm{GL}_2(\mathbb {Q})\backslash \textrm{GL}_2(\mathbb {A}) \times \mathbb {Q}^{\times }\backslash \mathbb {A}^{\times }/\mathbb {R}_{>0} = \big [\textrm{GL}_2(\mathbb {Q})\backslash \textrm{GL}_2(\mathbb {A})\big ] \times \widehat{\mathbb {Z}}^{\times }. $$

By translation-invariance of Haar measures, we have $dg = d\gamma$. As $\Vert z\Vert ^{-j} = 1$ for $z \in \widehat{\mathbb {Z}}^{\times }$, we get

7.7 $$\begin{aligned}&= \tfrac{\operatorname {vol}(\mathcal {U}_{n,p}^H)^{-1}}{p^{n}\alpha _{p}^n \Theta _\Pi } \sum _{r,s,t} \varepsilon _{rst} \sum _{\alpha ,\beta } \langle v_\alpha , w_\beta ^{[j]}\rangle _{a,j} \int _{[\textrm{GL}_2(\mathbb {Q})\backslash \textrm{GL}_2(\mathbb {A})] \times \widehat{\mathbb {Z}}^{\times }} \varphi _{r,s,\alpha } \Big (\iota \left( g,1\right) u\tau ^{n}\Big ) \nonumber \\&\hspace{120pt}\times \mathcal {E}_{{\Phi _{\textrm{f}}}}^{j+2} \big (g z\big ) \cdot \widehat{\omega }_\Pi \widehat{\eta }\widehat{\eta _2}(z) \cdot \widehat{\eta }\Vert \cdot \Vert ^{-j}\big (\det \left( g\right) \big ) \textrm{d}(g,z), \end{aligned}$$

#### Lemma 7.1

Let $k \ge 0$ and $\chi$ be a Dirichlet character. Then we have

$$ \int _{\widehat{\mathbb {Z}}^{\times }} \mathcal {E}_{{\Phi _{\textrm{f}}}}^{j+2}(gz)\widehat{\chi }(z)\textrm{d}z = E_{\Phi }^{j+2,\chi }(g) = \Vert \det g\Vert ^{\tfrac{j}{2}} E_\Phi \big (g; \widehat{\chi }, -\tfrac{j}{2}\big ). $$

#### Proof

By definition, for $z \in \widehat{\mathbb {Z}}^{\times }$ we have $\mathcal {E}_{{\Phi _{\textrm{f}}}}^{j+2}(gz) = \mathcal {E}_{z\cdot {\Phi _{\textrm{f}}}}^{j+2}(g)$, where *z* acts as $$\left( {\begin{smallmatrix}z &  \\  &  z\end{smallmatrix}}\right) $$. Substituting the definitions, we see that the left-hand side is $\mathcal {E}_{R_\chi ({\Phi _{\textrm{f}}})}^{j+2}(g) = \mathcal {E}_{{\Phi _{\textrm{f}}}}^{j+2,\chi }(g)$, where the equality follows from Definition 5.1(iii). We conclude by (5.8) (giving the first equality) and (5.7) (the second). $\square$

Taking $\chi = \omega _\Pi \eta \eta _2$, and collapsing (7.7) with Lemma 7.1, we conclude

$$\begin{aligned}\begin{array}{c} \int _{\mathbb {Z}_p^{\times }} \eta (x) \cdot \textrm{d}\Xi ^{[a,j]}(\phi _{\varphi _{\textrm{f}}})(x) = \tfrac{\operatorname {vol}(\mathcal {U}_{n,p}^H)^{-1}}{p^{n}\alpha _{p}^n \Theta _\Pi } \sum _{r,s,t} \varepsilon _{rst} \sum _{\alpha ,\beta } \langle v_\alpha , w_\beta ^{[j]}\rangle _{a,j}\\ \int _{\textrm{GL}_2(\mathbb {Q})\backslash \textrm{GL}_2(\mathbb {A})} \varphi _{r,s,\alpha } \Big (\iota (g,1)u\tau ^{n}\Big ) \cdot E_{\Phi }\big (g; \widehat{\omega }_\Pi \widehat{\eta }\widehat{\eta _2}, -\tfrac{j}{2}\big ) \cdot \widehat{\eta }(\det g) \Vert \det g\Vert ^{-\tfrac{j}{2}} \textrm{d}g. \end{array}\end{aligned}$$

#### Definition 7.2

Let $\Pi$ be a unitary automorphic representation of $\textrm{GL}_3(\mathbb {A})$, and $\chi _1$ and $\chi _2$ be Dirichlet characters. For $\varphi \in \Pi$ and $\Phi \in \mathcal {S}(\mathbb {A}^2,\mathbb {C})$, define

Summarising all of the above, we have shown:

#### Proposition 7.3

Let $\eta$ be a Dirichlet character of conductor $p^n$. Then

$$\begin{aligned}&\int _{\mathbb {Z}_p^{\times }} \eta (x) \cdot \textrm{d}\Xi ^{[a,j]}(\phi _{\varphi _{\textrm{f}}})(x) = \frac{\operatorname {vol}(\mathcal {U}_{n,p}^H)^{-1}}{p^{n}\alpha _{p}^n \Theta _\Pi } \sum _{r,s,t} \varepsilon _{rst}\\&\quad \sum _{\alpha ,\beta } \langle v_\alpha , w_\beta ^{[j]}\rangle _{a,j} \mathcal {Z}\Big (u\tau ^n\cdot \varphi _{r,s,\alpha }, \Phi ; \eta , \omega _\Pi \eta \eta _2, \tfrac{1-j}{2},-\tfrac{j}{2}\Big ). \end{aligned}$$

Exactly as in [45, Prop. 3.1] (following the proof of [24, Prop. 3.3]), for $\Re (s) \gg 0$ the integral $\mathcal {Z}(-)$ has an Eulerian factorisation in terms of local zeta integrals, namely

7.8 $$\begin{aligned} \mathcal {Z}\big (\varphi , \Phi ; \chi _1,\chi _2,s_1,s_2\big ) = \prod _v Z_v\big (W_{\varphi ,v}, \Phi _v; \widehat{\chi }_{1,v},\widehat{\chi }_{2,v},s_1,s_2\big ). \end{aligned}$$

We will define and study the local integrals $Z_v(-)$ in the next section (Sect. 8).

We emphasise that in Proposition 7.3, $\varphi _{\textrm{f}}$ is the finite part of each the $\varphi _{r,s,\alpha }$ (i.e. the $\varphi _{r,s,\alpha }$ differ *only* at infinity). This will allow us to move both sums into the local zeta integral at infinity.

## Local zeta integrals

Throughout this section we work locally at a place *v* of $\mathbb {Q}$, and largely drop *v* from notation.

### $\textrm{GL}_2$ principal series

Given $\Phi \in \mathcal {S}(\mathbb {Q}_v^2,\mathbb {C})$, continuous $\chi : \mathbb {Q}_v^{\times } \rightarrow \mathbb {C}^{\times }$, and $g \in \textrm{GL}_2(\mathbb {Q}_v)$, we define a local Godement–Siegel section $f_\Phi (g;\chi ,s)$, exactly as in Sect. 5.2.1 (but with the integral over $\mathbb {Q}_v^{\times }$ rather than $\mathbb {A}^{\times }$). We let $W_\Phi (g;\chi ,s)$ be its Whittaker transform as in [37, §8.1], defined by analytic continuation of an integral convergent for $\Re (s) \gg 0$; the function $W_{\Phi }$ is entire, while $f_{\Phi }$ has the same poles as $\Phi (0, 0) \cdot L(\chi , 2s)$. Note the maps $\Phi \rightarrow f_{\Phi }, \Phi \rightarrow W_\Phi$ have the equivariance property

$$\begin{aligned} f_{h\Phi }(g; \chi , s)&= |\det h|^{-s} f_{\Phi }(gh; \chi , s),\\ W_{h\Phi }(g; \chi , s)&= |\det h|^{-s} W_{\Phi }(gh; \chi , s). \end{aligned}$$

For a fixed *s* where the (local) *L*-factor $L(\chi , 2\,s) \ne \infty$, the space of functions $f_{\Phi }(-; \chi , s)$ for varying $\Phi$ is exactly the induced representation $I\big (|\cdot |^{s-\tfrac{1}{2}}, |\cdot |^{\tfrac{1}{2}-s}\chi ^{-1}\big )$, while the space of $W_{\Phi }(-; \chi , s)$ is the Whittaker model of this representation (with respect to $\psi ^{-1}$, not $\psi$).

If $L(\chi , 2s) = \infty$, the $f_{\Phi }$ may have poles, and $I\big (|\cdot |^{s-\tfrac{1}{2}}, |\cdot |^{\tfrac{1}{2}-s}\chi ^{-1}\big )$ is non-generic: it has a 1-dimensional subrepresentation spanned by the residues of the $f_{\Phi }$, and the $W_{\Phi }$ lie in the Whittaker model of $I\big ( |\cdot |^{\tfrac{1}{2}-s}\chi ^{-1}, |\cdot |^{s-\tfrac{1}{2}}\big )$ instead.

### A two-parameter zeta integral

For Sects. 8.2 and 8.3 only, let $\pi$ be any generic representation of $\textrm{GL}_3(\mathbb {Q}_v)$, and let $\chi _1, \chi _2$ be two smooth characters of $\mathbb {Q}_v^{\times }$.

#### Definition 8.1

For $s_1, s_2$ complex numbers, $W \in \mathcal {W}_{\psi }(\pi )$, and $\Phi \in \mathcal {S}(\mathbb {Q}_v^2,\mathbb {C})$, we set

$$\begin{aligned}&Z(W, \Phi ; \chi _1, \chi _2, s_1, s_2)\\&\quad = \int _{(N_2 \backslash \textrm{GL}_2)(\mathbb {Q}_v) } W(\iota (g, 1)) W_{\Phi }(g; \chi _2, s_2) \chi _1(\det g)|\det g|^{s_1 - \tfrac{1}{2}}\ \textrm{d}g, \end{aligned}$$

which is convergent for $\Re (s_1) \gg 0$ (for fixed $s_2$) and has meromorphic continuation to all $s_1$ and $s_2$, as a rational function in $\ell ^{(\pm s_1 \pm s_2)}$ if $v = \ell$ is a finite place.

#### Theorem 8.2

(Jacquet, Piatetski-Shapiro, Shalika)

(i) The function  is entire as a function of the $s_i$, and is a polynomial in $\ell ^{(\pm s_1 \pm s_2)}$ if $v = \ell$ is a finite place.
(ii) The ideal generated by these functions for varying $(W, \Phi )$ is the unit ideal. In particular, if *v* is a finite place, there exist finite collections $\{W_i\}_{i\in I}$ and $\{\Phi _i\}_{i\in I}$ defined over $\overline{\mathbb {Q}}$ such that $\sum _i\widetilde{Z}(W_i,\Phi _i; \chi _1,\chi _2,s_1,s_2) = 1$ for all $s_1,s_2$.
(iii) If $\pi$ and the $\chi _i$ are unramified, and *W* and $\Phi$ are the normalised spherical data, then $\widetilde{Z}(W,\Phi ; \chi _1,\chi _2,s_1,s_2) = 1$ for all $s_1,s_2$.

Note that for $W_i$ and $\Phi _i$ as in the theorem, we have

$$ \sum _i \widetilde{Z}(W_i,\Phi _i;\chi _1\theta _1,\chi _2\theta _2,s_1,s_2) = 1 $$

for all unramified characters $\theta _1,\theta _2$ (since we can move such $\theta _j$ into the $s_j$).

#### Proof

We know that, for each $s_2$, the functions $W_{\Phi }(-; \chi _2, s_2)$ for varying $\Phi$ form the Whittaker model of the representation $\Pi ' = I(|\cdot |^{s_2 - 1/2}, |\cdot |^{1/2 - s_2} \chi _2^{-1})$; and evidently the $W(g)\chi _1(\det g)$ span the Whittaker model of $\pi \times \chi _1$. So, by the results of [27], the greatest common divisor of the $Z(-;\chi _1, \chi _2, s_1, s_2)$, as functions of $s_1$, is the *L*-factor

$$ L(\pi \times \Pi ' \times \chi _1, s_1) = L(\pi \times \chi _1, s_1 + s_2 - \tfrac{1}{2}) L(\pi \times \chi _1\chi _2^{-1}, s_1 - s_2 + \tfrac{1}{2}), $$

where the latter equality follows from the compatibility of Rankin–Selberg *L*-factors with parabolic induction (also proved in *op.cit.*).

So, for each fixed $s_2$, the normalised integrals $\widetilde{Z}(-; \chi _1, \chi _2, s_1, s_2)$ generate the unit ideal. As these functions are meromorphic in $s_1 \pm s_2$, they in fact generate the unit ideal in $\mathbb {C}[\ell ^{\pm s_1 \pm s_2}]$. $\square$

Note that

$$\begin{aligned} Z(W, \Phi ; \chi _1, \chi _2, s_1, 1-s_2) = Z(W, \hat{\Phi }; \chi _1 \chi _2^{-1}, \chi _2^{-1}, s_1, s_2). \end{aligned}$$

This is immediate from the functional equation of $W_{\Phi }$, cf. [37, Eq. (8.1)]. The denominator in the definition of $\widetilde{Z}$ is the same for both sides (the factors get swapped) so this relation also holds for $\widetilde{Z}$ in place of *Z*. There is also a functional equation in $s_1$ (for fixed $s_2$), but this is more complicated to state, and we shall not use it here.

### A second zeta-integral

We consider a second zeta-integral, studied in [39] (following a series of earlier works). In this section, we shall assume *v* is a finite place.

#### Definition 8.3

For $(\pi , \chi _1, \chi _2, W, \Phi )$ as before, we define

$$\begin{aligned}&Y(W, \Phi ; \chi _1, \chi _2, s_1, s_2) = \\&\quad \int _{(N_2 \backslash \textrm{GL}_2)(\mathbb {Q}_v)} W\left[ \left( {\begin{smallmatrix} 1 &   &   \\  &   &  1 \\  &  -1 &  \end{smallmatrix}}\right) \iota (g, 1) \right] f_{\Phi }(g; \chi _2, s_2) |\det g|^{s_1 - \tfrac{1}{2}}\chi _1(\det g)\, \textrm{d}g. \end{aligned}$$

This integral converges for $\Re (s_2) \gg 0$ for any fixed $s_1$ [sic!] and extends to a meromorphic function of $s_1$ and $s_2$, which is a a rational function in $\ell ^{\pm s_1 \pm s_2}$ if $v = \ell$ is finite.

#### Relation to the *Z* integral

We will prove the following theorem in an appendix below.

##### Theorem 8.4

We have the identity

$$ Y(W, \Phi ; \chi _1, \chi _2, s_1, s_2) = \gamma (\pi \times \chi _1/\chi _2, s_1 - s_2 + \tfrac{1}{2})\cdot Z(W, \Phi ; \chi _1, \chi _2, s_1, s_2), $$

where

$$\begin{aligned} \gamma (\pi , s) = \frac{\varepsilon (\pi , s) L(\pi ^\vee , 1-s)}{L(\pi , s)} \end{aligned}$$

is the local $\gamma$-factor. In particular, the greatest common divisor of the $Y(-)$ as $(W, \Phi )$ vary is the principal ideal generated by the product of *L*-factors

$$ L(\pi \times \chi _1, s_1 + s_2 - \tfrac{1}{2}) \cdot L(\pi ^\vee \times \chi _1^{-1} \chi _2, s_2 - s_1 + \tfrac{1}{2}). $$

#### Torus integrals

We now give an alternative, more “computable” formula for $Y(-)$.

##### Definition 8.5

For $W \in \mathcal {W}(\pi )$, define a function on $\textrm{GL}_2(\mathbb {Q}_v)$ by

$$\begin{aligned}&y(W; \chi _1, \chi _2, s_1, s_2)(g) = \chi _1(\det g)|\det g|^{s_1 - \tfrac{1}{2}} \\&\quad \times \int _{(\mathbb {Q}_v^{\times })^2} W\left[ \left( {\begin{smallmatrix}x \\   &  1\\   & &  y^{-1}\end{smallmatrix}}\right) \left( {\begin{smallmatrix} 1 &   &   \\  &   &  1 \\  &  -1 &  \end{smallmatrix}}\right) \iota (g, 1)\right] \cdot |x|^{s_1 + s_2 - \tfrac{3}{2}} \chi _1(x)\cdot |y|^{s_2 - s_1-\tfrac{1}{2}} \tfrac{\chi _2}{\chi _1}(y) \ \textrm{d}^{\times } x\, \textrm{d}^{\times } y.\end{aligned}$$

The integral converges for $\Re (s_2) \gg 0$ (for fixed $s_1$), as before; more precisely, it converges in some quadrant $\Re (s_2 - s_1)> C, \Re (s_2 + s_1) > C$. A computation shows that it transforms as an element of $I(|\cdot |^{1/2-s_2}, |\cdot |^{s_2 - 1/2} \chi _2)$, which is the dual space of the representation in which $f_{\Phi }(-;\chi _2, s_2)$ lies, and the duality pairing recovers *Y*: that is, we have

8.1 $$\begin{aligned} Y(W, \Phi ; \chi _1, \chi _2, s_1, s_2) = \left\langle y(W; \chi _1, \chi _2, s_1, s_2), f_{\Phi }(-;\chi _2, s_2)\right\rangle . \end{aligned}$$

### Parahoric level test data

We now take $\pi = \Pi _p$, and evaluate (in Theorem 8.7) the local zeta-integral directly for certain specific test data; the integral we consider here is a local factor of the global integral in Proposition 7.3). We suppose that *v* is a finite place, and denote it by *p* (for compatibility with our applications below).

Suppose $\Pi$ has an unramified $P_1$-refinement $\sigma _p$, so there is an irreducible $\textrm{GL}_2$-representation $\sigma _p'$ such that $\sigma _p \times \sigma _p' \hookrightarrow J_{P_1}(\Pi _p)$. Recall $\alpha _p = \sigma _p(p)$, and from Proposition 2.24 that $r(\Pi ,\alpha _p)$ is the conductor of $\sigma _p'$.

Henceforth, we fix the following characters and test data.

#### Notation 8.6

- Let $\chi _1 = \widehat{\eta }_{1, p}$, where $\eta _1$ is a Dirichlet character of conductor $p^{n_1}$ for $n_1 \in \mathbb {Z}_{\ge 0}$ (so that $\chi _1(p) = 1$ and $\chi _1|_{\mathbb {Z}_p^{\times }} = \eta _1^{-1}$). Let $n = \max (1,n_1) \ge 1$.
- Let $\chi _2 = \widehat{\omega }_{\Pi , p} \widehat{\eta }_{1, p}\widehat{\eta }_{2, p}$, where $\eta _2$ is the auxiliary Dirichlet character from Notation 6.5.
- Let $W^{\alpha } \in \Pi _p$ be the $P_1$-stabilised newvector of $\tilde{\Pi }$ as defined in Sect. 2.9, so that $W^{\alpha }$ is stabilized by $\mathcal {U}_{1,p}^{(P_1)}(p^{r(\Pi ,\alpha _p)})$ and lies in the $U_{p,1} = p^{a+1}\alpha _p$ eigenspace.
- Let $$u = \left( {\begin{smallmatrix} 1 &   &  1 \\  &  1 &   \\  &   &  1\end{smallmatrix}}\right) \left( {\begin{smallmatrix} 1 &   &   \\  &   &  -1 \\  &  1 &  \end{smallmatrix}}\right) \in \textrm{GL}_3(\mathbb {Z}_p)$$ as before, and take for *W* the element $u\tau _{ 1}^n \cdot W^{\alpha }$
- Let $\Phi$ be the characteristic function $\Phi _R = \operatorname {ch}( (0, 1) + p^R \mathbb {Z}_p^2)$, for $R = \textrm{max}(n,r(\Pi ,\alpha _p))$.

To ease notation, we will drop the $\widehat{\cdot }$ from local characters, writing for example $\eta _{i,p}$ for $\widehat{\eta }_{i,p}$.

Recall the Coates–Perrin-Riou factor $e_p(\Pi _p \times \eta _{1,p}, -j)$ from Definition 2.16, and its explicit description in our present setting from Example 2.21.

#### Theorem 8.7

We have

$$\begin{aligned}&Y\Big (W, \Phi _R; \chi _1, \chi _2, \tfrac{1-j}{2}, \tfrac{-j}{2}\Big ) \\&\quad =\frac{\alpha _p^n}{p^{2R + n}(1 - p^{-1})(1 - p^{-2})} \cdot e_p(\Pi _p \times \eta _{1,p}, -j) \cdot \mathcal {E}_0 \cdot L(\Pi _p^\vee \times \omega _{\Pi ,p}\eta _{2, p}, 0), \end{aligned}$$

where $\mathcal {E}_0$ is an explicit *p*-adic unit. It follows that

$$\begin{aligned}&Z\Big (W, \Phi _R; \chi _1, \chi _2, \tfrac{1-j}{2}, \tfrac{-j}{2}\Big ) = \\&\qquad \frac{\alpha _p^n }{p^{2R + n}(1 - p^{-1})(1 - p^{-2})} \cdot e_p(\Pi _p \times \eta _{1,p}, -j) \cdot \frac{\mathcal {E}_0 \cdot L(\Pi _p\times \omega _{\Pi ,p}^{-1}\eta _{2, p}^{-1}, 1)}{\varepsilon (\Pi _p\times \omega _{\Pi ,p}^{-1}\eta _{2, p}^{-1},1)}. \end{aligned}$$

The factor $\alpha _p^n$, and the first denominator term (which has an interpretation as the index of a subgroup of $H(\mathbb {Z}_p)$), will naturally cancel out in our *p*-adic interpolation computations. The local *L*-factor will later contribute to the period $\Omega _\Pi ^-$ (see (9.1)).

#### Proof

The statement for *Z* follows immediately from the statement for *Y* and Theorem 8.4.

We focus, then, on *Y*. To save notation we give the proof supposing $\eta _{2, p}$ is trivial; we indicate the necessary modifications for general $\eta _{2,p}$ in Remark 8.9. Since $R \ge 1$, one computes that $f_{\Phi _R}(-, \chi _2, s_2)$ has support in $B_2(\mathbb {Q}_p) K_0(p^R)$, whose measure in the quotient $B_2(\mathbb {Q}_p) \backslash \textrm{GL}_2(\mathbb {Q}_p)$ is $\tfrac{1}{p^R(1+1/p)}$; and its value at the identity is $\tfrac{1}{p^R(1-1/p)}$. Hence for all sufficiently large *R* (depending on *W*) we have

$$\begin{aligned} Y(W, \Phi _R; \chi _1, \chi _2, s_1, s_2) = \tfrac{1}{p^{2R}(1-p^{-2})} y(W; \chi _1, \chi _2, s_1, s_2)(1). \end{aligned}$$

Thus the Whittaker function in the integral $y\left( W; \chi _1, \chi _2, s_1, s_2\right) (1)$ is given by

$$ W^{\alpha }\left( \left( {\begin{smallmatrix}x \\   &  1\\   & &  y^{-1}\end{smallmatrix}}\right) \left( {\begin{smallmatrix} 1 &  &  \\ &  &  1 \\ &  -1 &  \end{smallmatrix}}\right) u \tau _{1}^n\right) = \psi (x) \omega _\Pi (y)^{-1} W^{\alpha } \left( \left( {\begin{smallmatrix} p^n xy &  &  \\ &  y &  \\ &  &  1\end{smallmatrix}}\right) \right) . $$

Hence the integral is given by

$$ \sum _{(a, b) \in \mathbb {Z}^2} W^{\alpha }\left( \left( {\begin{smallmatrix}p^{n+a+b} \\   &  p^b\\   & &  1\end{smallmatrix}}\right) \right) p^{-a(s_1 + s_2 - \tfrac{3}{2})} p^{-b(s_2 - s_1-\tfrac{1}{2})} \int _{\mathbb {Z}_p^{\times }}\psi (p^a x) \chi _1(x) \ \textrm{d}^{\times } x. $$

The values of the Whittaker function $W^{\alpha }$ along the torus are given by Proposition 2.25; they vanish unless $a \ge -n$ and $b \ge 0$. The integral over $\mathbb {Z}_p^{\times }$ is standard: if $\eta _{1,p}$ is ramified, then it is $G(\eta _{1,p}^{-1}) / p^n(1-p^{-1})$ when $a = -n$, and vanishes otherwise; if $\eta _{1,p}$ is unramified (so $n = 1$), then it vanishes for $a < -1$, takes the value $\tfrac{-1}{p-1}$ for $a = -1$, and is 1 for $a \ge 0$.

For non-trivial $\eta _{1,p}$, we thus compute that $Y(W, \Phi _R, \chi _1, \chi _2, s_1, s_2)$ is equal to

$$\begin{aligned}&= \frac{G(\eta _{1,p}^{-1})}{p^{2R+n}(1-p^{-1})(1-p^{-2})} p^{n(s_1 + s_2 - \tfrac{3}{2})} \sum _{b \ge 0} W^{\alpha }\left( \left( {\begin{smallmatrix}p^b \\   &  p^b\\   & &  1\end{smallmatrix}}\right) \right) p^{-b(s_2 - s_1-\tfrac{1}{2})}. \end{aligned}$$

Using Proposition 2.25, the sum is

$$\begin{aligned} \sum _{b \ge 0} W^{\alpha }\left( \left( {\begin{smallmatrix}p^b \\   &  p^b\\   & &  1\end{smallmatrix}}\right) \right) p^{-b(s_2 - s_1-\tfrac{1}{2})}&= \sum _{b \ge 0} W^{\textrm{new}}_{\sigma _p'}\left( \begin{pmatrix}p^b &  \\  &  1\end{pmatrix}\right) p^{-b(s_2 - s_1)}\alpha _p^b\\&= \int _{\mathbb {Q}_p^{\times }}W_{\sigma _p'}^{\textrm{new}}\begin{pmatrix}x &  \\  &  1\end{pmatrix}\sigma _p(x)|x|^{s_2-s_1}d^{\times } x\\&= L(\sigma _p'\times \sigma _p, s_2-s_1+\tfrac{1}{2}), \end{aligned}$$

recalling $\sigma _p \times \sigma _p'$ is our $P_1$-refinement; the last equality is [17, Prop. 6.17]. Thus

$$ Y(W, \Phi _R; \chi _1, \chi _2, s_1, s_2) = \frac{G(\eta _{1,p}^{-1}) p^{n(s_1 + s_2 -\tfrac{3}{2})}}{p^{2R+n}(1-p^{-1})(1-p^{-2})} \cdot L(\sigma _p' \times \sigma _p,s_2 - s_1 + \tfrac{1}{2}). $$

If $\eta _{1,p}$ is trivial, we obtain instead

$$ \frac{\alpha _p}{p^{2R+1}(1 - p^{-1})(1 - p^{-2})} \cdot \frac{(1 - \alpha _p^{-1}p^{-(\tfrac{3}{2}-s_1-s_2)})}{(1 - \alpha _p p^{-(s_1 + s_2 - \tfrac{1}{2})})} \cdot L(\sigma _p' \times \sigma _p, s_2 - s_1 + \tfrac{1}{2}). $$

We see that when $s_1 = \tfrac{1-j}{2}$ and $s_2 = -\tfrac{j}{2}$, then in either case we obtain

$$\begin{aligned}&Y\big (W, \Phi _R; \chi _1, \chi _2, \tfrac{1-j}{2}, -\tfrac{j}{2}\big )\\&\quad = \frac{\alpha _p^n}{p^{2R+n}(1-p^{-1})(1-p^{-2})} \cdot e_p(\Pi _p \times \eta _{1,p}, -j) \cdot L(\sigma _p' \times \sigma _p,0). \end{aligned}$$

To remove the *L*-factor $L(\sigma _p'\times \sigma _p,0)$, let

8.2 $$\begin{aligned} \mathcal {E}_s = \frac{L(\sigma _p' \times \sigma _p,s)}{L(\Pi _p^\vee \times \omega _{\Pi ,p}, s)}. \end{aligned}$$

The statement for *Y* in the theorem follows by replacing $L(\sigma _p'\times \sigma _p,0)$ with $\mathcal {E}_0\cdot L(\Pi _p^\vee \times \omega _{\Pi ,p}, 0)$. It only remains to show $\mathcal {E}_0$ is well-defined and a *p*-adic unit, which we do in Lemma 8.8 below. $\square$

#### Lemma 8.8

If $\Pi _p$ is irreducibly induced from a representation $\alpha \times \sigma$ of $\textrm{GL}_1 \times \textrm{GL}_2$, then we have

$$ \frac{L(\sigma _p' \times \sigma _p, s)}{L(\Pi _p^\vee \times \omega _{\Pi ,p}, s)} = \frac{1}{L(\widehat{\omega }_{\Pi , p} \sigma _p^{-1}, s)} = 1-\omega _{\Pi }(p)\alpha _p^{-1}p^{-s}. $$

If the induction of $\sigma _p \times \sigma _p'$ is reducible, then this ratio is identically 1.

In particular, if $\tilde{\Pi }$ is $P_1$-ordinary, then $\mathcal {E}_0$ is a *p*-adic unit.

#### Proof

For $\textrm{GL}_3$, we have $L(\Pi _p^\vee \times \omega _{\Pi ,p},s) = L(\wedge ^2 \Pi _p, s)$ (see e.g. [31, §1]). If $\Pi _p$ is irreducibly induced, then we have $L(\wedge ^2 \Pi _p, s) = L(\sigma _p' \times \sigma _p, s) L(\wedge ^2 \sigma _p', s)$, and we have $\wedge ^2 \sigma _p' = \omega _{\sigma _p'} = \widehat{\omega }_{\Pi ,p} \sigma _p^{-1}$; and $\widehat{\omega }_{\Pi ,p}(p)\sigma _p^{-1}(p) = \omega _{\Pi }(p)\alpha _p^{-1}$. In the remaining cases, one can write down the Langlands parameter as in Example 2.13 and argue similarly to find that the ratio is 1.

For the final claim, we note that $\mathcal {E}_0$ is either 1 or $1 - \omega _{\Pi }(p)\alpha _p^{-1}$. As $\alpha _p^{-1}$ has valuation $1 + a > 0$ with respect to our choice of embedding into $\overline{\mathbb {Q}}_p$, whilst $\omega _{\Pi }(p)$ is a root of unity, we see $\mathcal {E}_0$ must be a *p*-adic unit. $\square$

#### Remark 8.9

We indicate the changes required if $\eta _{2,p}$ is not trivial. In the sum over $(a,b)\in \mathbb {Z}^2$, we must add $\eta _{2,p}(p^b)$; and in the expression for *Y*, the *L*-value that appears is $L(\sigma _p'\times \sigma _p\eta _{2,p},s_2-s_1+1/2)$. Then $\mathcal {E}_s = L(\sigma _p'\times \sigma _p\eta _{2,p},s)/L(\Pi _p^\vee \times \omega _{\Pi ,p}\eta _{2,p},s)$. In Lemma 8.8 we find the ratio is either 1 or $1-\omega _\pi (p)\eta _2(p)\alpha _p^{-1}p^{-s}$, and conclude as before, since $\eta _2(p)$ is a root of unity.

### Local zeta integrals at infinity

Now consider $v=\infty$. The following is directly analogous to the discussion after [46, Lem. 1.1]. Recall $\Phi _\infty ^{j+2}$ from (5.5), and let $W_\infty \in \mathcal {W}_\psi (\pi _\infty )$.

#### Lemma 8.10

[26]. There exists a polynomial $P_j(W_\infty ; T) \in \mathbb {C}[T]$ such that

$$\begin{aligned} Z(W_\infty ,\Phi _\infty ^{j+2};&\chi _1,\chi _2,s,-\tfrac{j}{2}) \\&= P_j(W_\infty ; s +\tfrac{j}{2}) \cdot L(\pi \times \chi _1, s - \tfrac{j + 1}{2})\cdot L(\pi \times \chi _1\chi _2^{-1}, s+\tfrac{j + 1}{2}). \end{aligned}$$

#### Remark 8.11

Our $P_j(W_\infty ;T)$ is not the direct analogue of $P_{w_\infty ,\psi _\infty }(T)$ from [46]. Instead, it is scaled by a non-zero rational multiple of a power of $\pi$, which depends on *j* but not $W_\infty$. In particular, we incorporate (the analogue of) Mahnkopf’s $A\cdot \pi ^{\flat -l}$ into *P*.

## The *p*-adic *L*-function

We collect our constructions and prove the interpolation formula, completing the proof of Theorem B from the introduction for twists in the “left half” of the critical strip. Fix $\tilde{\Pi }= (\Pi ,\alpha _p)$ a $P_1$-ordinary $P_1$-refined RACAR of $\textrm{GL}_3(\mathbb {A})$ of weight $\lambda = (a,0,-a)$. Recall the auxiliary character $\eta _{2}$ from Notation 6.5.

### Main result

We fix test data at the finite primes using the following recipe.

#### Notation 9.1

- At $v \not \mid p\infty$, choose a finite index set $I_v$, and a collection of Whittaker functions $W_{v, i} \in \mathcal {W}(\Pi _v, E)$ and Schwartz functions $\Phi _{v, i}$ indexed by $i \in I_v$, such that
  $$\begin{aligned} \sum _{i \in I_v} \widetilde{Z}(W_v,\Phi _v; 1,\omega _{\Pi ,v}\eta _{2,v},s_1,s_2) = 1. \end{aligned}$$
  This is possible by Theorem 8.2. We let $\varphi _{v, i} \in \Pi _v$ be such that $\mathcal {W}_\psi (\varphi _{v, i}) = W_{v, i}$. We can (and do) suppose that the $\Phi _{v, i}$ take values in $\mathbb {Z}$, and that for primes such that $\Pi _v$ is unramified, we have $|I_v| = 1$ and the corresponding $W_{v, i}$ and $\Phi _{v, i}$ are the normalized spherical data.
- We define $I = \prod _{v \not \mid p\infty } I_v$ (which is finite since almost all the $I_v$ are singletons), so that tensoring together the $W_{v, i}$ and $\Phi _{v, i}$ gives us a finite collection of vectors $W_i^{(p\infty )} \in \mathcal {W}(\Pi ^{(p)}_{\textrm{f}}, E)$, $\Phi _i^{(p\infty )} \in \mathcal {S}((\mathbb {A}_{\textrm{f}}^{p})^2, \mathbb {Z})$ with
  $$\begin{aligned} \sum _{i \in I} \widetilde{Z}\left( W_i^{(p\infty )}, \Phi _i^{(p\infty )}; 1,\omega _{\Pi }^{(p\infty )} \eta _{2}^{(p\infty )},s_1,s_2\right) = 1. \end{aligned}$$
- At $v = p$, we take $\varphi _p$ such that $\mathcal {W}_\psi (\varphi _p) = W^\alpha$ and $\Phi _p = \Phi _R$ (as in Notation 8.6).

This gives us a finite collection of vectors $\varphi _{\textrm{f}, i} \in \Pi _{\textrm{f}}$ and $\Phi _{\textrm{f}, i} \in \mathcal {S}(\mathbb {A}_{\textrm{f}}^2, \mathbb {Z})$. We fix a choice of level group $\mathcal {U}^{(p)}$ away from *p* fixing all of the $\varphi _{\textrm{f}, i}$, and work at level $\mathcal {U}= \mathcal {U}^{(p)}\mathcal {U}_p$ where $\mathcal {U}_p$ is the subgroup $\mathcal {U}_p^{(P_1)}(p^{r(\Pi _p, \alpha _p)})$ as above. We normalize our periods, as in Sect. 3.3.3 and Sect. 7, so that we have  for all *i*, for $L/\mathbb {Q}_p$ finite (containing *E*).

Recall $\Xi ^{[a,j]}(\phi _{\varphi _{\textrm{f}}},\Phi ^{(p)}) \in \mathcal {O}_L[\![\mathbb {Z}_p^{\times }]\!]$ from Sects. 6.2 and 6.3.

#### Definition 9.2

We write $\iota$ for the involution of $\mathcal {O}_L[\![\mathbb {Z}_p^{\times }]\!]$ corresponding to $x \mapsto x^{-1}$ on $\mathbb {Z}_p^{\times }$.

#### Definition 9.3

The *left-half* *p-adic* *L-function* of $\Pi$ is defined by

#### Remark 9.4

We include the involution $\iota$ in order to obtain a measure with an interpolating property at integers in $[-a, 0]$ rather than [0, *a*], to better match the critical range for the complex *L*-function.

Recall $\mathcal {E}_0 \ne 0$ from (8.2) and Lemma 8.8, and define a period9.1The following interpolation, which proves Conjecture 1.2(i) of the introduction for $n=3$, is our main theorem. The proof will occupy the rest of Sect. 9.

#### Theorem 9.5

Let $\Pi$ be a RACAR of $\textrm{GL}_3(\mathbb {A})$ of weight $(a,0,-a)$, admitting an ordinary $P_1$-refinement at *p*. The *p*-adic *L*-function $L_p^-(\Pi ) \in \mathcal {O}_L[\![\mathbb {Z}_p^{\times }]\!]$ from Definition 9.3 satisfies the following interpolation property: for all $(-j,\eta ) \in {{\,\textrm{Crit}\,}}^-_p(\Pi )$, we have

9.2 $$\begin{aligned} \int _{\mathbb {Z}_p^{\times }} \eta ^{-1}(x)x^{-j} \cdot \textrm{d}L_p^-(\Pi )(x) = e_\infty (\Pi _\infty \times \eta _\infty , -j) e_p(\Pi _p \times \eta _p, -j)\cdot \frac{L^{(p)}(\Pi \times \eta ,-j)}{\Omega _\Pi ^-}. \end{aligned}$$

Here ${{\,\textrm{Crit}\,}}^-_p(\Pi ) = \{(-j,\eta ): 0 \le j \le a, \ \textrm{cond}(\eta ) \mid p^\infty \}$ was defined in Sect. 2.5, $e_\infty$ in Definition 2.6, and $e_p$ in Definition 2.16.

### Interpolation of *L*-values, part I

Recall from Sect. 7.2: our choices of $\zeta _\infty$ (in (2.2)) and bases $v_\alpha ,w_\beta ^{[j]}$ determined forms $\varphi _{\infty ,r,s,\alpha } \in \Pi _\infty$, and we set $\varphi _{r,s,\alpha } = \varphi _{\infty ,r,s,\alpha } \cdot \varphi _{\textrm{f}}$. By Theorem 6.14 and Proposition 7.3, the left-hand side of (9.2) is then

9.3 $$\begin{aligned}&{\mathop {=}\limits ^{5.4}} \int _{\mathbb {Z}_p^{\times }} \eta (x) \cdot \textrm{d}\Xi ^{[a,j]}(\phi _{\varphi _{\textrm{f}}})(x) \nonumber \\&{\mathop {=}\limits ^{7.2}} \frac{1 }{p^{n}\operatorname {vol}(\mathcal {U}_{n,p}^H)\alpha _{p}^n \Theta _\Pi } \sum _{r,s,t} \varepsilon _{rst} \sum _{\alpha ,\beta } \langle v_\alpha , w_\beta ^{[j]}\rangle _{a,j} \mathcal {Z}\Big (u\tau _1^n\cdot \varphi _{r,s,\alpha }, \Phi ; \eta , \omega _\Pi \eta \eta _2, \tfrac{1-j}{2}, -\tfrac{j}{2}\Big ). \end{aligned}$$

Each $\mathcal {Z}(-)$ is a product of local integrals $Z_v(-)$ by (7.8). As the *finite* parts of the $\varphi _{r,s,\alpha }$ are the same, for each $v\not \mid \infty$ the integral $Z_v(-)$ is the same in each summand. Such $Z_v(-)$ is computed in Theorem 8.2 ($v\not \mid p\infty$) and Theorem 8.7 ($v = p$, noting $W = u\tau _1^n \cdot W^\alpha$). We conclude

$$\begin{aligned}&\mathcal {Z}\big (u\tau _1^n \cdot \varphi _{r,s,\alpha }, \Phi ; \eta ,\omega _\Pi \eta \eta _2,\tfrac{1-j}{2},-\tfrac{j}{2}\big ) = \\&Z_\infty \big (W_{\infty , r,s,\alpha }, \Phi _\infty ^{j+2}; \widehat{\eta }_{\infty }, \widehat{\omega }_{\Pi ,\infty }\widehat{\eta }_{\infty }, \tfrac{1-j}{2}, -\tfrac{j}{2}\big ) \times \frac{\alpha _p^n \cdot \mathcal {E}_0 }{p^{2R + n}(1 - p^{-1})(1 - p^{-2})}\\&\qquad \qquad \times e_p(\Pi _p \times \eta _{p}, -j) \cdot L^{(p)}(\Pi \times \eta , -j) \cdot \frac{L(\Pi \times (\omega _{\Pi }\eta _2)^{-1}, 1)}{\varepsilon (\Pi _p \times (\omega _{\Pi ,p}\eta _{2,p})^{-1},1)}, \end{aligned}$$

where $W_{\infty ,r,s,\alpha } = \mathcal {W}_\psi (\varphi _{\infty ,r,s,\alpha }) \in \mathcal {W}_\psi (\Pi _\infty )$ (recalling that $\eta _2$ is even, so $\widehat{\eta }_{2,\infty } = 1$).

We now sweep the sums into the local zeta integral at $\infty$. LetThen

9.4 $$\begin{aligned} (9.3)&= \frac{1 }{p^{n}\operatorname {vol}(\mathcal {U}_{n,p}^H)\alpha _{p}^n \Theta _\Pi } \cdot \frac{\alpha _p^n \cdot \mathcal {E}_0 }{p^{2R + n}(1 - p^{-1})(1 - p^{-2})} \cdot \widetilde{e}_\infty (\zeta _\infty \times \eta _\infty ,-j) \nonumber \\&\quad \times e_p(\Pi _p \times \eta _{p}, -j) \cdot L^{(p)}(\Pi \times \eta , -j) \cdot \frac{L(\Pi \times (\omega _{\Pi }\eta _2)^{-1}, 1)}{\varepsilon (\Pi _p \times (\omega _{\Pi ,p}\eta _{2,p})^{-1},1)}. \end{aligned}$$

We compute $\textrm{vol}(\mathcal {U}_{n,p}^H)^{-1} = p^{2R+2n}(1-p^{-1})(1-p^{-2})$ via Proposition 6.3, whence by (9.1) we find

9.5 $$\begin{aligned} \int _{\mathbb {Z}_p^{\times }} \eta (x)x^{j} \cdot \textrm{d}L_p(\tilde{\Pi })(x) = \widetilde{e}_\infty (\zeta _\infty \times \eta _\infty ,-j) \cdot e_p(\Pi _p \times \eta _{p}, -j)\cdot \frac{L^{(p)}(\Pi \times \eta , -j)}{\Omega _\Pi ^-}. \end{aligned}$$

### Non-vanishing at infinity

It remains to evaluate $\widetilde{e}_\infty (\zeta _\infty \times \eta _\infty ,-j)$. We first show it is non-zero. For each $Z_\infty (W_{\infty ,r,s,\alpha },-)$ in its definition, by Lemma 8.10 we get an associated polynomial $P_j(W_{\infty ,r,s,\alpha };T) \in \mathbb {C}[T]$. DefineThis is (an explicit non-zero multiple of) the analogue of $P_l(s)$ in [46, (3.1)] (cf. Remark 8.11). Combining with Lemma 8.10, we see

$$\begin{aligned} \widetilde{e}_\infty (\zeta _\infty \times \eta _\infty ,-j) = P_j(\zeta _\infty ; \tfrac{1}{2}) \cdot L(\Pi _\infty \times \eta _\infty , -j)\cdot L(\Pi _\infty \times \omega _{\Pi ,\infty }^{-1}, 1). \end{aligned}$$

By [29], we know $P_j(\zeta _\infty ; \tfrac{1}{2}) \ne 0$ (cf. [18, Thm. 2.1]), and hence $\widetilde{e}_\infty (\zeta _\infty \times \eta _\infty ,-j) \ne 0$.

### Symmetric square *p*-adic *L*-functions

To pin the term at infinity down more precisely, we exploit the fact that Theorem 9.5 is known in full when $\Pi$ is essentially self-dual, i.e. $\Pi$ is a (twist of a) symmetric square lift. We recall this result.

Let *f* be a classical cuspidal *p*-ordinary newform of weight $a+2$ and level *N*, and $\theta$ a finite order Hecke character over $\mathbb {Q}$ of prime-to-*p* conductor. Let $\pi ' = \textrm{Sym}^2(f) \times \theta$ be the symmetric square lift to $\textrm{GL}_3$, which has weight (2*a*, *a*, 0), and let  (which has weight $(a,0,-a)$).

The form *f* has a unique ordinary *p*-refinement; write $p^{1/2}A_p$ for its $U_p$-eigenvalue, which is a *p*-adic unit (so $v_p(A_p) = -1/2$). Then $\pi$ has an ordinary $P_1$-refinement defined by an unramified character $\sigma _p$, where ; as $\theta (p)$ is a root of unity, we have $v_p(\alpha _p) = -a-1$.

If $\omega _\pi (-1) = -1$, let $b = 0$; otherwise let $b=1$.

#### Theorem 9.6

(Schmidt, Hida, Dabrowski–Delbourgo, Rosso) There exists $\mathcal {L}_p^-(\pi ) \in \mathbb {C}_p\otimes _{\mathbb {Z}_p}\mathbb {Z}_p[\![\mathbb {Z}_p^{\times }]\!]$ such that for finite-order characters $\eta$ of $\mathbb {Z}_p^{\times }$, and $0 \le j \le a$ with $(-1)^j = \omega _\pi \eta (-1)$, we have

9.6 $$\begin{aligned} \int _{\mathbb {Z}_p^{\times }} \eta ^{-1}(x)x^{-j}\cdot \textrm{d}\mathcal {L}_p(\tilde{\pi })(x)= &   \frac{\omega _\pi (-1)\cdot \Gamma (-j+a+1)}{2^{2a+4}\cdot i^b \cdot (2\pi i)^{-j}} \cdot e_p(\pi _p \times \eta _p,-j)\nonumber \\  &   \cdot \frac{L^{(p)}(\pi \times \eta ,-j)}{\pi ^{a+1}\langle f,f\rangle }. \end{aligned}$$

#### Proof

This is summarised in [43, Thm. 2.3.2(i)], at least when $p\not \mid N$; the case where *p*||*N* is described in [55]. For the convenience of the reader we indicate how the statement in [43] translates to that above; the comparison with [55] follows as both are compatible with Conjecture 1.2(i), which has a uniform statement whether *p* divides *N* or not.

Since $L(\pi ,s) = L(\textrm{Sym}^2(f)\times \eta ,s+a+1)$ we renormalise, first defining $\mathcal {L}_p^-(\tilde{\pi })$ so that

$$ \int _{\mathbb {Z}_p^{\times }} f(x) \cdot \textrm{d}\mathcal {L}_p^-(\tilde{\pi }) = \int _{\mathbb {Z}_p^{\times }}x^{a+1}f(x)\cdot \textrm{d}L_p(\textrm{Sym}^2f \otimes \theta ) $$

(in the notation of [42]), and making the substitution $-j = s-a-1 = s-k+1$ (with $k = a+2$). Translating between the data attached to the refinements of *f* and $\pi$ then equates $G(\eta ) \cdot \mathcal {E}_p(s,\eta )$ *op. cit*. with $e_p(\pi _p \times \eta _p,-j)$ here. The parity condition *op. cit*. is $(-1)^{a+1-j}\eta (-1) = - \theta (-1)$; since $\omega _\pi (-1) = \omega _{\textrm{Sym}^2(f)}(-1)\theta (-1) = (-1)^a\theta (-1)$, this is equivalent to $(-1)^j = \omega _\pi \eta (-1)$. Combining all of this shows that $\int _{\mathbb {Z}_p^{\times }} \eta ^{-1}(x)x^{-j} \cdot \mathcal {L}_p^-(\tilde{\pi })(x)$ is equal to the right-hand side of (9.6). $\square$

#### Remark 9.7

The proof of Theorem 9.6 is rather circuitous, owing to complications with local Euler factors at the bad primes. Taking $\theta = 1$ for simplicity, the method initially interpolates values of the “imprimitive” symmetric square *L*-function

$$ L^{\textrm{imp}}(\operatorname {Sym}^2(f), s) = L^{(N_f)}(2\,s - 2a - 2, \omega _f^2) \cdot \sum _{n \ge 1} a_{n^2}(f) n^{-s}, $$

which differs from the “true” symmetric square *L*-function by a product of local error terms at the primes dividing $N_f$. Having constructed an imprimitive *p*-adic *L*-function, one can attempt to define a primitive *p*-adic *L*-function by dividing out by the error term; however, it remains to be shown that the resulting function does not have poles at the zeroes of the error term, and that it has the expected interpolation property at all $(j, \eta )$ in the interpolation range (even if the error term vanishes there, which can occur). This requires rather lengthy case-by-case analysis according to the local factors of *f* (see [23, §6] and [11, §3.1]).

However, for our present purpose of identifying the factors $\widetilde{e}_\infty (\zeta _\infty \times \eta _\infty ,-j)$, it suffices to know that there is a (possibly meromorphic) *p*-adic *L*-function which satisfies the conclusion of Theorem 10.3 for *almost all* $(j, \eta )$ in the appropriate range. This is much more straightforward to prove using the methods of [56]. Combining this partial result towards Theorem 9.6 with the output of our present construction, we obtain the full strength of Theorem 9.6 as a consequence, yielding an alternative proof not requiring the intricate local computations involved in the previous approach.

### Interpolation of *L*-values, part II

We are evaluating $\widetilde{e}_\infty (\zeta _\infty \times \eta _\infty ,-j)$. This term depends only on data attached to $\Pi _\infty$ and *j*, as (to be critical) $\eta _\infty = \omega _{\Pi ,\infty } \cdot \textrm{sgn}^j$.

Note in particular $\widetilde{e}_\infty (\zeta _\infty ,-j)$ does not depend on any data at *p*. We now exploit this, and existence of symmetric square 3-adic *L*-functions, to show:

#### Proposition 9.8

For any $0 \le j \le a$, we have

$$ \widetilde{e}_\infty (\zeta _\infty \times \omega _{\Pi ,\infty }\textrm{sgn}^j,-j) = (2\pi i)^{j} \cdot \tfrac{\Gamma (-j+a+1)}{\Gamma (a+1)} \cdot \widetilde{e}_\infty (\zeta _\infty \times \omega _{\Pi ,\infty },0). $$

#### Proof

To ease notation, we write $\widetilde{e}_\infty (\zeta _\infty ,-j) = \widetilde{e}_\infty (\zeta _\infty \times \omega _{\Pi ,\infty }\textrm{sgn}^j,-j)$.

From (2.3), for fixed *a* there are only two possibilities for $\Pi _\infty$; denote them by

#### Lemma 9.9

Let $a \in \mathbb {Z}_{\ge 0}$. For $\epsilon \in \{\pm \}$, there exists a RACAR $\pi ^{a,\epsilon }$ such that:

(i) $$\pi ^{a,\epsilon }_\infty = \Pi _{\infty }^{a,\epsilon }$$;
(ii) $\pi ^{a,\epsilon }_3$ is *B*-ordinary;
(iii) There exists an elliptic modular newform $f_a$ of weight $a+2$ and a character $\theta$ such that $\pi ^{a,\epsilon } \cong \textrm{Sym}^2(f_a)\otimes \theta ||\cdot ||^{-a}$.

#### Proof

First suppose $a = 0$ and $\epsilon = +$. Take $f_0$ to be the unique newform of level 15, trivial character and weight 2. This newform is 3-ordinary and not of CM-type, so  is the required RACAR.

By Hida theory, for any $a+2 \ge 2$ there exists a 3-ordinary newform $f_a$ of weight $a+2$ congruent to $f_0$ mod 3 (with trivial character if *k* is even, and quadratic character mod 3 if *k* is odd). Since the mod 3 Galois representation associated to $f_0$ is surjective, $f_a$ cannot be of CM-type, so lifts to a RACAR of $\textrm{GL}_3$. We may thus take .

Finally we take , for $\theta$ an odd finite order Hecke character unramified at 3. $\square$

Now we return to the proof of Proposition 9.8. The lemma implies that for our given $\Pi$, there exists a RACAR $\pi \cong \textrm{Sym}^2(f) \otimes \theta ||\cdot ||^{-a}$ such that $\Pi _\infty \cong \pi _\infty$ and $\pi _3$ is *B*-ordinary (hence $P_1$-ordinary). As $\widetilde{e}_\infty (\zeta _\infty ,-j)$ only depends on the factor at infinity, it suffices to work with $\pi$ rather than $\Pi$. Let $L_3^-(\pi )$ and $\mathcal {L}_3^-(\pi )$ be the 3-adic *L*-functions of Definition 9.3 and Theorem 9.6 respectively.

Note that $L_3^-(\pi )$ and $\mathcal {L}_3^-(\pi )$ interpolate the same *L*-values $L(\pi \times \eta ,-j)$, so when considered as (bounded) rigid analytic functions on weight space $\mathscr {W}$, they are supported on the same half of $\mathscr {W}$. In particular, we can make sense of $L_3^-(\pi )/\mathcal {L}_3^-(\pi ) \in \textrm{Frac}(\mathbb {C}_3\otimes _{\mathbb {Z}_3}\mathbb {Z}_3[\![\mathbb {Z}_3^{\times }]\!])$ as a well-defined meromorphic function on $\mathscr {W}$. This quotient is uniquely determined by its integral against finite-order characters $\eta$ of $\mathbb {Z}_3^{\times }$ such that $\eta (-1) = \omega _\pi (-1)$ (and vanishes when $\eta (-1) = - \omega _\pi (-1)$). By considering $j = 0$ in (9.5) and Theorem 9.6, we deduce that

$$ \int _{\mathbb {Z}_3^{\times }} \eta (x) \cdot \textrm{d}\frac{L_3^-(\pi )}{\mathcal {L}_3^-(\pi )} = \widetilde{e}_\infty (\zeta _\infty ,0) \cdot \left[ \frac{\omega _\pi (-1)\cdot \Gamma (a+1)}{2^{2a+4}\cdot i^b \cdot \pi ^{a+1}}\right] ^{-1} \cdot \frac{\langle f,f\rangle }{\Omega _\Pi ^-}. $$

As this is independent of $\eta$, the quotient $L_3^-(\pi )/\mathcal {L}_3^-(\pi )$ is *constant*, say equal to $C \in \mathbb {C}_3^{\times }$.

Now let $0 \le j \le a$, and $\eta$ such that $(-1)^j = \omega _\pi \eta (-1)$. By constancy, this is

$$\begin{aligned} C&= \int _{\mathbb {Z}_3^{\times }} \eta (x)x^{j}(x) \cdot \textrm{d}\frac{L_3^-(\pi )}{\mathcal {L}_3^-(\pi )}\\&= \widetilde{e}_\infty (\zeta _\infty ,-j)\cdot \left[ \frac{\omega _\pi (-1)\cdot \Gamma (-j+a+1)}{2^{2a+4}\cdot i^b \cdot (2\pi i)^{-j}\cdot \pi ^{a+1}}\right] ^{-1} \cdot \frac{\langle f,f\rangle }{\Omega _\Pi ^-}, \end{aligned}$$

where the second equality is the interpolation formula; and hence we have

9.7 $$\begin{aligned} \widetilde{e}_\infty (\zeta _\infty ,-j) = (2\pi i)^{j} \cdot \tfrac{\Gamma (-j+a+1)}{\Gamma (a+1)} \cdot \widetilde{e}_\infty (\zeta _\infty ,0), \end{aligned}$$

proving Proposition 9.8. $$\square $$

Now return to our original representation $\Pi$. We are free to renormalise $\zeta _\infty$ by any element of $\mathbb {C}^{\times }$; this then rescales $\Theta _\Pi$, $\Omega _\Pi ^-$, $P_j(\zeta _\infty ;s)$, and hence also $\widetilde{e}_\infty (\zeta _\infty \times \omega _{\Pi ,\infty }\textrm{sgn}^j,-j)$. We renormalise it so that $\widetilde{e}_\infty (\zeta _\infty \times \omega _{\Pi ,\infty },0) = e_\infty (\Pi _\infty \times \omega _{\Pi ,\infty },0).$ Since by definition

$$ e_\infty (\Pi _\infty \times \omega _{\Pi ,\infty }\textrm{sgn}^j,-j) = (2\pi i)^{j} \cdot \tfrac{\Gamma (-j+a+1)}{\Gamma (a+1)} \cdot e_\infty (\Pi _\infty \times \omega _{\Pi ,\infty },0), $$

from Proposition 9.8 we deduce

9.8 $$\begin{aligned} \widetilde{e}_\infty (\zeta _\infty \times \omega _{\Pi ,\infty }\textrm{sgn}^j,-j) = e_\infty (\Pi _\infty \times \omega _{\Pi ,\infty }\textrm{sgn}^j,-j). \end{aligned}$$

Theorem 9.5 follows by combining this with (9.5), recalling that if $(-j,\eta )$ is critical, then $\eta _\infty = \omega _{\Pi ,\infty }\textrm{sgn}^j$.

#### Remark 9.10

Combining the equality (9.8) with Theorem 2.7 also completes the proof of the Algebraicity Conjecture (Conjecture 1.1) for $n=3$ for $(-j,\eta ) \in {{\,\textrm{Crit}\,}}^-(\Pi )$; the analogous result for ${{\,\textrm{Crit}\,}}^+(\Pi )$ will be proved in the next section.

## Duality and functional equations

Our results so far have focused entirely on $P_1$-nearly-ordinary representations, and on interpolation of *L*-values $L(\Pi \times \eta ,-j)$ for $(-j,\eta ) \in {{\,\textrm{Crit}\,}}^-(\Pi )$, the left half of the critical range. In this section we complete the proof of Conjecture 1.2 by studying the mirror-image picture for $P_2$-nearly-ordinary refinements, for which we obtain interpolation over ${{\,\textrm{Crit}\,}}^+_p(\Pi )$, that is, in the right-half of the critical range.

Suppose $\Pi$ is a RACAR of $\textrm{GL}_3$ admitting a (necessarily unique) $P_2$-nearly-ordinary refinement $\sigma _p \times \sigma _p'$. Using the invariance of Conjecture 1.2 under twisting, as in Proposition 2.19, we may assume without loss of generality that $\sigma _p'$ is an unramified character.^3^ Hence the dual $\sigma _p^{\prime \vee }$ defines a $P_1$-ordinary refinement of $\Pi ^\vee$; and Theorem 9.5 thus yields a *p*-adic *L*-function $L_p^-\big (\Pi ^\vee \big ) \in \mathcal {O}_L[\![\mathbb {Z}_p^{\times }]\!]$.

### Definition 10.1

(i) If $C \in \mathbb {Z}_p^{\times }$, let $[C] \in \mathcal {O}[\![\mathbb {Z}_p^{\times }]\!]^{\times }$ denote the Dirac measure defined by
  $$ \int _{\mathbb {Z}_p^{\times }} f(x) \cdot \textrm{d}[C] = f(C). $$
(ii) Let $\operatorname {tw}_1$ be the unique automorphism of $\mathcal {O}_L[\![\mathbb {Z}_p^{\times }]\!]$ sending [*C*] to $C \cdot [C]$, and (as above) $\iota$ the involution sending [*C*] to $[C^{-1}]$, so
  $$ \int x^s\, \textrm{d}\iota (\operatorname {tw}_1 \mu ) = \int x^{1-s}\, \textrm{d}\mu $$
  for all $s \in \mathbb {Z}$ and $\mu \in \mathcal {O}_L[\![\mathbb {Z}_p^{\times }]\!]$.
(ii) Define the measure $L_p^+(\Pi )$ to be

Here $N_\Pi ^{(p)}$ is the prime-to-*p* part of the conductor of $\Pi$ (the integer such that the functional equation of the complex *L*-function involves a factor of $N_{\Pi }^{-s}$); the Dirac measure $[1/N_\Pi ^{(p)}]$ is a *p*-adic avatar of this factor. Let also 

### Proposition 10.2

The *p*-adic *L*-function $L_p^+(\Pi )$ satisfies the following interpolation property: for all $(j+1,\eta ) \in {{\,\textrm{Crit}\,}}^+(\Pi )$, we have

10.1 $$\begin{aligned} \int _{\mathbb {Z}_p^{\times }} \eta (x)x^{j+1} \cdot \textrm{d}L_p^+(\Pi )(x)&= e_\infty (\Pi _\infty \times \eta _\infty ,j+1)\cdot e_p\big (\Pi _p \times \eta _p^{-1}, j+1\big )\nonumber \\&\quad \cdot \frac{L^{(p)}(\Pi \times \eta ^{-1},j+1)}{\Omega _\Pi ^+}. \end{aligned}$$

### Proof

By Theorem 9.5, we have

$$\begin{aligned} \int _{\mathbb {Z}_p^{\times }} \eta (x)x^{j+1} \cdot \textrm{d}L_p^+(\Pi )(x)&= (N_{\Pi }^{(p)})^{-j}\cdot \eta (N_{\Pi }^{(p)})^{-1} \cdot \varepsilon ^{(p)}(\Pi ^{\vee ,(p)},0)^{-1}\\&\quad \times e_\infty (\Pi _\infty ^\vee \times \eta _\infty , -j)\cdot e_p(\Pi ^\vee _p \times \eta _p,-j)\\&\quad \cdot \frac{L^{(p)}(\Pi ^\vee \times \eta ,-j)}{\Omega _{\Pi ^\vee }^-}. \end{aligned}$$

In [9, (20)], Coates proves the equality

$$\begin{aligned} e_\infty (&\Pi _\infty ^\vee \times \eta _\infty ,-j) \cdot e_p(\Pi ^\vee _p \times \eta _p, -j)\cdot \frac{L^{(p)}(\Pi ^\vee \times \eta ,-j)}{\Omega _{\Pi ^\vee }^-}\\&=\Big (\prod _{\ell \ne p} \varepsilon _\ell (\Pi _\ell ^\vee \times \eta _\ell ,-j)\Big ) \cdot e_\infty (\Pi _\infty \times \eta _\infty ^{-1}, j+1)\cdot e_p(\Pi _p \times \eta _p^{-1},j + 1)\\&\quad \cdot \frac{L^{(p)}(\Pi \times \eta ^{-1},j+1)}{\Omega _{\Pi }^+}. \end{aligned}$$

Here we recall that the modified Coates–Perrin-Riou factors were set up to use the additive character $\psi$ for ${{\,\textrm{Crit}\,}}^-_p(\Pi ^\vee )$ and $\psi ^{-1}$ for ${{\,\textrm{Crit}\,}}^+_p(\Pi )$. As $\eta$ is unramified at $\ell \ne p$, as in [9] we can exploit [59, (3.4.6)] to see

$$ \varepsilon _\ell (\Pi _\ell ^\vee \times \widehat{\eta }_\ell ,-j) = \varepsilon _\ell (\Pi _\ell ^\vee ,0) \cdot \ell ^{jc_\ell }\cdot \widehat{\eta }_\ell ^{-1}(\ell ^{c_\ell }), $$

where $c_\ell$ is the exponent of $\ell$ in $N_{\Pi ^\vee } = N_{\Pi }$. In particular, we have

$$ \prod _{\ell \ne p} \varepsilon _\ell (\Pi _\ell ^\vee \times \widehat{\eta }_\ell ,-j) = \varepsilon ^{(p)}(\Pi ^{\vee ,(p)},0) \cdot (N_\Pi ^{(p)})^j \cdot \widehat{\eta }^{(p)}(N_\Pi ^{(p)})^{-1}. $$

We conclude by combining all terms, noting $\hat{\eta }^{(p)}(N_\Pi ^{(p)})^{-1} = \widehat{\eta }_p(N_\Pi ^{(p)})^{-1} = \eta (N_\Pi ^{(p)})$ via the conventions in Sect. 2.1. $\square$

With this, we have completed the proof of Theorem B.

## Data Availability

No new data were created during this study.

## Glossary of notation/terminology

*a*: Integer $\ge 0$; weight of $\Pi$ (Sect. 2.3.2)
$$\alpha _p = \sigma _p(p)$$: (For $$\sigma _p = P_1$$-refinement)
$B \subset \textrm{GL}_3$: Upper-triangular Borel
$\beta _p$: Satake parameter (Sect. 2.4)
$\textrm{br}^{[a,j]}$: Branching law (Sect. 4.2)
${{\,\textrm{Crit}\,}}_p^\pm (\Pi )$: Critical values (Sect. 2.5)
$c \in \mathbb {Z}$: Aux. integer prime to 6*p*
$\chi$: Dirichlet character (Sect. 2.1)
$\widehat{\chi }$: Hecke character (Sect. 2.1)
$$\Delta _n$$: $$(\mathbb {Z}/p^n\mathbb {Z})^{\times }$$
*E*: Suff. large number field (Sect. 3.3.1)
$E_\Phi$: Adelic Eisenstein series (Sect. 5.2.1)
$$E_\Phi ^{j+2,\chi }$$: (5.7)
$\mathcal {E}_{{\Phi _{\textrm{f}}}}^{j+2}$: Classical Eisenstein series (Sect. 5.2.2)
$$\mathcal {E}_{{\Phi _{\textrm{f}}}}^{j+2,\chi }$$: (5.6)
$\textrm{Eis}_{{\Phi _{\textrm{f}}}}^{j+2}$: Betti–Eisenstein class (Cor. 5.6)
Integral $$\textrm{Eis}_{{\Phi _{\textrm{f}}}}^{j+2}$$ (Thm. 5.7)
Eisenstein–Iwasawa class (Sect. 5.3.4)
$e_\infty (\Pi _\infty \times \eta _\infty ,t)$: Mod. Euler factor at $\infty$ (Sect. 2.5)
$\widetilde{e}_\infty (\zeta _\infty \times \eta _\infty ,t)$: Mahnkopf factor at $\infty$ (9.5)
$e_p(\Pi _p \times \eta _{p}, t)$: Mod. Euler factor at *p* (Sect. 2.7)
$\eta$: Dirichlet char. of cond. $p^n$
$\eta _2$: Dir. char., cond. *D* prime to *p* (Not. 6.5)
$G(\eta )$: Gauss sum (Sect. 2.1)
$\gamma _p$: Satake parameter (Sect. 2.4)
*H*: $$\textrm{GL}_2 \times \textrm{GL}_1$$
$I(-,-)$: Principal series for $\textrm{GL}_2$ (Sect. 5.2.1)
$$I_j(\widehat{\chi })$$: $$I(\Vert \cdot \Vert ^{-1/2}, \widehat{\chi }^{-1}\Vert \cdot \Vert ^{k+1/2})$$ (5.9)
$$\iota : H \hookrightarrow \textrm{GL}_3$$: (4.1)
$\mathcal {H}_J$: Symmetric space for *J* (Sect. 3.1)
*J*: General reductive group
*j*: Integer $$0 \le j \le a$$
$K_{\textrm{GL}_n,\infty } \subset \textrm{GL}_n(\mathbb {R})$: Max. cpct subgroup
$$K_{n,\infty }^\circ \subset \textrm{GL}_n(\mathbb {R})$$: $$= K_{\textrm{GL}_n,\infty }^\circ Z_{\textrm{GL}_n,\infty }^\circ $$
*L*: Suff. large *p*-adic field (Rem. 3.3)
$$\lambda = (a,0,-a)$$: Weight of $$\Pi $$
$N \subset \textrm{GL}_3$: Upper-triangular unipotent
$$N_{P_i}$$: Unipotent in $$P_i$$
$$\nu _1: H \rightarrow \textrm{GL}_1$$: $$(\gamma ,z) \mapsto \det (\gamma )/z$$
$$\nu _2: H \rightarrow \textrm{GL}_1$$: $$(\gamma ,z) \mapsto z$$
$$\nu _{1,(n)}: \widetilde{Y}^H \rightarrow \Delta _n$$: (6.2)
$$\Omega _\Pi ^-$$: (9.1)
$$\Omega _\Pi ^+$$: $$= \Omega _{\Pi ^\vee }^-$$ (Sect. 10)
$\widehat{\omega }_\Pi$: Central char. of $\Pi$
$P_1,P_2 \subset \textrm{GL}_3$: Max. standard parabolics (Sect. 2.6)
$$\Pi $$: RACAR of $$\textrm{GL}_3$$
$\Phi , {\Phi _{\textrm{f}}}$: Schwartz functions (Sect. 5.1)
$\Phi _{\textrm{f}}^t$: Specific Schwartz function (Sect. 5.3.4)
$$\phi _\Pi $$: Map $$\mathcal {W}_\psi (\Pi _{\textrm{f}}) \rightarrow \textrm{H}_{\textrm{c}}^{2}(-)$$ (Sect. 3.3)
$$\textrm{pr}_{\textrm{GL}_2}$$: Projection $$H \rightarrow \textrm{GL}_2$$
$\varphi \in \Pi$: Cusp form (in $L^2_0(\textrm{GL}_3(\mathbb {Q})\backslash \textrm{GL}_3(\mathbb {A}))$)
$\varphi _{\infty ,r,s,\alpha }$: Vectors at $\infty$ (Sect. 7.2.1)
$\psi$: Additive character of $\mathbb {Q}\backslash \mathbb {A}$ (Sect. 2.1)
RACAR: Regular alg. cusp. auto. rep.
$$R_\chi $$: $$\chi ^{-1}$$-projector (5.1)
$\mathcal {S},\mathcal {S}_0$: Spaces of Schwartz functions (Sect. 5.1)
$\textrm{tw}_j$: Twist by $\Vert \nu _1\Vert ^{-j}$ (Sect. 6.1.1)
$$\tau = \tau _1 \in \textrm{GL}_3(\mathbb {Q}_p)$$: $$\textrm{diag}(p,1,1)$$ (2.6)
$$\tau _2 \in \textrm{GL}_3(\mathbb {Q}_p)$$: $$\textrm{diag}(p,p,1)$$
$\Theta _\Pi$: Cohomological period (Sect. 3.3.1)
$U_{p,1},U_p$: (Normalised) Hecke ops. at *p* ((2.6), Sect. 3.2)
$U_{p,1}',U_p'$: (Normalised) adjoint Hecke ops. (Sect. 3.2)
$\mathcal {U}^J \subset J(\mathbb {A}_{\textrm{f}})$: Open compact level
$\mathcal {U}^{(p)}$: Prime-to-*p* level
$$\mathcal {U}_n \subset \textrm{GL}_3(\mathbb {Z}_p)$$: Def. 6.2
$$\mathcal {U}_1^H(p^t) \subset H(\mathbb {A}_{\textrm{f}})$$: (Sect. 6.1.2)
*u*: $$\left( {\begin{smallmatrix} 1 & 0 & 1 \\ & 1 & 0 \\ & & 1\end{smallmatrix}}\right) \left( {\begin{smallmatrix} 1 & & \\ & & -1 \\ & 1 & \end{smallmatrix}}\right) $$
$V_\lambda$: Highest weight rep. of $\textrm{GL}_3$ (Sect. 2.2)
$V_\mu ^J$: Highest weight rep. of *J*
$$\mathcal {V}_n \subset \textrm{GL}_3(\mathbb {Z}_p)$$: Def. 6.8
$\mathscr {V}_*^*$: Local system attached to $V_*^*$ (Sect. 3.2)
$\mathcal {W}_\psi$: Whittaker transform (Sect. 2.3.1)
*W*: Element in $$\mathcal {W}_\psi (\Pi )$$
$\textrm{H}^3(Y^{\textrm{GL}_3})$-valued measures (Sect. 6.2)
$$\Xi ^{[a,j]}_n, \Xi ^{[a,j]}$$: Measures (Sect. 6.3)
Def. 6.9
$Y^J(\mathcal {U})$: Loc. sym. space for *J* (§3.1)
$Y(-)$: Local zeta integral (Def. 8.3)
$\widetilde{Y}^H$: Modified space for *H* (4.2)
$$Z_{\textrm{GL}_n,\infty }$$: Centre of $$\textrm{GL}_n(\mathbb {R})$$
$Z(-)$: Local zeta integral (Def. 8.1)
$$\widetilde{Z}(-)$$: Modified $$Z^{k+2,\chi }$$ (Thm. 8.2)
$\mathcal {Z}(-)$: Global zeta integral (Def. 7.2)
Def. 6.6
$$\zeta _\infty $$: $$\in \textrm{H}^2(\mathfrak {gl}_3,K_{3,\infty }^\circ ; \Pi _\infty \otimes V_{\lambda }^{\vee }(\mathbb {C}) )$$ (2.2)
$\langle -,-\rangle _{\mathcal {U}}$: Poincaré duality pairing (Sect. 3.4)
$\langle -,-\rangle _{a,j}$: Branching law pairing (4.4)
$$(-)^\circ $$: Identity component

## A Proof of Theorem 8.4

We now give the proof of the “partial functional equation” relating the *Y* and *Z* integrals. Compare Proposition A.3 of [44], which is a closely related computation in the case of $\textrm{GL}_2 \times \textrm{GL}_2$, rather than $\textrm{GL}_3 \times \textrm{GL}_2$. Replacing $(\pi , \chi _1, \chi _2)$ with $(\pi \times \chi _1, \textrm{id}, \chi _2)$, we may suppose without loss of generality that $\chi _1 = \textrm{id}$.

### Remark A.1

The overall shape of the computation is as follows: the integral $Z(W, \Phi ; \chi _1, \chi _2, s_1, s_2)$ involves the function $W^{\Phi }(-; \chi _2, s_2)$, which lies in the Whittaker model of a $\textrm{GL}_2$ principal-series representation (depending on the parameters $\chi _2$ and $s_2$). Meanwhile, the *Y* integral involves $f^{\Phi }(-; \chi _2, s_2)$, which lies in the natural model of the $\textrm{GL}_2$ principal series (as a space of functions on $\textrm{GL}_2$ transforming by a character under left-translation by the Borel). There is an explicit intertwining operator from the natural induced model of the principal-series representation to its Whittaker model, sending $f^{\Phi }(-; \chi _2, s_2)$ to $W^{\Phi }(-; \chi _2, s_2)$; and we need to show that the composite of the *Z* integral and the intertwining operator agrees with the *Y* integral up to the constant factor $\gamma (\pi \times \chi _1/\chi _2, s_1 - s_2 + \tfrac{1}{2})$.

We note that both integrals define elements of the space $\textrm{Hom}_H(\pi \times \sigma , \mathbb {C})$, where $\sigma$ is a principal-series *H*-representation (depending on the parameters $s_i, \chi _i$). For sufficiently general values of $s_i$, the representation $\sigma$ is irreducible and generic, so this Hom-space has dimension 1, by a well-known multiplicity-one result (see the introduction of [52], where the result is attributed to J. Bernstein). Hence the two integrals must be proportional, and it remains only to identify the constant as a $\gamma$-factor.

We begin by recalling some $\textrm{GL}_3 \times \textrm{GL}_1$ zeta-integrals introduced by Jacquet et al. We let $F = \mathbb {Q}_p^{\times }$ (in fact any nonarchimedean local field would work here).

### Definition A.2

For $W \in \mathcal {W}(\Pi )$ we consider the integrals

$$ \Psi _0(W, \chi , s) = \int _{F^{\times }} W\left( \left( {\begin{smallmatrix}a \\   &  1\\   & &  1\end{smallmatrix}}\right) \right) \chi (a) |a|^{s-1}\ \textrm{d}^{\times }\!a$$

and

$$ \Psi _1(W, \chi , s) = \int _{F \times F^{\times }} W\left( \left( {\begin{smallmatrix} a &  &  \\ x &  1 &  \\ &  &  1\end{smallmatrix}}\right) \right) \chi (a) |a|^{s-1} \ \textrm{d}x\, \textrm{d}^{\times }\!a.$$

By [27, Thm. 2.7(i),(ii)], both integrals converge for $\Re (s) \gg 0$ and have meromorphic continuation to all *s*, and the greatest common divisor of the values of either integral is $L(\pi \times \chi , s)$. By part (iii) of the cited theorem, we also have a functional equation for $\Psi _j$; let us write

For $W \in \mathcal {W}(\pi )$, we write $\tilde{W}(g) = W(w_r {}^t\! g^{-1})$; the functions $\{ \tilde{W}: W \in \mathcal {W}(\pi , \psi )\}$ form the Whittaker model $\mathcal {W}(\pi ^\vee , \psi ^{-1})$. Then for $j = 0, 1$ we have the functional equation

A.1 $$\begin{aligned} \Psi _{(1-j)}(w_{3, 1} \cdot \tilde{W}, \chi ^{-1}, 1-s) = \gamma (\pi \times \chi , \psi , s) \cdot \Psi _j(W, \chi , s). \end{aligned}$$

We now write the torus integral $y(\cdots )$ (from Definition 8.5) in terms of $\Psi _0$:

with the $\chi _2(-1)$ arising from the change of variables $b \leftrightarrow -b$. Applying the functional equation (A.1) to the inner term, we can write this as

$$\begin{aligned}&y(W, \textrm{id}, \chi _2, s_1, s_2)(g) \\&\qquad \quad = \gamma (\pi \times \chi _2^{-1},\psi , s_1 - s_2 + \tfrac{1}{2})\chi _2(-1)|\det g|^{s_1-\tfrac{1}{2}} \int _a |a|^{s_1 + s_2 - 3/2}\\&\qquad \qquad \qquad \Psi _1\left( w_{3, 1} w_3 \left( {\begin{smallmatrix}a \\   &  1\\   & &  1\end{smallmatrix}}\right) w_{3, 1} g \cdot W, \chi _2^{-1}, s_1 - s_2 - \tfrac{1}{2}\right) \ \textrm{d}^{\times } a. \end{aligned}$$

The integral expands to

$$\begin{aligned} \int _a&|a|^{s_1 + s_2 - 3/2} \int _{b,x} |b|^{s_1 - s_2 - \tfrac{1}{2}} \chi _2(b)^{-1} W\left( \left( {\begin{smallmatrix} b &   &   \\ x &  1 &   \\  &   &  1\end{smallmatrix}}\right) w_{3, 1} w_3 \left( {\begin{smallmatrix}a \\   &  1\\   & &  1\end{smallmatrix}}\right) w_{3, 1} g\right) \ \textrm{d}x\, \textrm{d}^{\times }\! a\, \textrm{d}^{\times }\! b\\&= \int _{a,b,x} |a|^{s_1 + s_2 - 3/2} |b|^{s_1 - s_2 - \tfrac{1}{2}} \chi _2(b)^{-1} W\left( \iota (w_2 \left( {\begin{smallmatrix}1 &  x/b\\ & 1\end{smallmatrix}}\right) \left( {\begin{smallmatrix}a &  \\ & b\end{smallmatrix}}\right) g)\right) \ \textrm{d}x\, \textrm{d}^{\times } a\, \textrm{d}^{\times } b\\&= \int _{a,b,x} |a|^{s_1 + s_2 - 3/2} |b|^{s_1 - s_2 + \tfrac{1}{2}} \chi _2(b)^{-1} W\left( \iota (w_2 \left( {\begin{smallmatrix}1 &  x\\ & 1\end{smallmatrix}}\right) \left( {\begin{smallmatrix}a &  \\ & b\end{smallmatrix}}\right) g)\right) \ \textrm{d}x\, \textrm{d}^{\times } a\, \textrm{d}^{\times } b. \end{aligned}$$

By (8.1), integrating this against $f_{\Phi }$ gives $Y(\cdots )$. In this pairing, we obtain an inner integral over $B_2$ and an outer integral over $B_2 \backslash \textrm{GL}_2$; so this rearranges into

$$\begin{aligned}&Y(W, \Phi ; \textrm{id}, \chi _2, s_1, s_2) \\&\qquad = \chi _2(-1)\gamma (\pi \times \chi _2^{-1},\psi , s_1 - s_2 + \tfrac{1}{2}) \int _{\textrm{GL}_2(F)} W(\iota (g))f_{\Phi }(w_2 g) |\det g|^{s_1-\tfrac{1}{2}}\ \textrm{d}g. \end{aligned}$$

Splitting into an integral over $N_2$ and an integral over $N_2 \backslash \textrm{GL}_2$, the $N_2$ factor acts on *W* via $\psi$, so the integral over $\textrm{GL}_2$ equals

$$ \int _{N_2 \backslash \textrm{GL}_2(F)} W(\iota (g)) |\det g|^{s_1-\tfrac{1}{2}} \left( \int _F f_{\Phi }(w_2 \left( {\begin{smallmatrix}1 &  x\\ 0& 1\end{smallmatrix}}\right) \,g; \chi _2, s_2) \psi (x)\ \textrm{d}x\right) \textrm{d}g. $$

Since $$w_2 = \left( {\begin{smallmatrix}1 &  \\  &  -1\end{smallmatrix}}\right) \left( {\begin{smallmatrix} &  1\\ -1 &  \end{smallmatrix}}\right) $$, and $f_{\Phi }$ transforms as $\chi _2(-1)$ under $$\left( {\begin{smallmatrix}1 &  \\  &  -1\end{smallmatrix}}\right) $$, by [37, §8.1] the second integral is precisely $\chi _2(-1)W_\Phi (g;\chi _2,s_2)$. The $\chi _2(-1)$’s cancel; we conclude since

$$\begin{aligned} Y(W, \Phi ; \textrm{id}, \chi _2, s_1, s_2)&= \gamma (\pi \times \chi _2^{-1},\psi , s_1 - s_2 + \tfrac{1}{2})\\&\hspace{20pt} \times \int _{N_2 \backslash \textrm{GL}_2(F)} W(\iota (g)) W_\Phi (g; \chi _2,s_2) |\det g|^{s_1-\tfrac{1}{2}}\textrm{d}g\\&= \gamma (\pi \times \chi _2^{-1},\psi , s_1 - s_2 + \tfrac{1}{2}) Z(W, \Phi ; \textrm{id}, \chi _2, s_1, s_2). \end{aligned}$$

## Footnotes

1 The case $n=2m$ is similar, but in many ways simpler: only the (*m*, *m*) parabolic subgroup is relevant; and $L(\Pi \times \eta , j)$ is critical for all *j* in a certain interval, independently of the parity of $\eta$, so we do not need to split the critical interval into the two halves ${{\,\textrm{Crit}\,}}^{\pm }$.

2 Any RACAR of $\textrm{GL}_3(\mathbb {A})$ is unitary up to some twist by an integral power of the norm character, so this assumption is harmless. This contrasts with the situation for $\textrm{GL}_2(\mathbb {A})$, where the analogous statement fails.

3 We might call such refinements “co-ordinary”.
